# Design, Synthesis, and Biological Evaluation of 6″-Modified Apramycin Derivatives to Overcome Aminoglycoside Resistance

**DOI:** 10.3390/pharmaceutics17121583

**Published:** 2025-12-08

**Authors:** Kseniya S. Shapovalova, Georgy V. Zatonsky, Elizaveta A. Razumova, Nikolai D. Dagaev, Dmitrii A. Lukianov, Natalia E. Grammatikova, Alexander S. Tikhomirov, Andrey E. Shchekotikhin

**Affiliations:** 1 Gause Institute of New Antibiotics, 11 B. Pirogovskaya Street, 119021 Moscow, Russia; schapowalowa.kseniya2014@yandex.ru (K.S.S.); gzatonsk@gmail.com (G.V.Z.); ngrammatikova@yandex.ru (N.E.G.); tikhomirov@gause-inst.ru (A.S.T.); 2 Department of Chemistry, Lomonosov Moscow State University, Leninskie Gory 1, 119991 Moscow, Russia; elizaveta_razumova@list.ru (E.A.R.); lukianovda@my.msu.ru (D.A.L.); 3 A.N. Belozersky Institute of Physico-Chemical Biology, Lomonosov Moscow State University, 119991 Moscow, Russia; nikolas.dagaev@yandex.ru; 4 Faculty of Bioengineering and Bioinformatics, Lomonosov Moscow State University, 119991 Moscow, Russia; 5 Center for Molecular and Cellular Biology, 121205 Moscow, Russia

**Keywords:** antibiotic resistance, aminoglycosides, apramycin, chemical transformation, semisynthetic antibiotics, antibacterial activity, translation inhibition, citotoxicity

## Abstract

**Background/Objectives**: Despite their long history of clinical use, aminoglycosides remain important broad-spectrum antibiotics, exhibiting potent activity against Gram-positive, Gram-negative, and mycobacterial pathogens. However, the growing prevalence of antimicrobial resistance, along with the well-documented nephrotoxicity and ototoxicity associated with this class, underscores the urgent need for novel derivatives with enhanced pharmacological and safety profiles. **Methods**: In this study, we developed a synthetic approach for the synthesis of new apramycin derivatives featuring structural modifications at the 6″-position of 4-amino-4-deoxy-D-glucose residue, specifically through the introduction of aminoalkylamine and guanidinoalkylamine substituents. The synthesized compounds were evaluated for their antimicrobial activity against a broad panel of bacterial strains, including multidrug-resistant clinical isolates. **Results**: The obtained derivatives of apramycin demonstrated significant antibacterial activity, retaining potency against strains resistant to conventional aminoglycosides. Moreover, the new compounds exhibited the ability to circumvent aminoglycoside resistance mediated by enzymatic modification and showed reduced cytotoxicity in mammalian cell assays. **Conclusions**: The distinctive pharmacological properties of apramycin and its newly synthesized derivatives, particularly their resilience to common resistance mechanisms and low cytotoxicity, highlight apramycin as a valuable structural scaffold for the development of next-generation aminoglycoside antibiotics with improved efficacy and safety.

## 1. Introduction

The overuse and misuse of antibiotics in medicine, agriculture, and animal husbandry have fueled a global antibiotics crisis, undermining the effectiveness of life-saving drugs. This has accelerated the development of antimicrobial resistance (AMR), leading to the rise in multidrug-resistant pathogens that threaten modern healthcare. According to the World Health Organization (WHO), a 2017 report identified 12 priority pathogens exhibiting resistance to multiple classes of antibiotics [1]. By 2024, this number had increased to 15, reflecting the accelerating pace at which resistant strains are emerging [2]. A striking feature of these pathogens is that the majority are Gram-negative bacteria, which present unique challenges due to their complex cell wall structure and ability to acquire and transfer resistance genes. Many of these strains exhibit multidrug resistance (MDR), significantly limiting available treatment options. Notably, the 2024 report also documented the appearance of the first *Mycobacterium tuberculosis* strain resistant to rifampicin, one of the cornerstone drugs for tuberculosis (TB) therapy, signaling a critical threat to global TB control programs.

**Figure 1:**
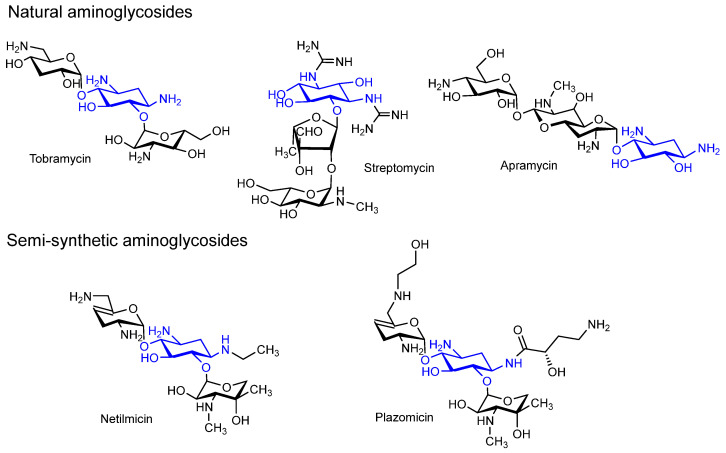
Examples of natural and semisynthetic aminoglycoside antibiotics.

Among the earliest antibiotics to be discovered were aminoglycosides (AG, Figure 1), which remain clinically important nowadays. These compounds possess a broad spectrum of activity against Gram-positive (G^+^) and Gram-negative (G^−^) bacteria, as well as mycobacteria [3]. Their mechanism of action, which involves binding to the bacterial 30S ribosomal subunit to disturb protein synthesis, makes them particularly valuable in treating severe infections, including sepsis, endocarditis, and drug-resistant tuberculosis. Despite their clinical utility, the use of aminoglycosides over the past decades has inevitably led to the emergence of resistance, mediated through diverse mechanisms such as enzymatic modification, ribosomal target alteration, and reduced drug uptake [4].

The persistence of AGs in clinical practice underscores both their therapeutic importance and the challenges posed by resistance. The current situation highlights the need for new strategies, including the development of next-generation derivatives with improved pharmacological properties, combination therapies to minimize resistance development, and novel adjuvants that can restore aminoglycoside efficacy. In parallel, global stewardship initiatives, stricter regulation of antibiotic use, and investment in surveillance systems remain critical to slowing the spread of resistance and safeguarding the effectiveness of antibiotics used in clinics and veterinary settings.

Apramycin is a natural antibiotic belonging to the aminoglycoside class. Unlike most natural and semi-synthetic AGs, its structural features differ markedly, particularly through the attachment of a monosubstituted 2-deoxystreptamine to a disaccharide containing bicyclic dialdose with a displaced binding site in the 30S ribosome (Figure 1) [5]. This unique structural configuration sets it apart from the classical 4,6-substituted AGs and plays a crucial role in both its antibacterial activity and resistance profile.

Originally, apramycin was developed and widely used as a veterinary drug. However, recent evidence has prompted renewed interest in its clinical potential for human medicine. A phase I clinical trial (NCT04105205) demonstrated that apramycin was safe in humans, with full results pending publication. Another ongoing phase I trial in the United States (NCT05590728) is assessing its pharmacokinetics in lung epithelial lining fluid and serum, aiming to evaluate its potential for the treatment of respiratory infections. Apramycin has exhibited potency against multidrug-resistant Gram-negative pathogens [6,7,8] and mycobacteria, including strains resistant to conventional treatment regimens [9,10]. Although AGs are often associated with significant oto- and nephrotoxicity [11], apramycin has demonstrated a comparatively favorable safety profile [12]. In preclinical studies, renal toxicity in rat models was significantly lower compared to gentamicin [13]. Similarly, in explanted cochlear models, apramycin did not appear to induce ototoxicity, possibly due to its reduced affinity for mitochondrial and eukaryotic ribosomes.

**Figure 2:**
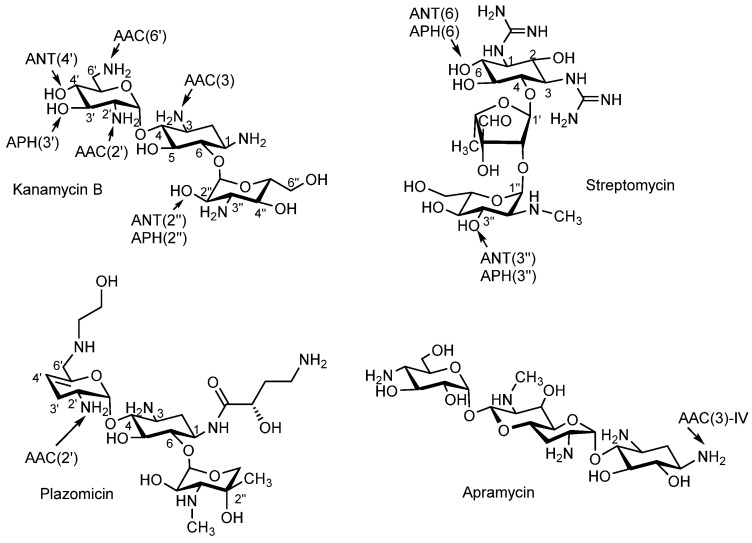
Action of aminoglycoside-modifying enzymes on natural and semi-synthetic aminoglycosides.

Aminoglycoside resistance is most commonly mediated by aminoglycoside-modifying enzymes (AMEs), which inactivate drugs by chemical modification (Figure 2) [14]. Three major families of AMEs have been described: acetyltransferases (ACC), which acetylate amino groups; phosphotransferases (APH), which phosphorylate hydroxyl groups; and adenylyltransferases (ANT), which adenylylate hydroxyl groups. These enzymatic processes render most AGs inactive. Notably, chemical modification of natural aminoglycosides can yield antibiotics that are resistant to inactivation by AMEs. A striking example is plazomicin, a semisynthetic derivative of sisomicin, which remains stable against numerous AMEs produced by multidrug-resistant bacteria (Figure 2) [15]. Apramycin similarly demonstrates intrinsic resistance to many AMEs, thereby evading the most prevalent aminoglycoside resistance mechanisms [16]. Moreover, its broad-spectrum antibacterial activity further underscores the potential of apramycin as a promising candidate in the fight against antimicrobial resistance.

Despite its clinical promise, only a limited number of chemical modifications of apramycin have been reported to date. Among them, the synthesis of apramycin 5-*O*-glycosides and ethers has shown improved antibacterial activity [17]. However, the rapid evolution of resistance among bacterial pathogens necessitates continued efforts to develop novel apramycin derivatives. In the present work, we describe the synthesis of apramycin derivatives modified at the 6″-position. This site was selected for several reasons: (i) it contains the only primary hydroxyl group, providing a high degree of reaction selectivity, and (ii) it does not participate directly in ribosomal binding, minimizing the risk of compromising antibacterial activity. We further evaluated the antibacterial properties of these derivatives against control strains and clinically relevant resistant pathogens, as well as cytotoxicity. Additionally, the mechanism of antibacterial action was evaluated.

## 2. Materials and Methods

### 2.1. Chemistry

#### Instruments, General Information, and Synthetic Procedures

Apramycin sulfate was sourced from Vostok Ltd. (Omutninsk, Russia). Unless otherwise specified, all reagents and solvents were obtained from commercial suppliers and were used without additional purification. Pyridine was dried over KOH, then over CaCl_2_, and distilled prior to use. Thin-layer chromatography (TLC) was performed on silica gel 60F_254_ plates (Merck, Darmstadt, Germany), and column chromatography was carried out using silica gel 60 (0.040–0.063 mm, Merck, Darmstadt, Germany). Visualization of apramycin and its derivatives on TLC plates was achieved using iodine vapor or 6N sulfuric acid, followed by heating; UV-active compounds were additionally detected under ultraviolet light (254 nm). All extracts were dried over anhydrous sodium sulfate and concentrated under reduced pressure at temperatures below 40 °C. Analytical high-performance liquid chromatography (HPLC) analysis was conducted on an LC-20AD system (Shimadzu, Kyoto, Japan) equipped with a UV detector and a Kromasil-100-C18, 4.6 mm × 250 mm column (Fisher Scientific, Pittsburgh, PA, USA), at a flow rate of 1 mL/min using the following mobile phase systems: System A1: A (0.2% aqueous ammonium formate, pH 4.5) and B (acetonitrile), with a linear gradient of acetonitrile from 40% to 90% over 30 min; System A2: A (water) and B (acetonitrile), with a linear gradient of acetonitrile from 80% to 95% over 30 min; and System A3: A (0.2% aqueous ammonium formate, pH 4.5) and B (acetonitrile), with a linear gradient of acetonitrile from 40% to 95% over 30 min. The purity of UV-absorbing intermediates was determined to be at least 90% by HPLC. Derivatives of apramycin were purified with reversed-phase chromatography on SepaBean machine T flash chromatograph (Santai, Jaingsu, China) on cartridge sepaflash spherical C18 (Santai, Jaingsu, China). ^1^H and ^13^C NMR spectra were recorded in DMSO-*d*_6_ or D_2_O solutions at 25 °C on a Bruker AV III 500 MHz spectrometer (Bruker Biospin AG, Fällanden, Switzerland), working at 500.2 MHz and 125.8 MHz, respectively. Signals were assigned using previously described methods [18,19]. High-resolution mass spectra (HRMS) were registered using electrospray ionization (ESI) on a Bruker Daltonics microOTOF-QII instrument. Elemental analysis was performed on a PerkinElmer 2400 CHN automatic microanalyzer (PerkinElmer, Inc., Waltham, MA, USA).

*2,6,4′,6′,4″-(Penta-N-Cbz)-apramycin (**2**).* To a solution of apramycin monosulfate (**1**, 1.50 g, 2.35 mmol) in a saturated aqueous solution of Na_2_CO_3_ (20 mL) at 0 °C, a solution of Cbz-Cl (2.0 mL, 2.4 g, 14.3 mmol) in acetone (8.5 mL) was added dropwise, maintaining the temperature at 0 °C. The reaction mixture was stirred with cooling for 2 h and then for 12 h at room temperature. Completion of the reaction was controlled by TLC. White precipitate was filtered off, then suspended in aqueous HCl (1 N, 50 mL), stirred for 30 min, and filtered off again. The crude solid was reprecipitated with H_2_O (50 mL) from DMSO (5 mL), the precipitate was filtered off, washed with water (3 × 10 mL), and dried under vacuum over P_2_O_5_. The yield of compound **2** was 2.45 g (86%) as a light beige solid. R_f_ = 0.45 (CHCl_3_:MeOH = 13:1), and HPLC (system A1) *t*_R_ = 17.6 min. HRMS (ESI): calculated for [C_61_H_75_N_6_O_21_]^+^ = 1227.498 0, found [M + NH_4_]^+^ = 1227.4946. ^1^H NMR (DMSO-*d*_6_; δ, ppm; *J*, Hz): 1.39–3.41 (m, 7H), 1.70–5.21 (m, 9H), 3.00 (s, 3H), 3.18–5.17 (m, 7H), 4.97–5.23 (m, 10H), 7.10 (s, 1H), 7.15 (s, 1H), 7.21–7.41 (m, 25H), 7.28 (s, 1H), 7.32 (s, 1H). ^13^C NMR (DMSO-*d*_6_; δ, ppm): 29.7, 32.2, 34.5, 50.1, 50.3, 51.2, 53.6, 58.0, 61.1, 65.1, 65.3, 66.2, 66.2, 69.4, 69.5, 70.3, 72.0, 72.8, 73.9, 76.9, 82.5, 93.4, 95.2, 97.9, 127.2, 127.6, 128.2, 128.5, 128.7, 136.9, 137.1, 137.2, 137.7, 155.4, 155.7, 155.9, 155.9, 156.5. Full ^1^H and ^13^C NMR signal assignments are available in the Supporting Information.

*2,6,4′,6′,4″-(Penta-N-Cbz)-6″-O-(2,4,6-triisopropylbenzosulfonyl)apramycin (**3a**) and 2,6,4′,6′,4″-(Penta-N-Cbz)-2″,6″-(di-O-(2,4,6-triisopropylbenzosulfonyl))apramycin (**3b**)*. To a solution of 2,6,4′,6′,4″-(penta-*N*-Cbz)-apramycin (**2**, 260 mg, 0.41 mmol) and 4-(dimethylamino)pyridine (113 mg, 0.93 mmol) in dry pyridine (3 mL) was added 2,4,6-triisopropylbenzenesulfonyl chloride (281 mg, 0.93 mmol). The reaction mixture was stirred for 6 h at room temperature, then an additional 2,4,6-triisopropylbenzenesulfonyl chloride (281 mg, 0.93 mmol) was added and stirred for 20 h. Completion of the reaction was controlled by TLC. The reaction was quenched by the addition of aqueous HCl (1 N, 10 mL) and H_2_O (10 mL). The product was extracted with ethyl acetate (3 × 20 mL), the extracts were combined, dried over Na_2_SO_4,_ and evaporated to dryness. The crude residue was purified by column chromatography on silica gel eluting with chloroform (200 mL), bringing the gradient to a mixture of chloroform and methanol (50:1) (200 mL). Fractions containing products **3a** and **3b** were combined and evaporated to dryness to obtain a product mixture, which was then separated by reversed-phase chromatography (gradient from 10% to 75% over 10 CV, then from 75% to 100% over 15 CV, water–acetonitrile system); fractions containing the products were combined and evaporated to dryness.

The yield of compound **3a** is 120 mg (20%) as a white solid. R_f_ = 0.6 (CHCl_3_:MeOH = 13:1), HPLC (system A2) *t*_R_ = 12.9 min. HRMS (ESI): calculated for [C_76_H_97_N_6_O_23_S]^+^ = 1493.6320, found [M + NH_4_]^+^ = 1493.6291. ^1^H NMR (DMSO-*d*_6_; δ, ppm; *J*, Hz): 1.10–1.16 (m, 18H), 1.41–3.44 (m, 7H), 1.70–5.13 (m, 9H), 2.79 (s, 2H), 3.00 (s, 3H), 3.58–5.48 (m, 7H), 4.57 (s, 1H), 4.78–5.27 (m, 10H), 6.83 (s, 1H), 6.95 (s, 2H), 7.11–7.41 (m, 25H), 7.14 (s, 1H), 7.28 (s, 1H), 7.42 (s, 1H). ^13^C NMR (DMSO-*d*_6_; δ, ppm): 23.8, 24.8, 27.9, 29.9, 31.9, 33.2, 34.4, 34.5, 50.4, 50.4, 51.5, 57.2, 58.3, 65.4, 66.1, 66.2, 66.4, 67.9, 69.1, 69.6, 70.0, 70.3, 74.2, 76.7, 76.9, 82.7, 95.0, 95.6, 98.0, 123.4, 123.4, 123.5, 123.7, 123.8, 123.8, 127.2, 127.5, 127.7, 128.1, 128.3, 128.4, 128.6, 136.8, 136.9, 137.0, 137.1, 137.2, 153.8, 155.1, 155.4, 155.7, 155.8, 156.3. Full ^1^H and ^13^C NMR signal assignments are available in the Supporting Information.

The yield of compound **3b** is 90 mg (15%) as a white solid. R_f_ = 0.7 (CHCl_3_:MeOH = 13:1), HPLC (system A2) *t*_R_ = 28.6 min. HRMS (ESI): calculated for [C_91_H_119_N_6_O_25_S_2_]^+^ =1759.7661, found [M + NH_4_]^+^ = 1759.7694. ^1^H NMR (DMSO-*d*_6_; δ, ppm; *J*, Hz): 1.16–1.24 (m, 36H), 1.37–3.42 (m, 7H), 1.58–5.14 (m, 9H), 2.98 (s, 3H), 3.30–5.40 (m, 7H), 4.02–4.12 (m, 6H), 4.94–5.15 (m, 10H), 6.69 (s, 1H), 6.92 (s, 1H), 7.16 (s, 1H), 7.21 (s, 2H), 7.21–7.38 (m, 25H), 7.28 (s, 1H), 7.32 (s, 2H). ^13^C NMR (DMSO-*d*_6_; δ, ppm): 23.2, 23.2, 29.1, 29.1, 29.1, 29.1, 29.6, 31.8, 34.4, 34.4, 34.7, 50.5, 50.5, 51.2, 54.4, 58.1, 65.1, 65.4, 65.9, 66.1, 66.2, 66.2, 68.6, 69.5, 69.6,70.4, 73.9, 76.8, 77.5, 83.7, 93.2, 93.8, 98.8, 123.4, 123.4, 123.4, 123.4, 123.5, 123.8, 123.8, 123.8, 123.8, 123.9, 127.2, 127.7, 128.3, 136.8, 137.2, 137.5, 149.9, 150.4, 153.1, 153.9, 155.4, 155.8. Full ^1^H and ^13^C NMR signal assignments are available in the Supporting Information.

*6″-((2-Aminoethyl-1)-amino)-2,6,4′,6′,4″-(penta-N-Cbz)-6″-deoxyapramycin (**4a**)*. Compound **3a** (500 mg, 0.34 mmol) was dissolved in ethylenediamine (3 mL) and stirred at room temperature for 24 h. Completion of the reaction was controlled by TLC. The reaction mixture was diluted with H_2_O (50 mL), the resulting precipitate was filtered off and washed with H_2_O (3 × 10 mL). A crude product was further purified by reversed-phase chromatography (gradient from 5% to 40% over 15 CV, then from 40% to 50% over 8 CV, ammonium acetate pH = 5-acetonitrile system), fractions containing the product were combined and evaporated to a volume of 2–3 mL, alkalinized with an aqueous solution of NH_4_OH to pH = 8, the resulting precipitate was filtered and dried under vacuum at 45 °C and then under vacuum over P_2_O_5_. The yield of compound **4a** is 150 mg (35%) as a white solid. R_f_ = 0.37 (CHCl_3_:MeOH:HCOOH 13:5:0.1), HPLC (system A3) *t*_R_ = 11.3 min. HRMS (ESI): calculated for [C_63_H_78_N_7_O_20_]^+^ = 1252.5296, found [M + H]^+^ = 1252.5286. ^1^H NMR (DMSO-*d*_6_; δ, ppm; *J*, Hz): 1.38–3.41 (m, 7H), 1.69–5.19 (m, 9H), 2.44 (s, 2H), 2.49 (s, 2H), 2.52–5.16 (m, 7H), 3.03 (s, 3H), 4.70–5.27 (m, 10H), 7.09 (s, 1H), 7.13 (s, 1H), 7.20–7.40 (m, 25H), 7.25 (s, 1H), 7.27 (s, 1H). ^13^C NMR (DMSO-*d*_6_; δ, ppm): 29.7, 32.2, 34.5, 41.2, 50.1, 50.3, 50.3, 51.2, 52.3, 54.9, 58.0, 65.0, 65.1, 65.2, 66.2, 66.3, 69.5, 69.5, 70.3, 71.6, 72.0, 73.9, 76.9, 82.5, 93.6, 95.2, 97.9, 127.2, 127.5, 127.6, 128.1, 128.2, 137.0, 137.1, 137.2, 137.7, 155.4, 155.5, 155.7, 155.9, 156.3. Full ^1^H and ^13^C NMR signal assignments are available in the Supporting Information.

*6″-((3-Aminopropyl-1)amino)-2,6,4′,6′,4″-(penta-N-Cbz)-6″-deoxyapramicyn (**4b**)*. Compound **4b** was prepared from **3a** and propan-1,3-diamine as described for **4a**. White powder, yield 52%. R_f_ = 0.35 (CHCl_3_:MeOH:HCOOH 13:5:0.1), HPLC (system A3) *t*_R_ = 8.7 min HRMS (ESI): calculated for [C_64_H_80_N_7_O_20_]^+^ = 1266.5453, found [M + H]^+^ = 1266.5399. ^1^H NMR (DMSO-*d*_6_; δ, ppm; *J*, Hz): 1.39–3.41 (m, 7H), 1.69–5.19 (m, 9H), 2.46 (s, 2H), 2.52 (s, 2H), 2.52–5.16 (m, 7H), 2.55 (s, 2H), 3.03 (s, 3H), 4.88–5.24 (m, 10H), 7.08–7.40 (m, 25H), 7.10 (s, 1H), 7.14 (s, 1H), 7.26 (s, 1H), 7.26 (s, 1H). ^13^C NMR (DMSO-*d*_6_; δ, ppm): 29.7, 32.2, 34.5, 39.6, 47.4, 50.1, 50.3, 50.3, 50.7, 51.2, 55.0, 58.0, 65.1, 65.2, 66.2, 66.3, 69.5, 69.5, 70.3, 71.6, 72.0, 73.9, 76.9, 82.5, 93.6, 95.2, 97.9, 127.2, 127.5, 127.6, 128.2, 137.1, 137.2, 137.7, 155.4, 155.7, 155.9, 156.3. Full ^1^H and ^13^C NMR signal assignments are available in the Supporting Information.

*6″-((2-Aminoethyl-1)-amino)-6″-deoxyapramycin acetate (**5a**)*. 6″-(2-Aminoethyamino)-2,6,4′,6′,4″-(penta-*N*-Cbz)-6″-deoxyapramycin (**4a**, 50 mg, 0.04 mmol) was dissolved in methanol (2 mL), and Pd on carbon (5 mass. %, 70 mg) was added to the solution. Acetic acid was added to the mixture until pH = 3 was reached, and the mixture was vigorously stirred at H_2_ flow (22 psi) for 4 h at room temperature. Completion of the reaction was controlled by TLC. The catalyst was filtered off via celite pad; the filter cake was washed with methanol (5 mL) and H_2_O (5 mL), and the combined filtrate was concentrated under vacuum. The target compound was precipitated with a mixture of isopropyl alcohol (5 mL) and acetone (20 mL), filtered off, and dried under vacuum over P_2_O_5._ The yield of compound **5a** was 21 mg (60%) as a white solid. R_f_ = 0.38 (NH_4_OH:iPrOH 10:7). HRMS (ESI): calculated for [C_23_H_48_N_7_O_10_]^+^ = 582.3457, found [M + H]^+^ = 582.3447. ^1^H NMR (DMSO-*d*_6_; δ, ppm; *J*, Hz): 1.94–3.65 (m, 7H), 1.97 (s, 15H), 2.03–5.57 (m, 9H), 2.71 (s, 3H), 2.88–5.51 (m, 7H), 3.03 (s, 2H), 3.20 (s, 2H). ^13^C NMR (DMSO-*d*_6_; δ, ppm): 26.2, 30.9, 33.6, 34.3, 41.0, 48.7, 51.2, 51.6, 52.2, 53.2, 56.9, 63.1, 66.6, 69.2, 72.7, 73.4, 73.8, 74.1, 76.3, 78.2, 86.2, 96.7, 97.3, 99.5, 184.2. Elem. anal.: calculated for [C_23_H_47_N_7_O_10_ × 5AcOH × 3H_2_O]: C, 42.35; H, 7.86; N, 10.48. Found: C, 42.68; H, 8.18; N, 10.13. Full ^1^H and ^13^C NMR signal assignments are available in the Supporting Information.

*6″-((3-Aminopropyl-1)-amino)-6″-deoxyapramycin acetate (**5b**)*. Compound **5b** was prepared from **4b** as described for **5a**. White solid, yield 40%. R_f_ = 0.37 (NH_4_OH:iPrOH 10:7). HRMS (ESI): calculated for [C_24_H_50_N_7_O_10_]^+^ = 596.3614, found [M + H]^+^ = 596.3674. ^1^H NMR (DMSO-*d*_6_; δ, ppm; *J*, Hz): 1.64–3.64 (m, 7H), 1.97 (s, 15H), 2.02–5.58 (m, 9H), 2.11 (s, 2H), 2.71 (s, 3H,), 2.75–5.46 (m, 7H), 3.10 (s, 2H), 3.13 (s, 2H). ^13^C NMR (DMSO-*d*_6_; δ, ppm): 26.1, 27.1, 30.8, 33.7, 34.2, 40.9, 48.1, 51.2, 51.6, 51.9, 53.2, 57.3, 63.2, 66.5, 69.1, 72.8, 73.1, 73.4, 74.5, 76.3, 78.2, 86.1, 97.3, 97.3, 99.4, 184.3. Elem. anal.: calculated for [C_24_H_49_N_7_O_10_ × 5AcOH × 3H_2_O]: C, 42.99; H, 7.96; N, 10.32. Found: C, 43.35; H, 8.22; N, 10.03. Full ^1^H and ^13^C NMR signal assignments are available in the Supporting Information.

*6″-((2-Aminoethyl-1)-amino)-2,6,4′,6′,4″-(penta-N-Cbz)-2″-O-(2,4,6-triisopropylbenzosulfonyl)-6″-deoxyapramycin (**6**)*. Compound **3b** (160 mg, 0.03 mmol) was dissolved in ethylenediamine (1.0 mL) and stirred at room temperature for 24 h. Completion of the reaction was controlled by TLC. The reaction mixture was diluted with H_2_O (50 mL), the forming precipitate was filtered off, washed with H_2_O (3 × 10 mL), and dried under vacuum at 45 °C and then under vacuum over P_2_O_5_. The yield of compound **6** was 110 mg (79%) as a white solid. R_f_ = 0.45 (CHCl_3_:MeOH:HCOOH 13:5:0.1). HRMS (ESI): calculated for [C_78_H_100_N_7_O_22_S]^+^ = 1518.6637, found [M + H]^+^ = 1518.6676. ^1^H NMR (DMSO-*d*_6_; δ, ppm; *J*, Hz): 1.12–1.27 (m, 18H), 1.39–3.43 (m, 7H), 1.59–5.18 (m, 9H), 2.56 (s, 2H), 2.58–5.44 (m, 7H), 2.62 (s, 2H), 2.93 (s, 2H), 3.04 (s, 3H), 4.13 (s, 1H), 4.96–5.08 (m, 10H), 6.95 (s, 1H), 7.16 (s, 1H), 7.22–7.36 (m, 25H), 7.30 (s, 2H), 7.34 (s, 1H), 7.41 (s, 1H). ^13^C NMR (DMSO-*d*_6_; δ, ppm): 23.4, 24.4, 27.7, 29.2, 29.9, 32.3, 33.2, 34.6, 40.6, 49.5, 50.5, 50.6, 50.6, 51.5, 55.7, 58.3, 65.1, 65.2, 66.1, 66.2, 66.2, 66.9, 69.7, 70.5, 71.5, 74.1, 76.8, 78.2, 83.9, 92.9, 94.2, 98.8, 123.4, 123.4, 123.4, 123.8, 123.8, 127.2, 127.6, 127.7, 128.3, 136.9, 137.0, 137.2, 137.5, 153.0, 155.4, 155.7. Full ^1^H and ^13^C NMR signal assignments are available in the Supporting Information.

*2,6,4′,6′,4″-(Penta-N-Cbz)-6″-(2-guanidinoethyl-1-)amino)-6″-deoxyapramycin (**7a**)*. To a mixture of 6″-(2-aminoethylamino)-2,6,4′,6′,4″-(penta-*N*-Cbz)-6″-deoxyapramycin (**4a**, 100 mg, 0.08 mmol) and 1*H*-pyrazole-1-carboximidamide hydrochloride (18 mg, 0.16 mmol) in DMF (1 mL) was added diisopropylethylamine (DIPEA, 42 μL, 0.24 mmol). The reaction mixture was stirred at room temperature for 24 h. Completion of the reaction was controlled by TLC. Water (50 mL) was added to the reaction mixture. The forming precipitate was filtered off, washed with H_2_O (3 × 10 mL), and dried under vacuum over P_2_O_5._ The yield of compound **7a** was 70 mg (68%) as a white solid. R_f_ = 0.32 (CHCl_3_:MeOH:HCOOH = 5:1:0.3), HPLC (system A3) *t*_R_ = 9.8 min. HRMS (ESI): calculated for [C_64_H_80_N_9_O_20_]^+^ = 1294.5514, found [M + H]^+^ = 1294.5528. ^1^H NMR (DMSO-*d*_6_; δ, ppm; *J*, Hz): 1.38–3.41 (m, 7H), 1.69–5.19 (m, 9H), 2.52–5.16 (m, 7H), 2.53 (s, 2H), 2.56 (s, 2H), 3.10 (s, 3H), 4.70–5.27 (m, 10H), 7.05 (s, 1H), 7.10 (s, 1H), 7.20–7.40 (m, 25H), 7.21 (s, 1H), 7.23 (s, 1H). ^13^C NMR (DMSO-*d*_6_; δ, ppm): 29.7, 32.2, 34.5, 41.2, 50.1, 50.3, 50.3, 51.2, 52.3, 54.9, 58.0, 65.0, 65.1, 65.2, 66.2, 66.3, 69.5, 69.5, 70.3, 71.6, 72.0, 73.9, 76.9, 82.5, 93.6, 95.2, 97.9, 127.2, 127.5, 127.6, 128.1, 128.2, 137.0, 137.1, 137.2, 137.7, 155.4, 155.5, 155.7, 155.9, 156.3, 161.9.

*2,6,4′,6′,4″-(Penta-N-Cbz)-6″-((3-guanidinopropyl-1)-amino)-6″-deoxyapramycin (**7b**)*. Compound **7b** was prepared from **4b** as described for **7a**. The yield of compound **7b** was 75% as a white solid. R_f_ = 0.30 (CHCl_3_:MeOH:HCOOH = 5:1:0.3), HPLC (system A3) *t*_R_ = 9.2 min. HRMS (ESI): calculated for [C_65_H_82_N_9_O_20_]^+^ = 1308.5671, found [M + H]^+^ = 1308.5710. ^1^H NMR (DMSO-*d*_6_; δ, ppm; *J*, Hz): 1.39–3.41 (m, 7H), 1.69–5.19 (m, 9H), 2.68 (s, 2H), 2.70 (s, 2H), 2.52–5.16 (m, 7H), 2.72 (s, 2H), 3.03 (s, 3H), 4.88–5.24 (m, 10H), 7.08–7.40 (m, 25H), 7.13 (s, 1H), 7.16 (s, 1H), 7.28 (s, 1H), 7.28 (s, 1H). ^13^C NMR (DMSO-*d*_6_; δ, ppm): 29.7, 32.2, 34.5, 39.6, 47.4, 50.1, 50.3, 50.3, 50.7, 51.2, 55.0, 58.0, 65.1, 65.2, 66.2, 66.3, 69.5, 69.5, 70.3, 71.6, 72.0, 73.9, 76.9, 82.5, 93.6, 95.2, 97.9, 127.2, 127.5, 127.6, 128.2, 137.1, 137.2, 137.7, 155.4, 155.7, 155.9, 156.3, 162.3.

*6″-((2-Guanidinoethyl-1)-amino)-6″-deoxyapramycin acetate (**8a**).* Compound **8a** was prepared from **7a** as described for **5a**. The yield of compound **8a** was 76% as a white solid. R_f_ = 0.35 (NH_4_OH:iPrOH 10:7). HRMS (ESI): calculated for [C_24_H_50_N_9_O_10_]^+^ = 624.3675, found [M + H]^+^ = 624.3617. ^1^H NMR (DMSO-*d*_6_; δ, ppm; *J*, Hz): 1.58–3.60 (m, 7H), 1.92 (s, 15H), 1.98–5.51 (m, 9H), 2.63 (s, 3H), 2.76–5.46 (m, 7H), 3.02 (s, 2H), 3.44 (s, 2H). ^13^C NMR (DMSO-*d*_6_; δ, ppm): 26.1, 31.2, 33.8, 33.8, 34.5, 42.3, 49.9, 51.3, 51.6, 52.2, 53.3, 57.2, 63.2, 66.8, 69.2, 72.7, 73.3, 73.4, 74.3, 76.2, 78.2, 86.4, 97.3, 99.7, 159.9, 184.4. Elem. anal.: calculated for [C_24_H_49_N_9_O_10_ × 5AcOH × 1H_2_O]: C, 43.35; H, 7.60; N, 13.38. Found: C, 43.89; H, 7.86; N, 13.07. Full ^1^H and ^13^C NMR signal assignments are available in the Supporting Information.

*6″-((3-Guanidinopropyl-1)-amino)-6″-deoxyapramycin acetate (**8b**).* Compound **8b** was prepared from **7b** as described for **5a**. The yield of compound **8b** was 71% as a white solid. R_f_ = 0.33 (NH_4_OH:iPrOH 10:7). HRMS (ESI): calculated for [C_25_H_52_N_9_O_10_]^+^ = 638.3832, found [M + H]^+^ = 638.3859. ^1^H NMR (DMSO-*d*_6_; δ, ppm; *J*, Hz): 1.57–3.63 (m, 7H), 1.93–5.46 (m, 9H), 1.97 (s, 15H), 2.02 (s, 2H), 2.53 (s, 3H), 2.70–5.49 (m, 7H), 3.36 (s, 2H), 3.10 (s, 2H). ^13^C NMR (DMSO-*d*_6_; δ, ppm): 26.1, 28.3, 32.1, 34.4, 35.0, 41.3, 48.4, 51.4, 51.7, 51.9, 53.3, 57.4, 63.8, 67.6, 69.4, 72.4, 72.9, 73.4, 74.9, 76.8, 78.3, 87.2, 97.3, 98.0, 100.7, 159.7, 184.3. Elem. anal.: calculated for [C_25_H_51_N_9_O_10_ × 5AcOH × 1H_2_O]: C, 43.97; H, 7.70; N, 13.19. Found: C, 44.21; H, 7.94; N, 12.85. Full ^1^H and ^13^C NMR signal assignments are available in the Supporting Information.

### 2.2. Microorganisms and the Minimum Inhibitory Concentration Evaluation

The microbial strains used in this study were obtained from the collection of the Gause Institute of New Antibiotics. The control strains included in the analysis were *S. aureus* ATCC 29213, *E. coli* ATCC 25922, *P. aeruginosa* ATCC 27853, *M. smegmatis* ATCC 607. Clinical isolates *E. coli* MAR17-1350, *K. pneumoniae* APEX-5, *K. pneumoniae* MAR14-3395, and *K. pneumoniae* MAR18-1752 with multidrug resistance, including resistance to aminoglycosides were kindly provided by the Research Institute of Antimicrobial Chemotherapy (RIAC) of the Smolensk State Medical University, Smolensk. The microbial cultures were stored at −75 °C in trypticase soy broth (Becton, Dickinson, France) containing 10–15% glycerol. Storage was carried out in accordance with CLSI guidelines [20]. Prior to experimentation, the bacterial strains were revived from cryopreservation by inoculating them onto trypticase soy agar (Becton, Dickinson, France) and incubating at (36 ± 1) °C for 16–24 h. Individual morphologically homogeneous colonies were then suspended in sterile physiological saline, and the turbidity of the suspension was adjusted to 0.5 McFarland units using a DEN-1 spectrophotometer (Biosan, Riga, Latvia), which corresponds to a concentration of 1.5 × 10^8^ CFU/mL.

#### 2.2.1. Sample Preparation

The samples were dissolved in sterile distilled water, depending on the physicochemical properties of the compounds under investigation, to a concentration of 10,000 µg/mL. Working solutions were then prepared by serial dilution, resulting in concentrations ranging from 64 µg/mL to 0.125 µg/mL.

#### 2.2.2. Assay Setting

Activity was evaluated by determining the minimum inhibitory concentration (MIC) values using the microdilution method in Mueller–Hinton Broth (Beckton and Dickinson, Le Pont-de-Claix, France), in accordance with the standard procedure for assessing the antimicrobial susceptibility of microorganisms [21].

The analysis was conducted in 96-well microtiter plates (Medpolymer, Saint Petersburg, Russia). The bacterial inoculum was introduced within 15 min of preparation. MIC values were determined after incubation for 15–18 h at (36 ± 1) °C. Microbial growth in the presence of the tested compounds was compared to the growth control (without exposure to the test samples). The MIC was defined as the lowest concentration at which visible microbial growth was inhibited.

To ensure the accuracy of the results, the control antibiotics kanamycin A, gentamycin, and tobramycin (Abcr GmbH, Karlsruhe, Germany) were included, along with standard reference strains: *E. coli* ATCC 25922. The MIC values for kanamycin A were determined as follows: *E. coli* ATCC 25922—1–4 µg/mL [22]. For gentamycin, the MIC values were *E. coli* ATCC 25922–0.25–1 µg/mL [23]. For tobramycin, the MIC values were *E. coli* ATCC 25922–0.25–1 µg/mL [23]. The MIC values of the reference strains were required to fall within the acceptable ranges specified in [24], provided that standard assay conditions were maintained.

### 2.3. Determination of the Minimum Inhibitory Concentration Against Aminoglycoside-Resistant Mutants of E. coli

The determination of the MIC values was performed on five strains from the working collection of MSU. *E. coli* JW5503 [25], which lacked a kanamycin resistance cassette that had been removed as per the method outlined in [26], and a strain derived from this strain through *in vitro* selection with kanamycin. The latter strain was found to harbor P610T substitutions in the *fusA* gene. Strains containing AG-modifying enzymes were obtained through the transformation of *E. coli* JW5503 with plasmids that carried cassettes of resistance, namely pCRISPomyces-2–AAC(3)-IV (Addgene #61737), pdualrep2–APH(3′)-II [27] and pt67–ANT(3″) [28]. The MIC determination was performed as described for JW5503 in [19].

### 2.4. Cell-Free Translation Inhibition Assay

The inhibitory effects of the compounds under investigation on in vitro firefly luciferase synthesis were evaluated using the *E. coli* S30 Extract System for Linear Templates (Promega, Madison, WI, USA), as previously described [29]. The working concentration for all tested compounds in the in vitro translation reaction was 0.4 μM. As all compounds were diluted in water, it was used as the negative control.

### 2.5. Determination of Translation Accuracy Using Reporters

To evaluate translation accuracy in the presence of antibiotics, *E. coli* JW5503 (kanamycin resistance cassette removed as per [26]) was transformed with pJC27 plasmids. These plasmids encode the β-galactosidase gene containing a mutation (E537: GAA → GAC/GGG) in its active site, alongside a control plasmid that does not contain this mutation. The synthesis of active β-galactosidase depends on translation errors occurring (when codons GAC and GGG are read as GAA). Petri dishes (9 cm) containing 20 mL of solid LB agar (1.5%), supplemented with chloramphenicol (11.3 μg/mL) and X-Gal (80 μg/mL), were prepared. A secondary soft-agar layer comprising 3.5 mL of LB agar (0.6%) supplemented with 11.3 μg/mL chloramphenicol and 0.5 mL of liquid culture (A600~1.0) was added and allowed to solidify. Antibiotics were applied to the plates in 0.1 μL aliquots at a concentration of 50 mg/mL for tobramycin, streptomycin, kanamycin, and apramycin, and for **5a, 5b, 8a,** and **8b.** The plates were then incubated at 37 °C for 18 h. The presence of blue-indigo staining at the periphery of the inhibition zones, which is a product of X-Gal substrate degradation by β-galactosidase, indicated that the antibiotic had induced translation errors. The results were documented using a Samsung Galaxy Tab A71 camera.

### 2.6. Cytotoxicity Assay

The MTT test [20] was performed in order to evaluate the effect of the compounds on eukaryotic cells’ survivability. Compounds **5a**,**b**, **8a**,**b**, and apramycin, as the reference drug, were tested on HEK293T human embryonic kidney cell line (ATCC). Compounds **5a**,**b**, **8a**,**b**, and apramycin were prepared as water solutions with a 50 mg/mL concentration. The HEK293T cell line was cultivated in DMEM/F-12 medium (Paneco LLC, Moscow, Russia) containing 10% FBS, 50 µg/mL penicillin, and 50 µg/mL streptomycin (Thermo Fisher Scientific, USA) at 37 °C in 5% CO_2_. Triplicates on 96-well plates were used, and 2500 HEK293T cells were seeded in 140 µL of DMEM/F-12 medium in each well during day one. The next day, compounds in twofold successive dilutions were added to these plates (concentrations in wells ranged between 2 and 250 µg/mL for **5a**, **5b**, and **8a**, **8b**). Plates were incubated for 72 h at 37 °C in 5% CO_2_. Afterwards, 0.5 mg/mL of the MTT reagent (Paneco LLC, Moscow, Russia) was added to each well. After 2 h of incubation, the media with MTT was replaced with 140 µL of DMSO, and all plates were placed on a shaker for 10 min for formazan to dissolve. Finally, the absorbance at a 555 nm wavelength was measured on all plates via the plate reader Victor X5 (PerkinElmer, Waltham, MA, USA). The data were then normalized, and dose–response curves and IC_50_ were calculated by means of GraphPad Prism version 8.0.2. (GraphPad Software, Inc., San Diego, CA, USA).

## 3. Results and Discussion

### 3.1. Chemistry

The synthesis of apramycin derivatives was performed in four steps, following a previously established strategy for the modification of the 6″-primary hydroxyl group in 4,6-disubstituted aminoglycosides. This approach has been successfully applied in the preparation of 6″-deoxy derivatives of kanamycin A and tobramycin [18,19]. The primary amino groups of apramycin were selectively protected to prevent undesired side reactions. The second step involved the selective conversion of the 6ʺ-primary hydroxyl group of the 4-amino-4-deoxy-D-glucose residue into a readily cleavable leaving group. This leaving group was substituted with a diamine residue, enabling an additional transformation of the side amino group for the introduction of structural diversity in the apramycin scaffold. Finally, the protecting groups on the amino functionalities were removed, affording the desired apramycin derivatives.

**Scheme 1:**
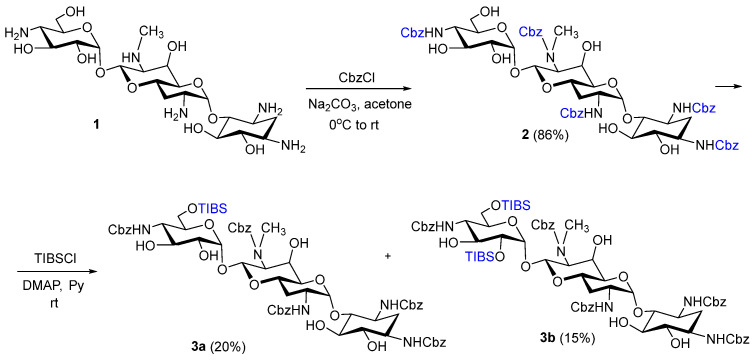
Carbamoylation and further sulfonylation reaction of apramycin (**1**).

The benzyloxycarbonyl (Cbz) group was chosen as the protecting group for the primary amino functionalities of apramycin (**1**). Carbamoylation was carried out in a mixture of acetone and saturated aqueous Na_2_CO_3_, affording the crude protected product. Purification via precipitation from DMSO with water yielded derivative **2** in 86% yield. The key step of the modification sequence involved the selective introduction of an activation group at the 6″-position of apramycin to transform the 6″-hydroxyl into a good leaving group. Following our experience with modification of tobramycin and kanamycin A [18,19], triisopropylsulfonyl chloride (TIBSCl) was employed as a selective reagent for sulfonylation. In contrast to tobramycin and kanamycin A derivatives, sulfonylation of **2** with TIBSCl resulted in a mixture of mono- and di-sulfonylated derivatives, respectively, in almost equal quantities in the crude reaction mixture. This mixture was separated by preparative HPLC to provide the desired mono-sulfonylated product **3a** in 20% yield and the di-sulfonylated by-product **3b** in 15% yield (Scheme 1). The position of the second TIBSO-group in the apramycin derivative **3b** was defined by analysis of NMR spectroscopy data, which clearly showed sulfonylation occurs at the 2″-position of the 4-amino-4-deoxy-D-glucose residue.

**Scheme 2:**
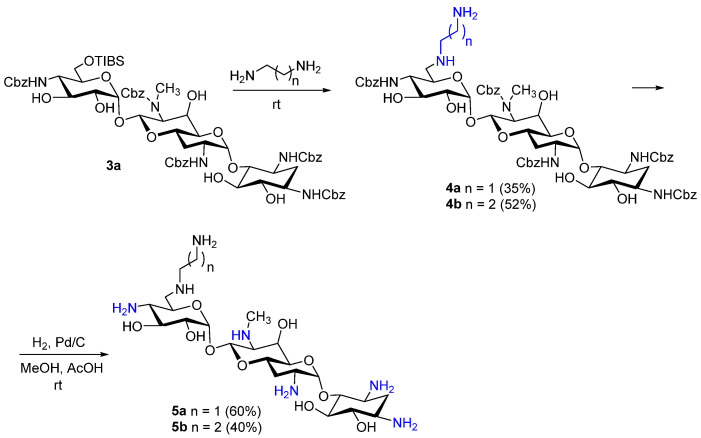
Synthesis of 6″-(aminoalkylamino)-6″-deoxyapramycin derivatives **5a**,**b**.

The 6″-TIBSO group of **3a** was substituted with ethylenediamine and 1,3-diaminopropane, affording derivatives **4a** and **4b** (Scheme 2) in moderate yields (35% and 52%, respectively). Literature reports suggest that the introduction of such pharmacophore moieties enhances both antibacterial activity and aqueous solubility [30]. In the final step, the benzyloxycarbonyl protecting groups were removed via hydrogenolysis on Pd/C in a MeOH–AcOH mixture. This deprotection furnished the target compounds **5a** and **5b** as water-soluble acetate salts, obtained in moderate yields of 60% and 40%, respectively.

**Scheme 3:**
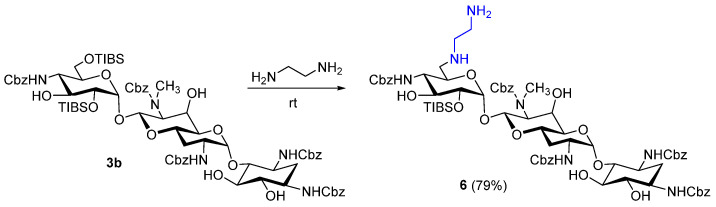
Synthesis of 6″-(aminoethylamino)-2″-TIBSO- apramycin derivative **6**.

Next, we evaluated the possibility of double modification by nucleophilic substitution of two sulfonyloxy groups in **3b** with the above-mentioned diamines. Several attempts to replace both 2″,6″-TIBSO groups in derivative **3b** with ethylenediamine were made. Treatment of **3b** with ethylenediamine at room temperature for 24 h, accompanied by substitution of the primary 6″-TIBSO fragment, led to the derivative **6** with a good yield of 79% (Scheme 3). An increase in the reaction temperature up to 100 °C resulted in slow degradation of the antibiotic core of **6,** while replacement of the secondary and more sterically hindered 2″-TIBSO group under these conditions was not observed.

**Scheme 4:**
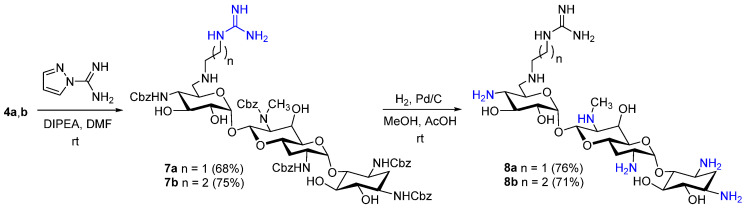
Synthesis of 6″-((guanidinoalkyl)amino)-6″-deoxyapramycin derivatives **8a**,**b**.

Guanidine groups are attractive for potentiating the activity of different classes, including AGs [31], as they have a positive charge at physiological pH, enabling stronger electrostatic and hydrogen-bonding interactions with nucleic acids. In addition, their incorporation can improve bacterial cell penetration and uptake of antibiotics into bacterial cells, enhance their aqueous solubility, and reduce susceptibility to AGEs, thereby strengthening antibacterial efficacy against resistant strains. Notably, streptomycin, one of the earliest AGs, contains a guanidine group, which contributes to its strong interactions with the bacterial ribosome and serves as a precedent for this design strategy. The alkylamino derivatives **4a**,**b** were subsequently subjected to a guanidination reaction with 1*H*-pyrazole-1-carboximidamide, affording compounds **7a** and **7b** in good yields (Scheme 4). Further deprotection of the amino groups in **7a** and **7b** provided the target compounds **8a** and **8b**, isolated as the acetate salts in good yields.

The structures of the synthesized compounds were confirmed and unambiguously assigned based on detailed analysis of their NMR (^1^H, ^13^C, and 2D experiments, Figures S21–S28) and HRMS, all of which were in full agreement with the proposed structures. The composition of the final compounds **5a**,**b**, and **8a**,**b** was also confirmed by elemental analysis data.

### 3.2. In Vitro Antibacterial Activity Studies

To estimate the antibacterial potency of new apramycin derivatives **5a,b**, and **8a,b**, several Gram-negative and Gram-positive bacterial strains were used, including *Staphylococcus aureus* ATCC 29213 (G^+^), *Escherichia coli* ATCC 25922 (G^−^), *Pseudomonas aeruginosa* ATCC 27853 (G^−^), and *Mycobacterium smegmatis* ATCC 607, using apramycin (**1**) as a reference. As summarized in Table 1, introduction of an alkyldiamine substituent at the position 6″ (compounds **5a–b**) resulted in antibacterial activity that was either retained or only slightly reduced compared to the parent compound against *S. aureus* ATCC 29213, *E. coli* ATCC 25922, and *M. smegmatis* ATCC 607. Guanidation of the terminal amine group (derivatives **8a**,**b**) also did not affect the antibacterial efficacy against the tested strains *S. aureus* ATCC 29213, *E. coli* ATCC 25922, and *M. smegmatis* ATCC 607. However, the antibacterial activity of all obtained derivatives **5a**,**b**, **8a**,**b** against *P. aeruginosa* ATCC 27853 strain was 4–8 times weaker than that of apramycin (**1**).

Further evaluation of the antibacterial activity of **5a**,**b**, and **8a**,**b** was conducted towards MDR-positive clinical isolates, including strains resistant to conventional aminoglycosides (Table 2). These experiments were carried out to assess the potential of the novel apramycin derivatives to overcome existing AG resistance mechanisms and expand therapeutic options against multidrug-resistant pathogens. Apramycin and its derivatives demonstrated potent activity, with minimum inhibitory concentrations (MIC) ranging from 1 to 8 μg/mL, whereas tobramycin, gentamicin, and kanamycin A were markedly less effective, displaying MIC values of 32 to >128 μg/mL. Among the synthesized compounds, the alkylamino derivatives **5a**,**b** exhibited moderate activity (4–8 μg/mL), while the corresponding alkylamino-guanidine derivatives **8a**,**b** were more potent, with MICs in the range of 1–4 μg/mL. These results confirmed the prospect of apramycin as the natural scaffold for the development of next-generation AMR-circumventing aminoglycosides.

A comparative evaluation of the new derivatives **5a**,**b**, **8a**,**b**, and paternal apramycin (**1**) was conducted on the *E. coli* strain (JW5503 EF-G P610T) with mutations in the *fusA* gene, which encodes the elongation factor G (Table 3). This mutation has been shown to confer resistance to different AGs [19,32,33]. The parent strain *E. coli* JW5503 kanS exhibited a slightly reduced sensitivity to compounds **5a**,**b**, and **8b** in comparison to apramycin (**1**). However, the MIC value for **8a** did not differ from that of the parent antibiotic. It is noteworthy that derivatives **5a** and **8b** with a shorter side chain at the 6″-position exhibit higher activity in comparison with their analogs that contain three methylene groups, **5b** and **8b**. It is also important to note that guanidinoalkylamine derivatives demonstrated higher activity than derivatives with aminoalkyl substituents. These data are consistent with the results obtained for other pathogens (Table 1 and Table 2). Concurrently, with regard to the resistant strain with a mutation in EF-G, the modified antibiotics demonstrated equivalent activity to the original apramycin. In addition, new compounds were tested on a panel of strains carrying AGEs on plasmids: APH(3′)-II, ANT(3″), and AAC(3)-IV [14]. The new antibiotics **5a**,**b**, **8a**,**b**, like the parent apramycin (**1**), were able to suppress the growth of strains carrying streptomycin- and kanamycin-inactivating proteins (Table 3). In contrast, modification of the 6″-position had no effect on the activity of the apramycin-inactivating enzyme, which was predictable since AAC(3)-IV acetylates the 3-amino position in the deoxystreptamine ring [14].

### 3.3. Mechanism of Action

**Figure 3:**
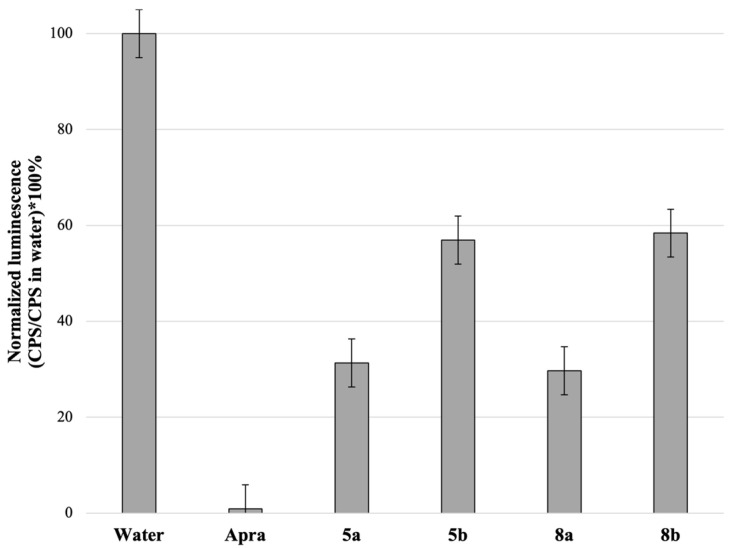
Inhibition of protein synthesis in vitro in the presence of new apramycin derivatives (**5a**, **5b**, and **8a**, **8b**), apramycin, and water (as a control). All compounds were tested at a final concentration of 0.4 μM. The relative maximum accumulation rates of firefly luciferase (Fluc) are shown. The errors correspond to the standard deviation (*n* = 3).

To determine whether the antibacterial activity of the new apramycin derivatives arises from an effect on translation, as in the case of the parent compound [34], or from alternative mechanisms, their action was examined using a cell-free translation system. As shown in Figure 3, all tested derivatives (**5a**, **5b, 8a**, and **8b**) inhibited protein synthesis in vitro, confirming that they retain the ability to interfere with the translational machinery. However, their inhibitory effects at equivalent concentrations were approximately one order of magnitude weaker than that of paternal apramycin (**1**). This finding is particularly interesting, given that the MIC data revealed no corresponding loss of antibacterial potency. One possible explanation is that the modified compounds penetrate into bacterial cells more efficiently or that their efflux is less pronounced, thereby compensating for their reduced intrinsic inhibition of translation. Alternatively, partial metabolic conversion of the derivatives to apramycin in the culture medium or within bacterial cells could account for the observed activity; however, the bioorthogonal nature of the chemical linkage makes this hypothesis unlikely (Figure S37). Beyond their primary action on the ribosome, aminoglycosides also induce alternative cellular effects such as heptosyltransferase I inhibition, membrane-voltage dysregulation, and oxidative stress, which contribute to bacterial killing [35,36,37]. These auxiliary mechanisms may help explain why antibacterial activity can be maintained even when translation inhibition is reduced, as the compound may exploit non-ribosomal processes to exert bactericidal effects.

Notably, derivatives **5a** and **8a**, which contain shorter side chains, exhibited stronger translational inhibition than their analogs bearing longer three-methylene substituents, suggesting that increased steric bulk at the 6″-position may hinder binding to the 16S ribosomal RNA target site. Overall, the observed trends in translation inhibition in vitro are consistent with the antibacterial properties of these compounds (see Table 1, Table 2 and Table 3), since for most strains, the MIC for compounds **5b** and **8b** was greater than the MIC for analogs a. This finding aligns with previous data on similar modifications at position 6″ in tobramycin and kanamycin A derivatives [18,19].

**Figure 4:**
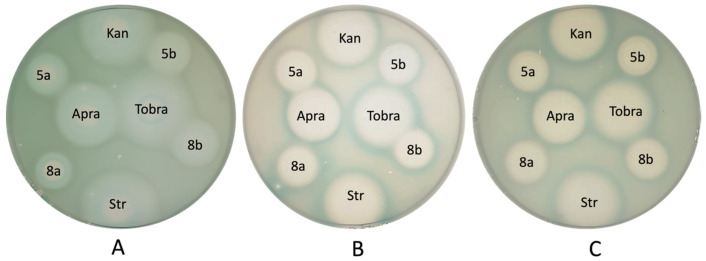
Apramycin and its new derivatives (**5a**,**b**, and **8a**,**b**) increase the misreading of codon 537 of the β-galactosidase reporter gene in *E. coli.* Figure (**A**) shows the construct with the native codon GAA. Figures (**B**,**C**) show the replacement with GGG and GAC, respectively. The test results are shown for compounds **5a**,**b**, **8a**,**b**, as well as for streptomycin (Str), tobramycin (Tobra), kanamycin A (Kan), and apramycin (Apra). The concentration of all antibiotics is 50 mg/mL. The volume of each antibiotic is 0.1 μL.

It is known that apramycin disturbs protein biosynthesis by blocking translocation and inducing translation errors [34]. We tested whether the new aminoglycoside derivatives (**5a**, **5b**, **8a**, and **8b**) retain this ability. This assay uses an *E. coli* strain with a plasmid construct that encodes β-galactosidase in such a way that the functional protein is synthesized only in the presence of translation errors. This is due to the presence of the E537D or E537G substitution in the enzyme’s active site. Correct translation results in the synthesis of non-functional β-galactosidase, which is incapable of cleaving X-gal. However, only if there is an error is the functional enzyme produced, resulting in an indigo stain along the edge of the inhibition zone. Consequently, if the antibiotic causes translation errors, blue staining is observed at the edge of the inhibition zone. As can be seen in Figure 4, the compounds **5a**, **5b**, **8a**, and **8b** caused blue staining of the bacterial lawn in the same way as the parent antibiotic **1**, indicating that they also cause translation errors in the target cells. In this regard, introducing aminoalkylamino or guanidinoalkylamino residues at the 6″-position of apramycin preserves the original antibacterial mechanism of aminoglycosides, affecting the fidelity of protein synthesis. These observations also confirm our previous findings that 6″-derivatives of kanamycin and tobramycin retain the ability to induce translation errors [18,19].

### 3.4. Cytotoxicity Assessment

**Figure 5:**
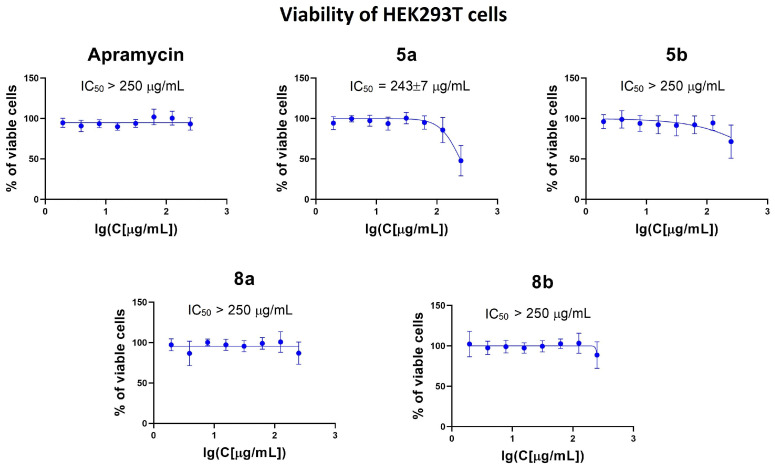
Viability curves (MTT test, 72 h) of HEK293T cell line treated with apramycin, **5a**, **5b**, and **8a**, **8b**.

The new apramycin derivatives **5a**,**b**, and **8a**,**b** exhibited antibacterial activity comparable to that of the parent compound **1**, prompting an evaluation of their cytotoxicity to mammalian cells. Cytotoxic effects were assessed using the standard MTT assay [20] on human embryonic kidney (HEK293T) cells to determine potential off-target toxicity. As shown in Figure 5, derivatives **5b**, **8a**, **8b**, and apramycin itself demonstrated no measurable cytotoxicity up to the highest tested concentration (IC_50_ > 250 µg/mL), indicating a favorable safety profile. Only compound **5a** exhibited a detectable but still low level of cytotoxicity, with an IC_50_ value of 243 ± 7 µg/mL. These results indicate that chemical modification at the 6″-position of apramycin does not significantly increase its toxicity toward eukaryotic cells. The maintenance of low cytotoxicity across all tested derivatives supports the conclusion that the introduced structural changes do not adversely affect the compound’s selectivity for bacterial targets.

## 4. Conclusions

In this study, we synthesized and structurally confirmed a series of novel apramycin derivatives modified at the 6″-position of the 4-amino-4-deoxy-D-glucose residue. A key finding was the distinct behavior of *N*-protected apramycin derivatives in the sulfonylation reaction: unlike tobramycin and kanamycin A, which undergo selective modification exclusively at the 6″-hydroxyl, apramycin was functionalized at both the 6″- and 2″-positions, yielding comparable amounts of mono- and di-sulfonylated products. Subsequent introduction of aminoalkylamino and guanidinoalkylamino substituents generated derivatives that preserved antibacterial activity and retained the ribosomal mechanism of action. Among these, guanidine-containing analogs **8a** and **8b** exhibited enhanced potency against multidrug-resistant G^−^ clinical isolates relative to their aminoalkylamino counterparts, confirming the guanidino group as a valuable pharmacophore. Importantly, all derivatives as well as paternal apramycin demonstrated low cytotoxicity in mammalian cells, supporting their therapeutic potential.

Despite these promising findings, the modifications did not achieve substantial or consistent improvements over parent apramycin, particularly against the apramycin-resistant *E. coli* strain. Nevertheless, apramycin’s favorable activity profile and its ability to evade common AG resistance mechanisms underscore its value as a privileged scaffold for next-in-class drug design. Future research should prioritize systematic structure–activity relationship studies, advanced chemical optimization, and evaluation in relevant infection models to unlock the full potential of apramycin-based antibiotics in addressing the global challenge of antimicrobial resistance.

## Figures and Tables

**Table 1:** Antibacterial potency (MIC, µg/mL) of the new apramycin derivatives **5a**,**b**, **8a**,**b**, and the reference drug apramycin.

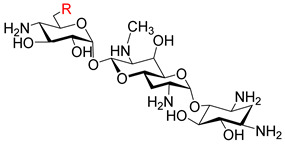					
Compound	R	MIC ^a^, µg/mL			
		*S. aureus* ATCC 29213	*E. coli* ATCC 25922	*P. aeruginosa* ATCC 27853	*M. smegmatis* ATCC 607
**5a**	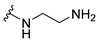	2	2	8	0.25
**5b**	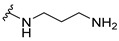	2	2	16	0.25
**8a**	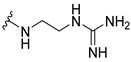	0.5	1	8	0.125
**8b**	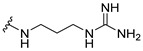	0.5	1	8	0.125
Apramycin (**1**)	OH	1	1	2	0.125

^a^ MIC refers to the minimum inhibitory concentration, defined as the lowest concentration of a compound capable of suppressing visible bacterial growth. All MIC values reported in this study were confirmed through three independent experimental repetitions, ensuring reproducibility of the results.

**Table 2:** Antibacterial potency (MIC, µg/mL) of new apramycin derivatives **5a**,**b**, **8a**,**b**, and reference AGs against MDR clinical isolates.

	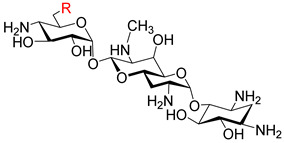					
Compound	R	MIC, µg/mL				
		*E. coli* ATCC 25922	*E. coli* MAR17-1350 *^a^*	*K. pneumoniae* APEX-5 *^b^*	*K. pneumoniae* MAR14-3395 *^c^*	*K. pneumoniae* MAR18-1752 *^d^*
**5a**	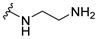	2	4	4	4	4
**5b**	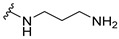	2	8	8	8	8
**8a**	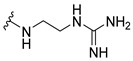	1	4	1	2	2
**8b**	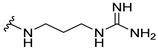	1	4	2	4	2
Apramycin (**1**)	OH	1	4	1	1	1
Tobramycin	-	0.5	>128	32	>128	>128
Gentamycin	-	0.25	>128	32	>128	>128
Kanamycin A	-	2	>128	>128	>128	>128

***Genetic determinants of resistance: ^a^*** NDM-5(blaTEM-1 + blaNDM-5 + aadA2 + rmtB1 + mph(A) + dfrA12 + sul1 + qacEdelta1 + ble-MBL + ftsI_N337NYRIN); **^*b*^** NDM-16(blaOXA-1 + blaSHV-11 + blaTEM-1 + blaNDM-16 + aac(6′)-Ib-cr + aac(3)-IIe + aph(3′)-VIa(mut) + aph(3ʺ)-Ib(mut) + aph(6)Id + oqxA + oqxB + fosA(mut) + catA1 + catA2 + catB3(trancated) + tet(A) + tet(D) + dfrA14 + sul2 + ble-MBL); **^*c*^** NDM-1 + OXA-244(OXA-48-rp.)(blaOXA-1 + blaSHV-11 + blaTEM-1 + blaNDM-1 + blaOXA-244 + aac(6′)-Ib-cr(trancated) + aph(3′)-VI + aadA2 + armA + oqxA + oqxB + qnrS1 + fosA + mph(E) + msr(E) + catA1 + catB3(trancated) + tet(A) + dfrA1 + dfrA12 + sul1 + qacEdelta1 + ble-MBL); **^*d*^** NDM-1(blaOXA-1 + blaSHV-11 + blaTEM-1 + blaNDM-1 + aac(6′)-Ib-cr + aph(3′)-VI + armA + oqxA + oqxB + qnrS1 + fosA + mph(A) + mph(E) + msr(E) + catA1 + catB3(trancated) + tet(A) + dfrA1 + dfrA5 + sul1 + sul2 + qacEdelta1 + ble-MBL).

**Table 3:** The antibacterial activity (MIC, µg/mL) of new apramycin derivatives **5a**,**b**, **8a**,**b**, and reference AGs against resistant *E. coli* JW5503 EF-G mutant P610T and transformants, carrying AG-modifying enzymes on plasmids (KanR–APH(3′)-II, SmR–ANT(3″), ApmR–AAC(3)-IV), resistant to kanamycin A, streptomycin, and apramycin, respectively.

		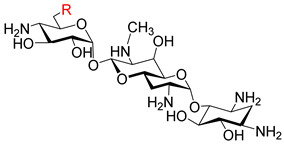				
Compound	R	MIC, µg/mL				
		*E. coli* JW5503 kanS	*E. coli* JW5503 EF-G P610T	*E. coli* JW5503 KanR	*E. coli* JW5503 SmR	*E. coli* JW5503 ApmR
**5a**	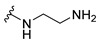	16	32	16	32	>256
**5b**	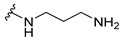	32	64	32	32	>256
**8a**	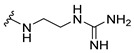	8	32	8	16	>256
**8b**	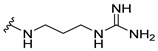	16	64	16	32	>256
**Apramycin (1)**	OH	8	64	4	8	>256
**Tobramycin**	-	2	16	2	4	>256
**Streptomycin**	-	4	8	4	>256	4
**Kanamycin A**	-	4	64	>256	8	32

## Data Availability

Data are contained within the article and in the Supporting Information file.

## Supplementary Materials

The following supporting information can be downloaded at: https://www.mdpi.com/article/10.3390/pharmaceutics17121583/s1, Tables S1–S3: Assignments of the signals in ^1^H and ^13^C NMR spectra of apramycin derivatives; Figures S1–S36: ^1^H, ^13^C, COSY, and HSQC NMR spectra of the apramycin derivatives; Figure S37: 1H NMR spectrum of compound **5a**. The top spectrum was recorded 3 months after the bottom one.

## Abbreviations

The following abbreviations are used in this manuscript:

AG	Aminoglycoside
AME	aminoglycoside-modifying enzyme
AMR	antimicrobial resistance
Cbz	Benzyloxycarbonyl
DMSO	Dimethylsulfoxide
IC_50_	the amount of a drug that causes the inhibition of the growth of 50% of cells
HPLC	high-performance liquid chromatography
HRMS (ESI)	high-resolution mass spectrometry with electrospray ionization
MDR	multidrug resistance
MIC	minimum inhibitory concentration
NMR	nuclear magnetic resonance
TIBS	2,4,6-triisopropylbenzenesulfonyl
TLC	thin layer chromatography
WHO	World Health Organization
