# Supplementary material for: Design, Synthesis, and Biological Evaluation of 6″-Modified Apramycin Derivatives to Overcome Aminoglycoside Resistance
Source: Pharmaceutics. 2025 Dec 8;17(12):1583. doi: 10.3390/pharmaceutics17121583 (PMC12736951; doi:10.3390/pharmaceutics17121583)
Supplement: Supplementary file 1 [file pharmaceutics-17-01583-s001.zip › pharmaceutics-3990379-supplementary.pdf]

SUPPORTING INFORMATION

Design, Synthesis, and Biological Evaluation of 6″-Modified Apramycin Derivatives to Overcome Aminoglycoside Resistance

Kseniya S. Shapovalova [1], Georgy V. Zatonsky [1], Elizaveta A. Razumova [2,3], Nikolai D. Dagaev [3,4], Dmitrii A. Lukianov [2,5], Natalia E. Grammatikova [1], Alexander S. Tikhomirov [1], Andrey E. Shchekotikhin [1,*]

[1] Gause Institute of New Antibiotics, 11 B. Pirogovskaya Street, Moscow 119021, Russian Federation;
[2] Department of Chemistry, Lomonosov Moscow State University, Leninskie Gory 1, 119991 Moscow, Russia;
[3] A.N. Belozersky Institute of Physico-Chemical Biology, Lomonosov Moscow State University, 119991, Moscow, Russia;
[4] Faculty of Bioengineering and Bioinformatics, Lomonosov Moscow State University, 119991 Moscow, Russia
[5] Center for Molecular and Cellular Biology, Moscow, Skolkovo, 121205, Russia;
* Correspondence: shchekotikhin@mail.ru

Table of content

Table S1. ^{1}H and ^{13}C NMR data of apramycin derivatives **2**, **3a, 4a-b** in DMSO-d_6. 4

Table S2. ^{1}H and ^{13}C NMR data of apramycin derivatives **3b**, **6,** in DMSO-d_6. 5

Table S3. ^{1}H and ^{13}C NMR data of apramycin derivatives **5a-b**, **8a-b** in D_2O. 7

Figure S1. ^{1}H NMR (500.2 MHz, DMSO-d_6) spectrum of 4,6,2′,6′,4″-penta-*N*-Cbz-apramycin **2** 8

Figure S2. ^{13}C NMR (125.8 MHz, DMSO-d_6) spectrum of 4,6,2′,6′,4″-penta-*N*-Cbz-apramycin **2** 9

Figure S3. ^{1}H NMR (500.2 MHz, DMSO-d_6) spectrum of 4,6,2′,6′,4″-penta-*N*-Cbz-6″-*O*-(2,4,6-triisopropylbenzosulfonyl)apramycin **3**a 10

Figure S4. ^{13}C NMR (125.8 MHz, DMSO-d_6) spectrum of of 4,6,2′,6′,4″-penta-*N*-Cbz-6″-*O*-(2,4,6-triisopropylbenzosulfonyl)apramycin **3a** 11

Figure S5. ^{1}H NMR (500.2 MHz, DMSO-d_6) spectrum of 6″-(2-aminoethyamino)-4,6,2′,6′,4″-penta-*N*-Cbz-6″-deoxyapramycin **4a** 12

Figure S6. ^{13}C NMR (125.8 MHz, DMSO-d_6) spectrum of 6″-(2-aminoethyamino)-4,6,2′,6′,4″-penta-*N*-Cbz-6″-deoxyapramycin **4a** 13

Figure S7. ^{1}H NMR (500.2 MHz, DMSO-d_6) spectrum of 6″-(3-aminopropyl-1-amino)-4,6,2′,6′,4″-penta-*N*-Cbz-6″-deoxyapramycin **4b** 14

Figure S8. ^{13}C NMR (125.8 MHz, DMSO-d_6) spectrum of 6″-(3-aminopropyl-1-amino)-4,6,2′,6′,4″-penta-*N*-Cbz-6″-deoxyapramycin **4b** 15

Figure S9. ^{1}H NMR (500.2 MHz, DMSO-d_6) spectrum of 4,6,2′,6′,4″-penta-*N*-Cbz-2″,6″-(di-*O*-(2,4,6-triisopropylbenzosulfonyl))apramycin **3b** 16

Figure S10. ^{13}C NMR (125.8 MHz, DMSO-d_6) spectrum of 4,6,2′,6′,4″-penta-*N*-Cbz-2″,6″-(di-*O*-(2,4,6-triisopropylbenzosulfonyl))apramycin **3b** 17

Figure S11. ^{1}H NMR (500.2 MHz, DMSO-d_6) spectrum of 4,6,2′,6′,4″-penta-*N*-Cbz-2″-*O*-(2,4,6-triisopropylbenzosulfonyl)-6″-(2-aminoethyamino)-6″-deoxyapramycin **6** 18

Figure S12. ^{13}C NMR (125.8 MHz, DMSO-d_6) spectrum of 4,6,2′,6′,4″-penta-*N*-Cbz-2″-*O*-(2,4,6-triisopropylbenzosulfonyl)-6″-(2-aminoethyamino)-6″-deoxyapramycin **6** 19

Figure S13. ^{1}H NMR (500.2 MHz, DMSO-d_6) spectrum of 4,6,2′,6′,4″-penta-*N*-Cbz-6″-(2-guanidinoethylamino)-6″-deoxyapramycin **7a** 20

Figure S14. ^{13}C NMR (125.8 MHz, DMSO-d_6) spectrum of 4,6,2′,6′,4″-penta-*N*-Cbz-6″-(2-guanidinoethylamino)-6″-deoxyapramycin **7a** 21

Figure S15. COSY NMR (DMSO-d_6) spectrum of spectrum of 4,6,2′,6′,4″-penta-*N*-Cbz-6″-(2-guanidinoethylamino)-6″-deoxyapramycin **7a** 22

Figure S16. HSQC NMR (DMSO-d_6) spectrum of 4,6,2′,6′,4″-penta-*N*-Cbz-6″-(2-guanidinoethylamino)-6″-deoxyapramycin **7a** 23
Figure S17. ^{1}H NMR (500.2 MHz, DMSO-d_6) spectrum of 4,6,2′,6′,4″-(penta-N-Cbz)-6″-(3-guanidinpropil-1-amino)-6″-deoxyapramycin **7b** 24
Figure S18. ^{13}C NMR (125.8 MHz, DMSO-d_6) spectrum of 4,6,2′,6′,4″-penta-*N*-Cbz-6″-(3-guanidinpropil-1-amino)-6″-deoxyapramycin **7b** 25
Figure S19. COSY NMR (DMSO-d_6) spectrum of spectrum of 4,6,2′,6′,4″-penta-*N*-Cbz-6″-(3-guanidinpropil-1-amino)-6″-deoxyapramycin **7b** 26
Figure S20. HSQC NMR (DMSO-d_6) spectrum of 4,6,2′,6′,4″-penta-*N*-Cbz-6″-(3-guanidinpropil-1-amino)-6″-deoxyapramycin **7b** 27
Figure S21. ^{1}H NMR (500.2 MHz, D_2O) spectrum of 6″-(2-aminoethyamino)-6″-deoxyapramycin **5a** 28
Figure S22. ^{13}C NMR (125.8 MHz, D_2O) spectrum of 6″-(2-aminoethyamino)-6″-deoxyapramycin **5a** 29
Figure S23. COSY NMR (D_2O) spectrum of spectrum of 6″-(2-aminoethyamino)-6″-deoxyapramycin **5a** 30
Figure S24. HSQC NMR (D_2O) spectrum of 6″-(2-aminoethyamino)-6″-deoxyapramycin **5a** 31
Figure S25. ^{1}H NMR (500.2 MHz, D_2O) spectrum of 6″-(3-aminopropyl-1-amino)-6″-deoxyapramycin **5b**32
Figure S26. ^{13}C NMR (125.8 MHz, D_2O) spectrum of 6″-(3-aminopropyl-1-amino)-6″-deoxyapramycin **5b**33
Figure S27. COSY NMR (D_2O) spectrum of 6″-(3-aminopropyl-1-amino)-6″-deoxyapramycin **5b** 34
Figure S28. HSQC NMR (D_2O) spectrum of 6″-(3-aminopropyl-1-amino)-6″-deoxyapramycin **5b**........ 35
Figure S29. 1H NMR (500.2 MHz, D2O) spectrum of 6″-(2-guanidinoethylamino)-6″-deoxyapramycin **8a**36
Figure S30. ^{13}C NMR (125.8 MHz, D_2O) spectrum of 6″-(2-guanidinoethylamino)-6″-deoxyapramycin **8a**37
Figure S31. COSY NMR (D_2O) spectrum of spectrum of 6″-(2-guanidinoethylamino)-6″-deoxyapramycin **8a**........ 38
Figure S32. HSQC NMR (D_2O) spectrum of 6″-(2-guanidinoethylamino)-6″-deoxyapramycin **8a**........ 39
Figure S33. ^{1}H NMR (500.2 MHz, D_2O) spectrum of 6″-(3-guanidinpropil-1-amino)-6″-deoxyapramycin **8b**40
Figure S34. ^{13}C NMR (125.8 MHz, D_2O) spectrum of 6″-(3-guanidinpropil-1-amino)-6″-deoxyapramycin **8b** 41
Figure S35. COSY NMR (D_2O) spectrum of spectrum of 6″-(3-guanidinpropil-1-amino)-6″-deoxyapramycin **8b** 42
Figure S36. HSQC NMR (D_2O) spectrum of 6″-(3-guanidinpropil-1-amino)-6″-deoxyapramycin **8b**........ 43
Figure S37. ^{1}H NMR spectrum of compound **5a** (SK-223). The top spectrum was recorded 3 months after the bottom one. 44

Table S1. ^{1}H and ^{13}C NMR data of apramycin derivatives **2**, **3a, 4a-b** in DMSO-d_6.

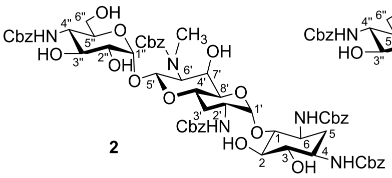

2

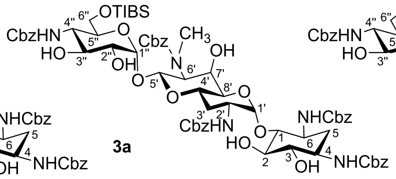

3a

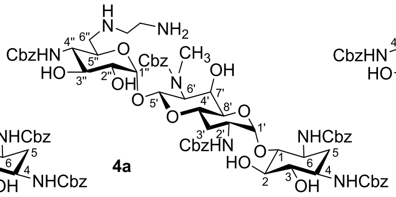

4a

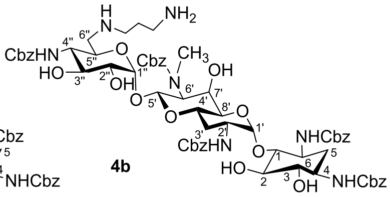

4b

	Chemical shift, ppm							
Compound	**2**		**3a**		**4a**		**4b**	
Position of C atom	^{1}H NMR	^{13}C NMR	^{1}H NMR	^{13}C NMR	^{1}H NMR	^{13}C NMR	^{1}H NMR	^{13}C NMR
1	3.39	82.5	3.44	82.7	3.39	82.5	3.39	82.5
2	3.30	76.9	3.33	76.9	3.29	76.9	3.29	76.9
3	3.11	73.9	3.17	74.2	3.10	73.9	3.10	73.9
4	3.26	51.2	3.29	51.5	3.26	51.2	3.26	51.2
5	1.77/1.39	34.5	1.88/1.41	34.5	1.77/1.38	34.5	1.78/1.39	34.5
6	3.41	50.1	3.44	50.4	3.41	50.1	3.41	50.1
1’	5.06	97.9	5.09	98.0	5.05	97.9	5.05	97.9
2’	3.56	50.3	3.60	50.4	3.55	50.3	3.55	50.3
3’	1.97/1.70	29.7	2.01/1.70	29.9	1.97/1.69	29.7	1.98/1.69	29.7
4’	3.72	66.2	3.74	66.4	3.73	66.3	3.73	66.3
5’	3.54	70.3	3.57	70.3	3.53	70.3	3.53	70.3
6’	4.16	69.5	4.18	69.6	4.15	69.5	4.15	69.5
7’	3.99	58.0	4.01	58.3	3.99	58.0	3.99	58.0
8’	5.21	93.4	5.13	95.6	5.19	93.6	5.19	93.6
1”	5.17	95.2	5.48	95.0	5.16	95.2	5.16	95.2
2”	3.31	72.0	4.51	76.7	3.29	72.0	3.29	72.0
3”	3.52	69.4	3.58	67.9	3.47	69.5	3.47	69.5
4”	3.18	53.6	3.58	57.2	3.22	54.9	3.19	55.0
5”	3.51	72.8	3.86	70.0	3.59	71.6	3.59	71.6
6”	3.42/3.34	61.1	4.11/3.89	69.1	2.52	50.3	2.52	50.3
2’-N	7.28	-	7.28	-	7.27	-	7.26	-
6’-N-**<u>CH3</u>**	3.00	32.2	3.00	31.9	3.03	32.2	3.03	32.2
4-N	7.15	-	6.83	-	7.13	-	7.14	-
6-N	7.10	-	7.14	-	7.09	-	7.10	-
4”-N	7.32	-	7.42	-	7.25	-	7.26	-
Ar (Cbz)	7.21-7.41	128.7/128.5/128.2/127.6/127.2	7.11-7.41	128.6/128.4/128.3/128.1/127.7/127.5/127.2/123.7/123.5	7.20-7.40	128.2/128.1/127.6/127.5/127.2	7.08-7.40	128.2/127.6/127.5/127.2
CH_2 (Cbz)	4.97-5.23	66.2/65.3/65.1	4.78-5.27	66.2/66.1/65.4/65.1	4.70-5.27	66.2/65.2/65.1/65.0	4.88-5.24	66.2/65.2/65.1
C=O (Cbz)	-	156.5/155.9/155.9/155.7/155.4	-	156.3/155.1/155.8/155.7/155.4/153.8	-	156.3/155.9/155.7/155.5/155.4	-	156.3/155.9/155.7/155.4
Cq	-	137.7/137.2/137.1/136.9		137.2/137.1/137.0/136.9/136.8		137.7/137.2/137.1/137.0		137.7/137.2/137.1
Substituent 6”			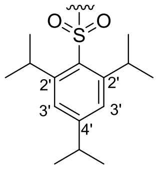		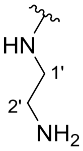		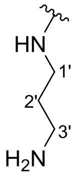	
1’			-	123.4	2.44	52.3	2.52	50.7
2’			-	123.4	2.49	41.2	2.55	39.6
3’			6.95	123.8			2.46	47.4
4’			-	123.8				
2’-CH			2.79	27.9				
2’-CH_3			1.16	23.8/24.8				
4’-CH			4.57	33.2				
4’-CH_3			1.10	34.4				

Table S2. ^{1}H and ^{13}C NMR data of apramycin derivatives **3b**, **6,** in DMSO-d_6.

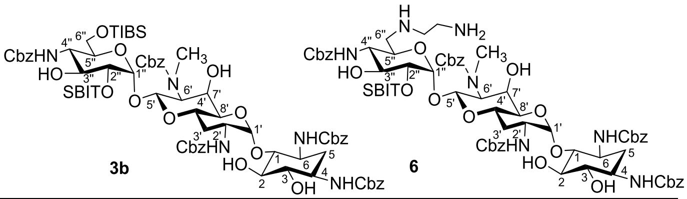

Compound	3b		6	
Position of C atom	^{1}H NMR	^{13}C NMR	^{1}H NMR	^{13}C NMR
1	3.42	83.7	3.43	83.9
2	3.31	76.8	3.32	76.8
3	3.17	73.9	3.19	74.1
4	3.29	51.2	3.30	51.5
5	1.91/1.37	34.7	1.94/1.39	34.6
6	3.42	50.5	3.43	50.6
1’	4.95	98.8	4.96	98.8
2’	3.57	50.5	3.58	50.5
3’	1.92/1.58	29.6	1.93/1.59	29.9
4’	3.67	66.2	3.71	66.2
5’	3.51	70.4	3.52	70.5
6’	4.17	69.5	4.19	69.7
7’	3.95	58.1	3.95	58.3
8’	5.14	93.8	5.18	94.2
1”	5.40	93.2	5.44	92.9
2”	3.34	77.5	4.45	78.2
3”	3.80	65.9	3.77	66.9
4”	3.30	54.4	3.45	55.7
5”	3.93	69.6	3.69	71.5
6”	4.11/3.92	68.6	2.63/2.58	49.5
2’-N	7.16	-	7.34	-
6’-N-$\underline{\mathbf{CH_3}}$	2.98	31.8	3.04	32.3
4-N	6.92	-	7.16	-
6-N	6.69	-	6.95	-
4”-N	7.28	-	7.41	-
Ar (Cbz)	7.21-7.38	128.3/127.7/127.2/123.9/123.5	7.22-7.36	128.3/127.7/127.6/127.2/123.4
CH_2 (Cbz)	4.94-5.15	66.2/66.1/65.4/65.1	4.96-5.08	66.2/66.1/65.2/65.1
C=O (Cbz)	-	155.8/155.4/153.9/153.1/150.4/149.9	-	155.7/155.4/153.0
Cq	-	137.5/137.2/136.8	-	137.5/137.2/137.0/136.9
Substituent 6”	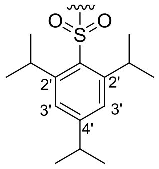		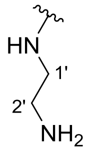	
1’	-	123.4	2.56	50.6
2’	-	123.4	2.62	40.6
3’	7.21	123.8		
4’	-	123.8		
2’-CH	4.02-4.12	29.1		
2’-CH_3	1.16-1.24	23.2		
4’-CH	4.02-4.12	29.1		
4’-CH_3	1.16-1.24	34.4		
Substituent 2”	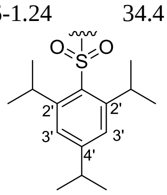		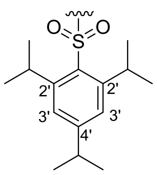	
1’	-	123.4	-	123.4

2’	-	123.4	-	123.4
3’	7.32	123.8	7.30	123.8
4’	-	123.8	-	123.8
2’-CH	4.02-4.12	29.1	2.93	29.2
2’-CH_3	1.16-1.24	23.2	1.12-1.27	23.2/24.4
4’-CH	4.02-4.12	29.1	4.13	33.2
4’-CH_3	1.16-1.24	34.4	1.12-1.27	27.7

Table S3. ^{1}H and ^{13}C NMR data of apramycin derivatives **5a-b**, **8a-b** in D$_2$O.

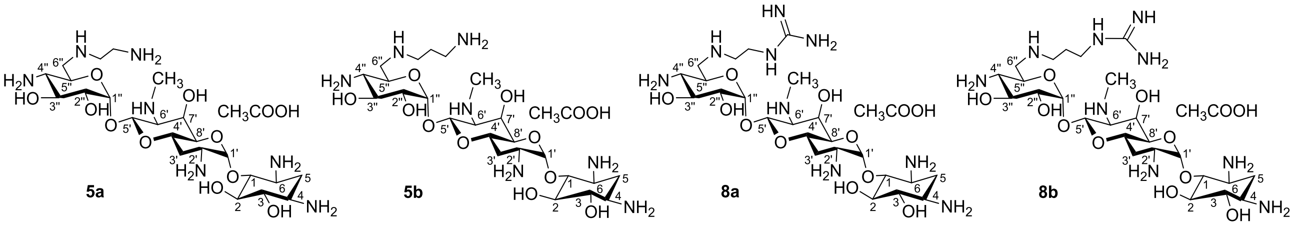

Chemical shift, ppm								
Compound	**5a**		**5b**		**8a**		**8b**	
Position of C atom	^{1}H NMR	^{13}C NMR	^{1}H NMR	^{13}C NMR	^{1}H NMR	^{13}C NMR	^{1}H NMR	^{13}C NMR
1	3.61	86.2	3.61	86.1	3.55	86.4	3.53	87.2
2	3.65	78.2	3.64	78.2	3.60	78.2	3.63	78.3
3	3.55	76.3	3.55	76.3	3.49	76.2	3.50	76.8
4	3.27	53.2	3.27	53.2	3.21	53.3	3.20	53.3
5	2.34/1.94	34.3	2.33/1.64	34.2	2.28/1.58	34.5	2.28/1.57	35.0
6	3.18	51.6	3.18	51.6	3.12	51.6	3.10	51.7
1’	5.57	99.5	5.58	99.4	5.51	99.7	5.46	100.7
2’	3.59	51.2	3.61	51.2	3.53	51.3	3.43	51.4
3’	2.39/2.03	30.9	2.39/2.02	30.8	2.33/1.98	31.2	2.32/1.93	32.1
4’	3.96	69.2	3.95	69.1	3.90	69.2	3.90	69.4
5’	3.87	72.7	3.87	72.8	3.82	72.7	3.83	72.9
6’	4.52	66.6	4.52	66.5	4.46	66.8	4.43	67.6
7’	3.20	63.1	3.14	63.2	3.12	63.2	2.92	63.8
8’	5.17	96.7	5.12	97.3	5.10	33.8	5.04	98.0
1”	5.51	97.3	5.46	97.3	5.46	97.3	5.49	97.3
2”	3.72	73.4	3.73	73.4	3.68	73.4	3.69	73.4
3”	3.75	74.1	3.71	74.5	3.70	74.3	3.68	74.9
4”	2.88	56.9	2.75	57.3	2.76	57.2	2.70	57.4
5”	3.84	73.8	3.81	73.1	3.83	73.3	3.90	72.4
6”	3.05/2.95	52.2	3.39/3.21	51.9	3.12/2.98	52.2	3.40/3.18	51.9
2’-N	-	-	-	-	-	-	-	-
6’-N-**<u>CH$_3$</u>**	2.71	33.6	2.71	33.7-	2.63	33.8	2.53	34.4
4-N	-	-	-	-	-	-	-	-
6-N	-	-	-	-	-	-	-	-
4”-N	-	-	-	-	-	-	-	-
Substituent 6”	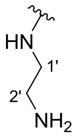		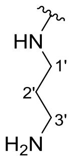		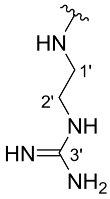		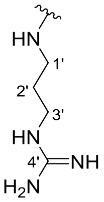	
1’	3.03	48.7	3.13	48.1	3.02	49.9	3.10	48.4
2’	3.20	41.0	2.11	27.1	3.44	42.3	2.02	28.3
3’			3.10	40.9	-	159.9	3.36	41.3
4’							-	159.7
CH$_3$ (Acetate)	1,97	26.2	1.97	26.1	1.92	26.1	1.97	26.1
COOH (Acetate)	-	184.2	-	184.3	-	184.4	-	184.3

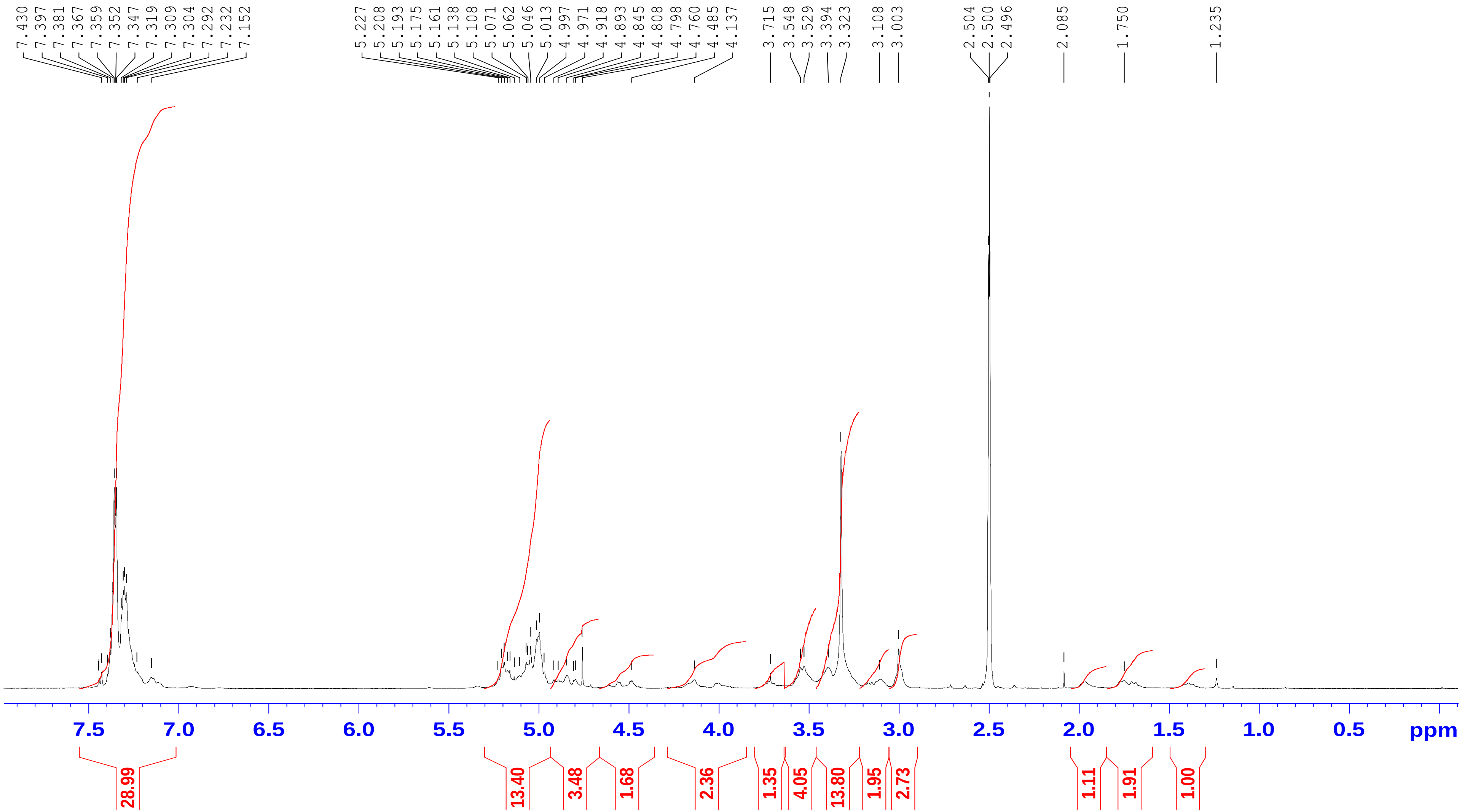

Figure S1. ^{1}H NMR (500.2 MHz, DMSO-d_6) spectrum of 4,6,2′,6′,4″-penta-*N*-Cbz-apramycin **2**

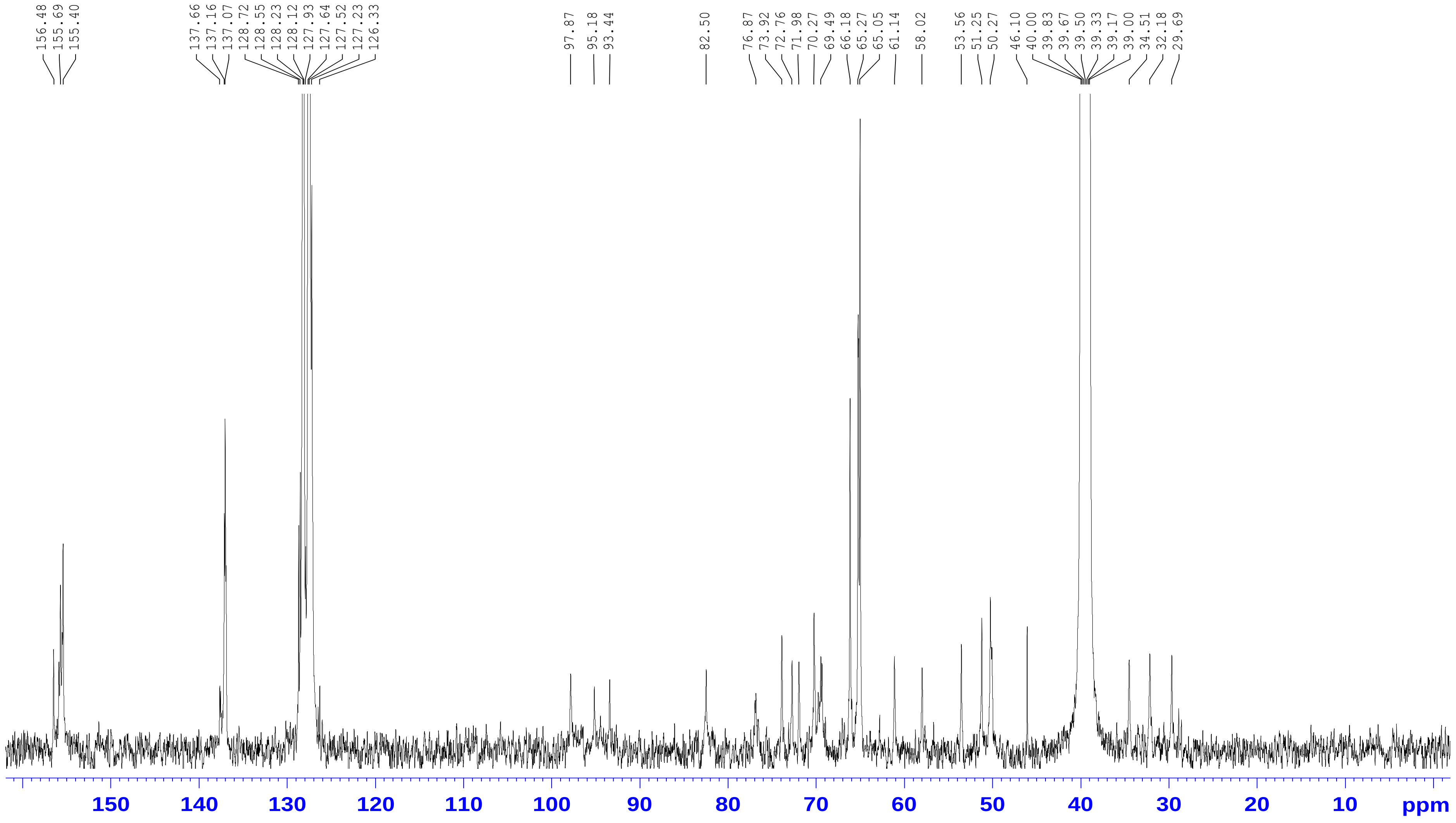

Figure S2. ^{13}C NMR (125.8 MHz, DMSO-d_6) spectrum of 4,6,2′,6′,4″-penta-*N*-Cbz-apramycin **2**

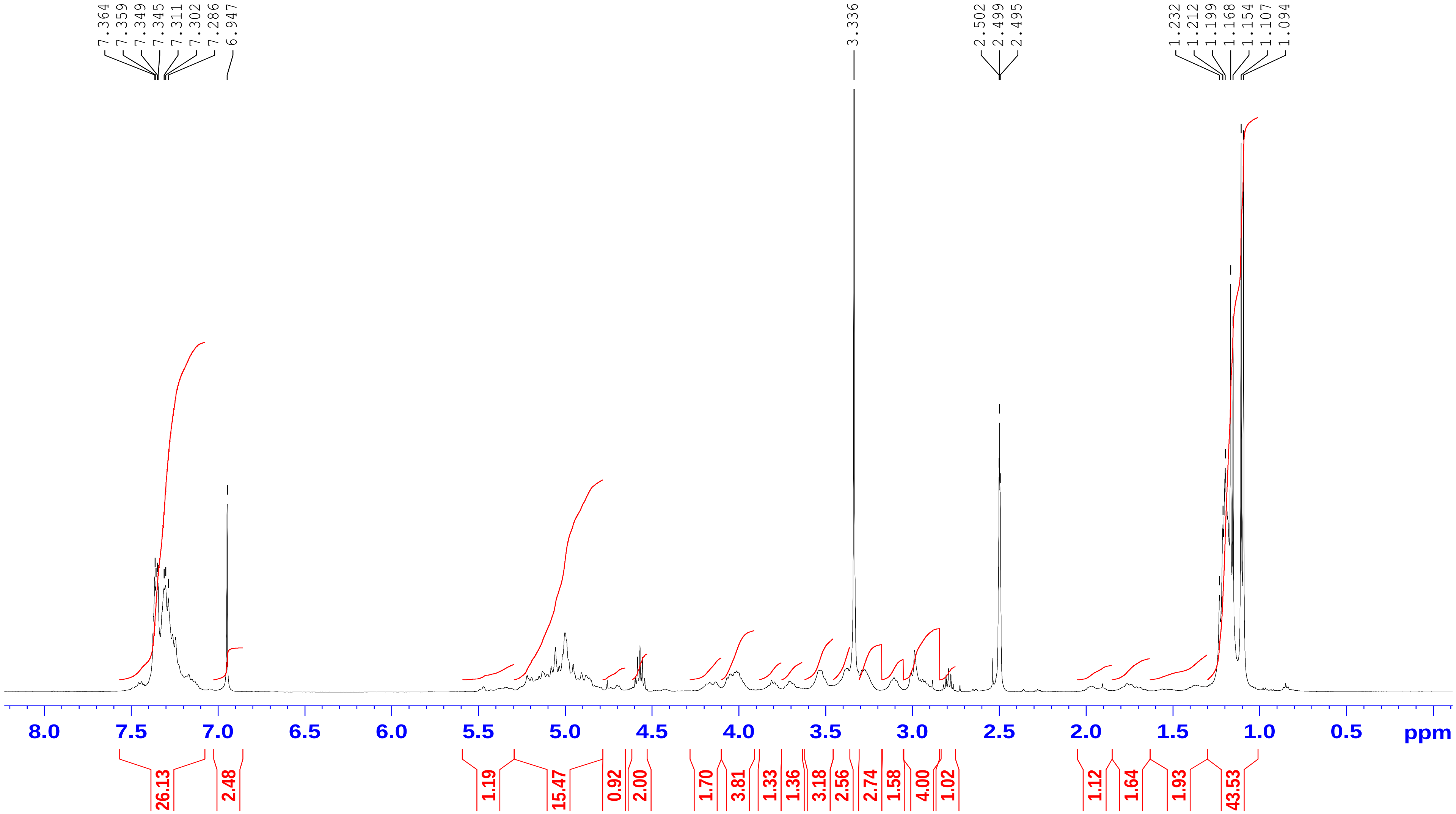

Figure S3. ^{1}H NMR (500.2 MHz, DMSO-*d* 6) spectrum of 4,6,2′,6′,4″-penta-*N*-Cbz-6″-*O*-(2,4,6-triisopropylbenzosulfonyl)apramycin **3**a

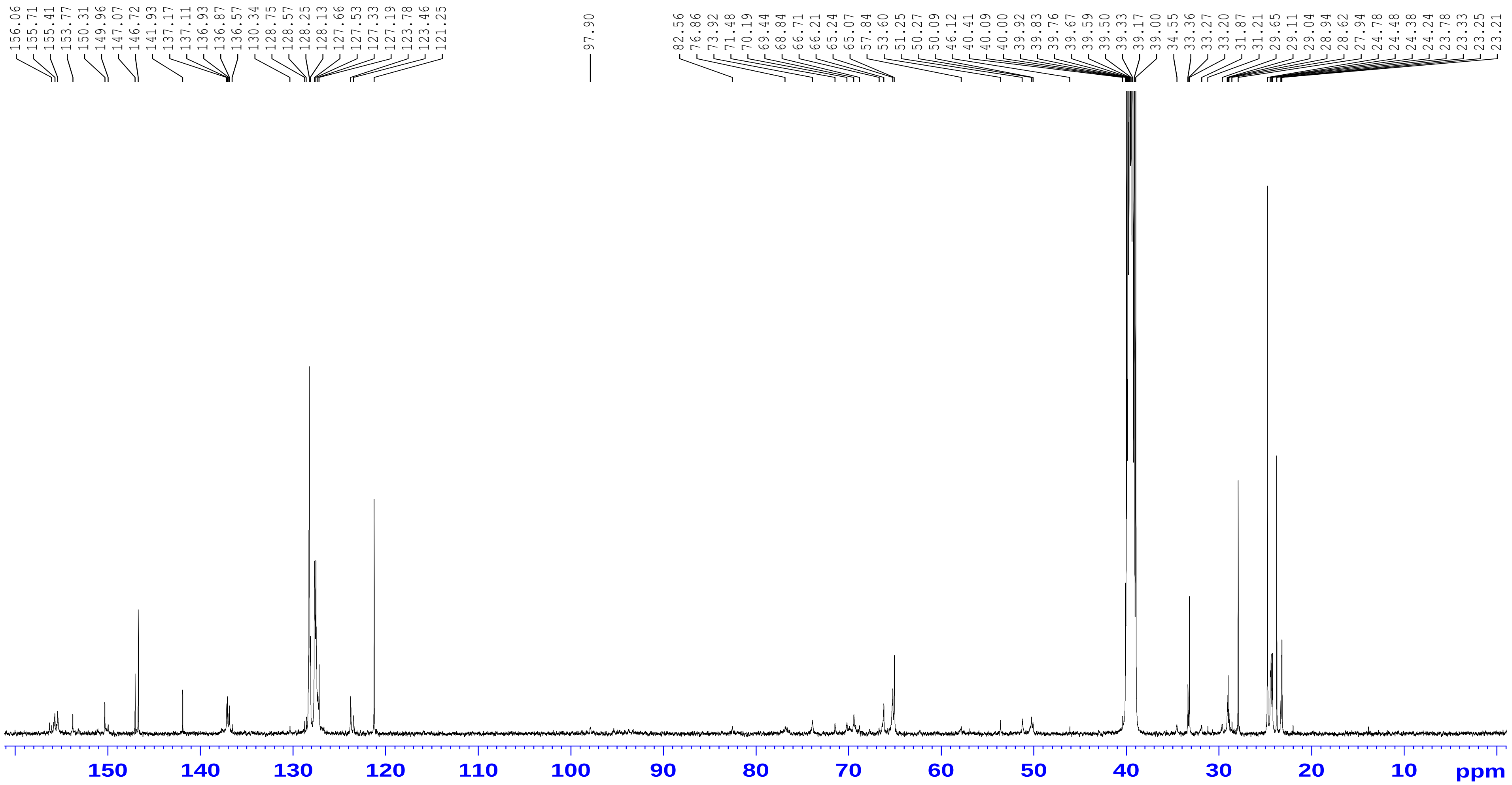

Figure S4. ^{13}C NMR (125.8 MHz, DMSO-d_6) spectrum of of 4,6,2′,6′,4″-penta-*N*-Cbz-6″-*O*-(2,4,6-triisopropylbenzosulfonyl)apramycin **3a**

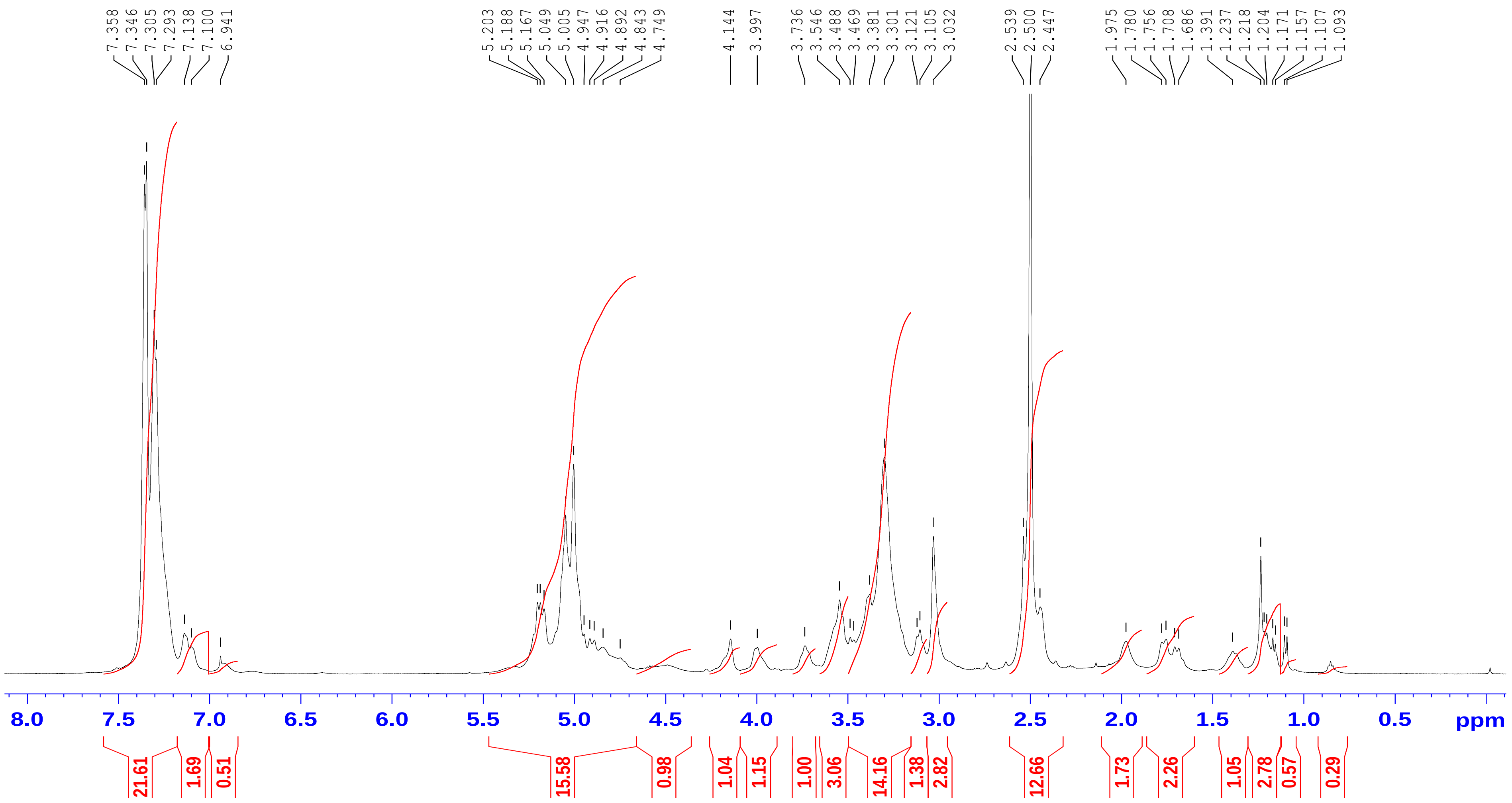

Figure S5. ^{1}H NMR (500.2 MHz, DMSO-d_6) spectrum of 6″-(2-aminoethyamino)-4,6,2′,6′,4″-penta-*N*-Cbz-6″-deoxyapramycin **4a**

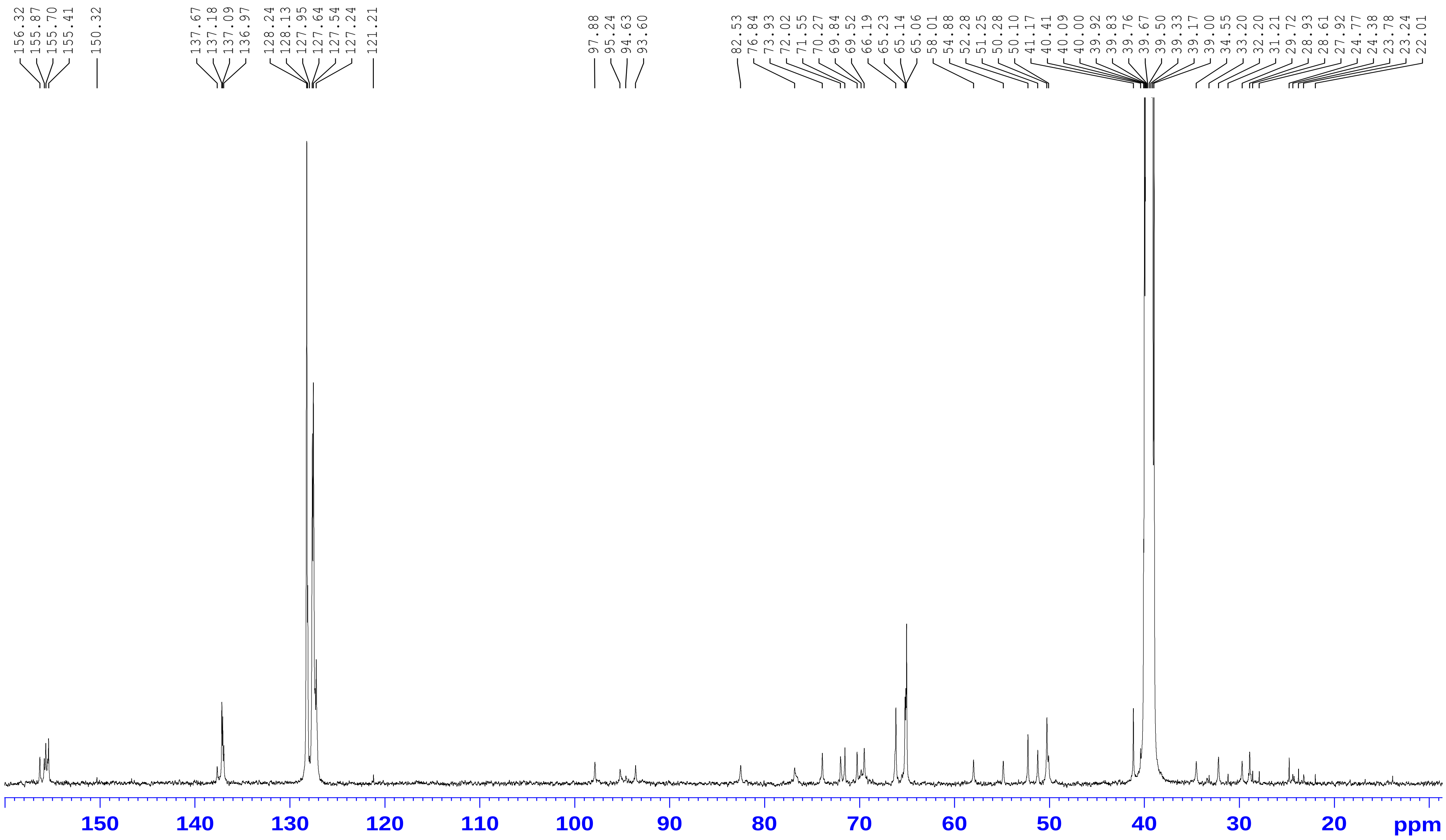

Figure S6. ^{13}C NMR (125.8 MHz, DMSO-d_6) spectrum of 6″-(2-aminoethyamino)-4,6,2′,6′,4″-penta-*N*-Cbz-6″-deoxyapramycin **4a**

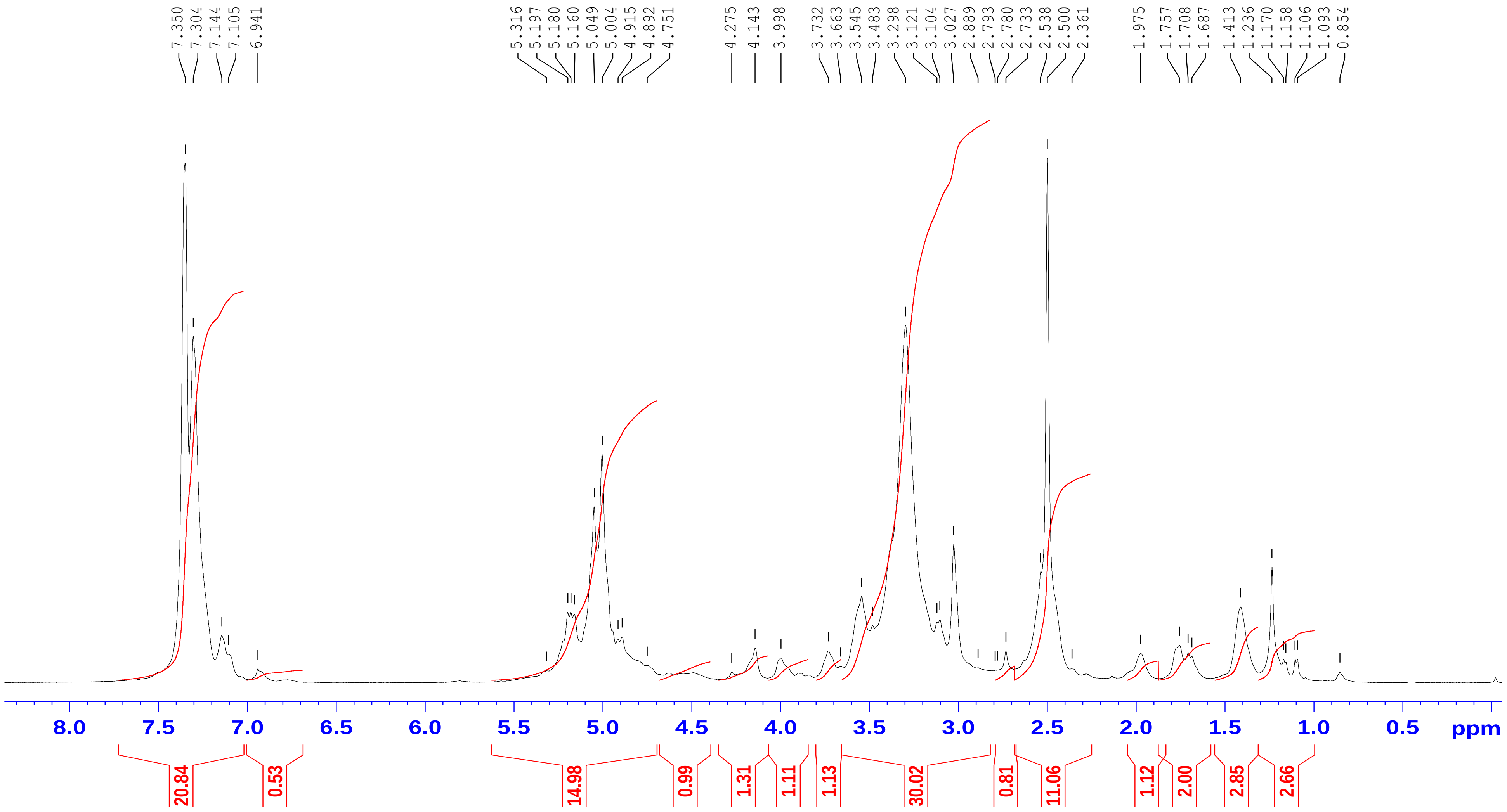

Figure S7. ^{1}H NMR (500.2 MHz, DMSO-d_6) spectrum of 6″-(3-aminopropyl-1-amino)-4,6,2′,6′,4″-penta-*N*-Cbz-6″-deoxyapramycin **4b**

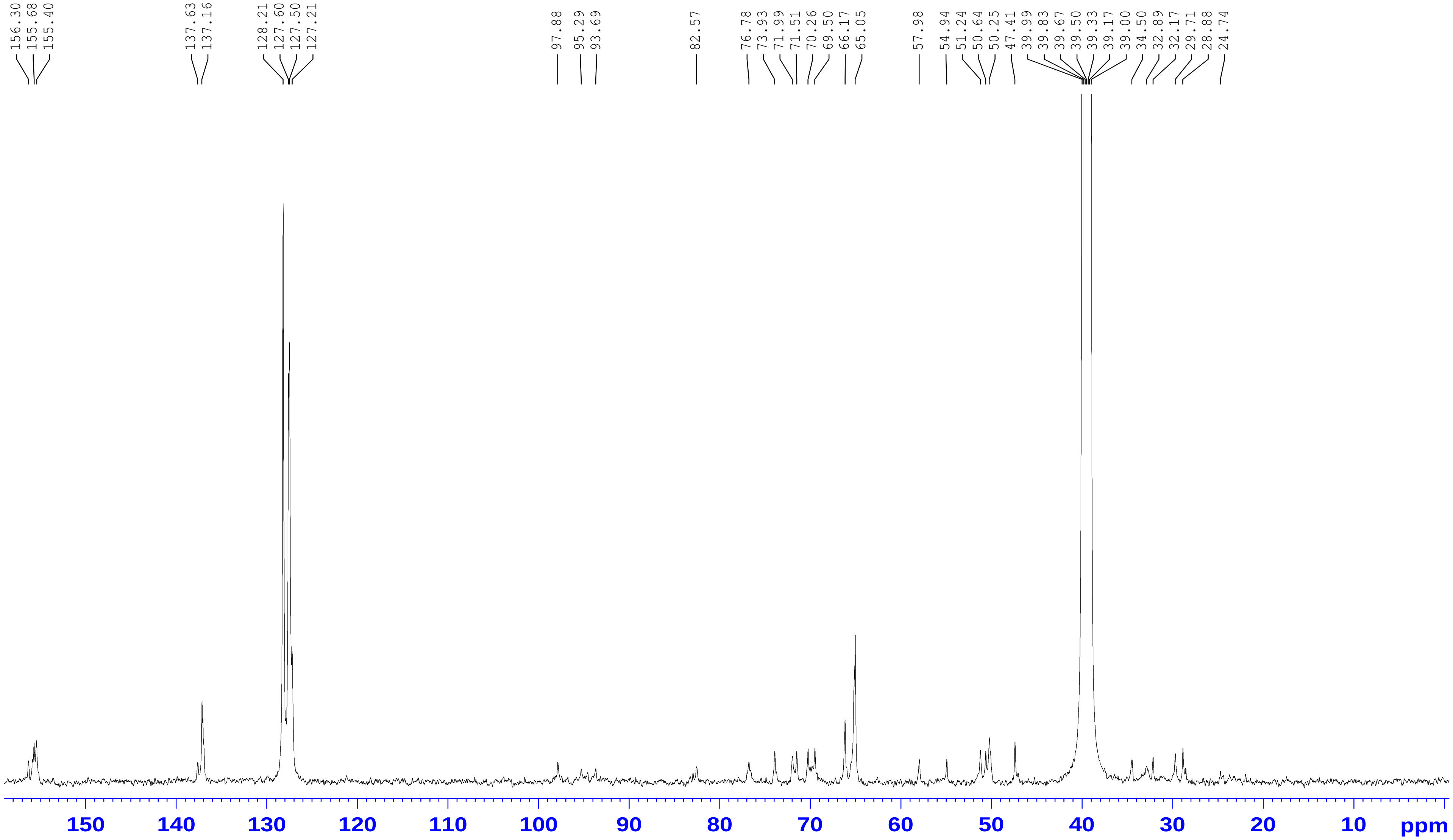

Figure S8. ^{13}C NMR (125.8 MHz, DMSO-*d*$_6$) spectrum of 6″-(3-aminopropyl-1-amino)-4,6,2′,6′,4″-penta-*N*-Cbz-6″-deoxyapramycin **4b**

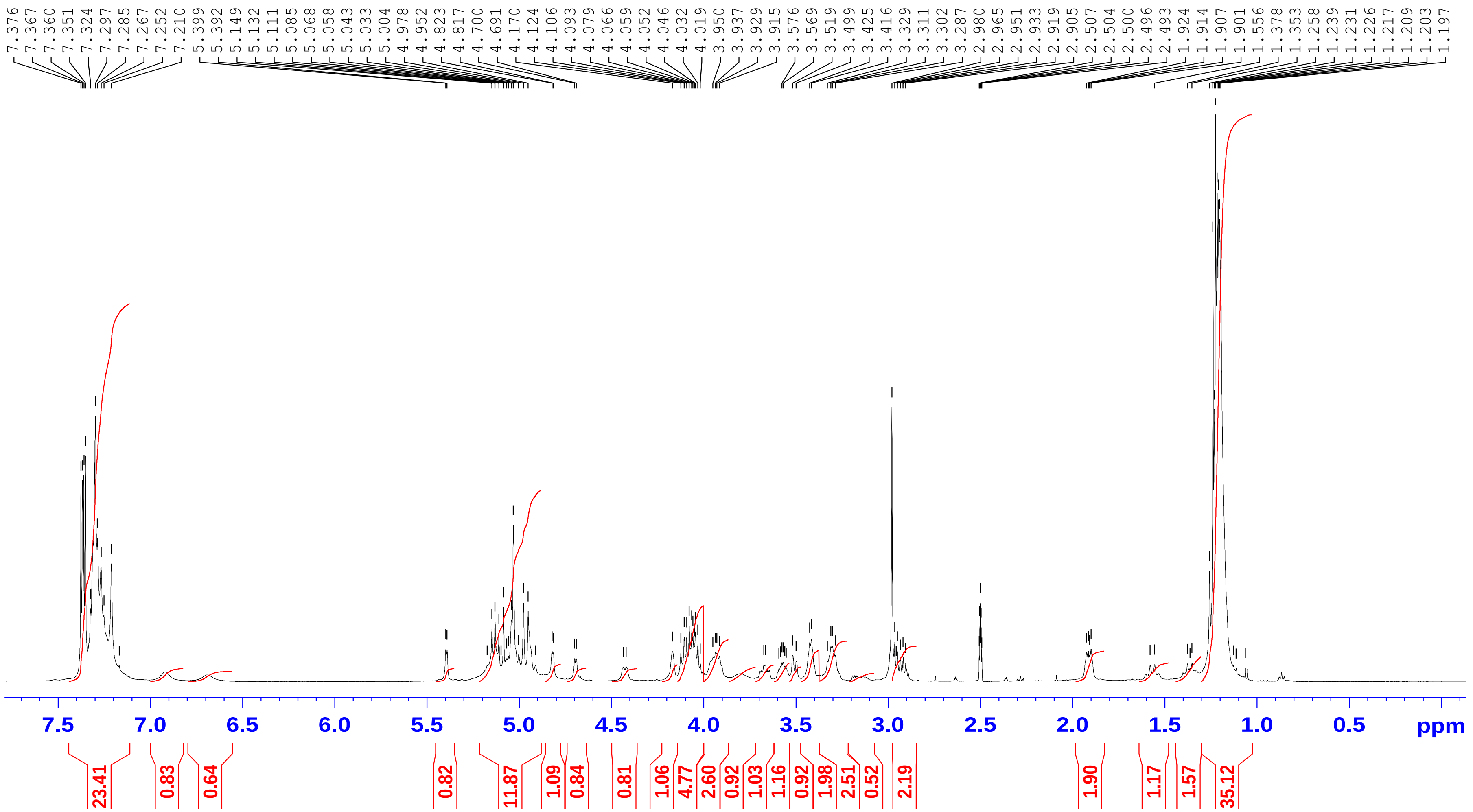

Figure S9. ¹H NMR (500.2 MHz, DMSO-d_6) spectrum of 4,6,2′,6′,4″-penta-*N*-Cbz-2″,6″-(di-*O*-(2,4,6-triisopropylbenzosulfonyl))apramycin **3b**

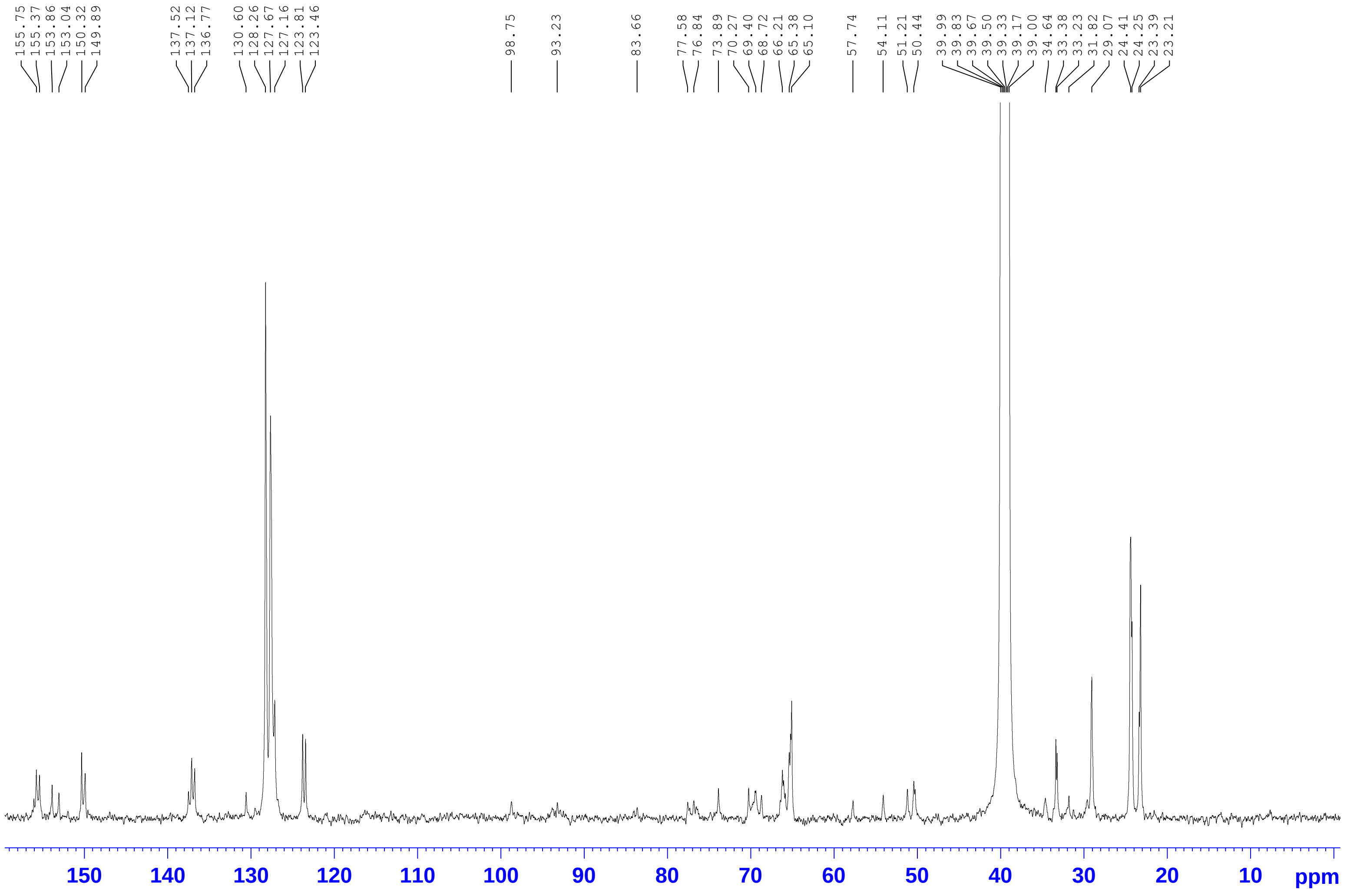

Figure S10. ^{13}C NMR (125.8 MHz, DMSO-d_6) spectrum of 4,6,2′,6′,4″-penta-*N*-Cbz-2″,6″-(di-*O*-(2,4,6-triisopropylbenzosulfonyl))apramycin **3b**

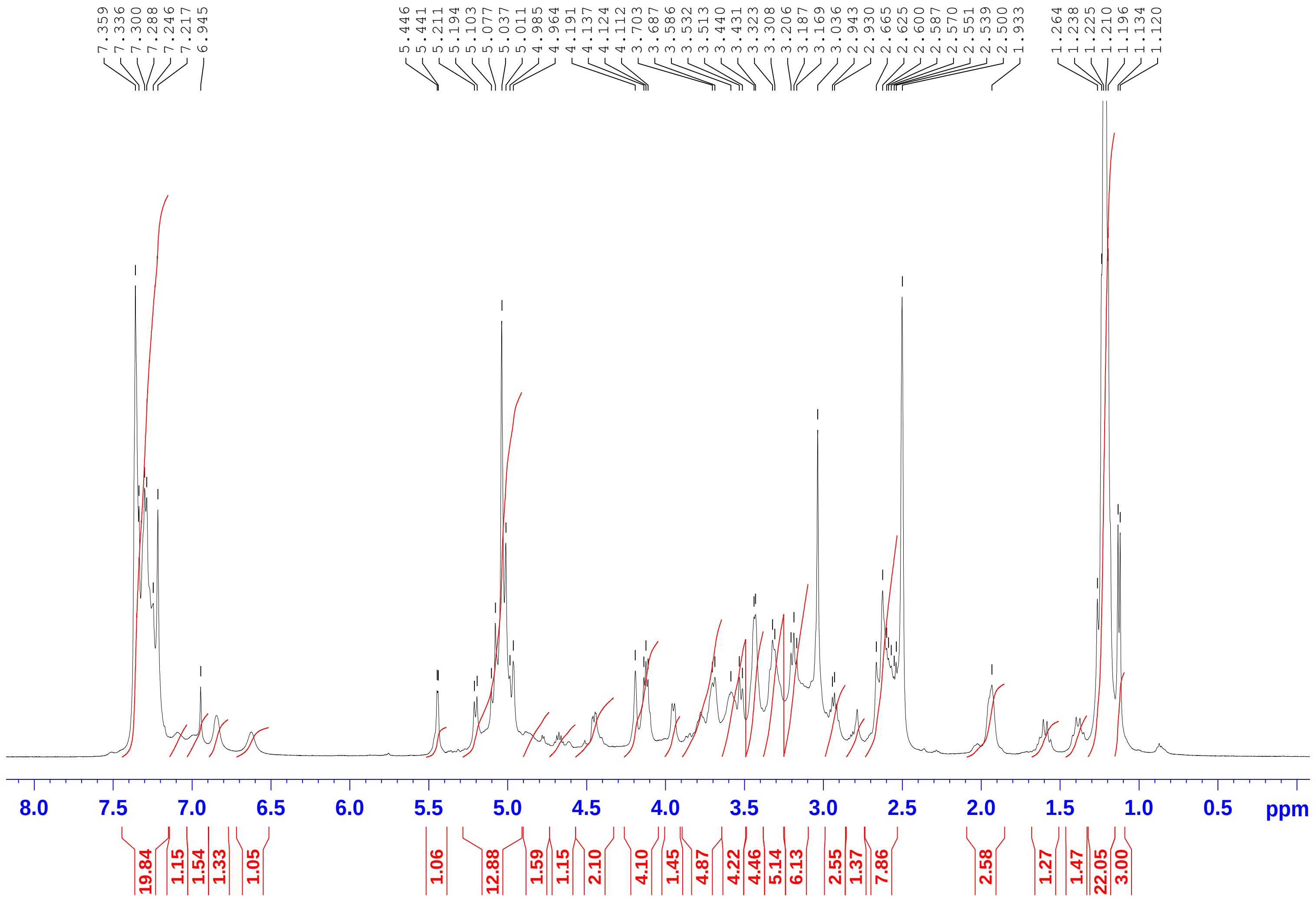

Figure S11. ^{1}H NMR (500.2 MHz, DMSO-*d*6) spectrum of 4,6,2′,6′,4″-penta-*N*-Cbz-2″-*O*-(2,4,6-triisopropylbenzosulfonyl)-6″-(2-aminoethyamino)-6″-deoxyapramycin **6**

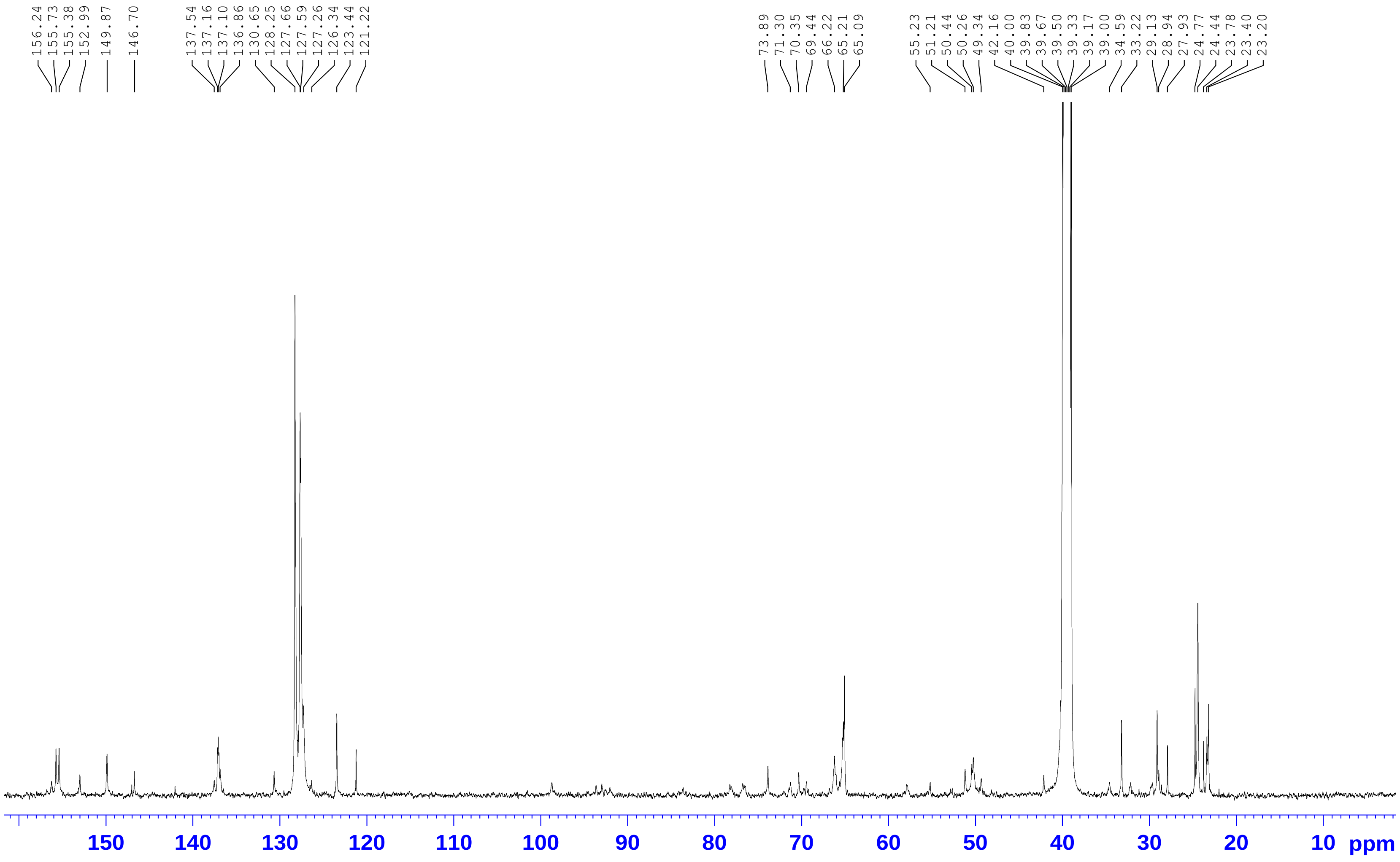

Figure S12. ^{13}C NMR (125.8 MHz, DMSO-*d*6) spectrum of 4,6,2′,6′,4″-penta-*N*-Cbz-2″-*O*-(2,4,6-triisopropylbenzosulfonyl)-6″-(2-aminoethyamino)-6″-deoxyapramycin **6**

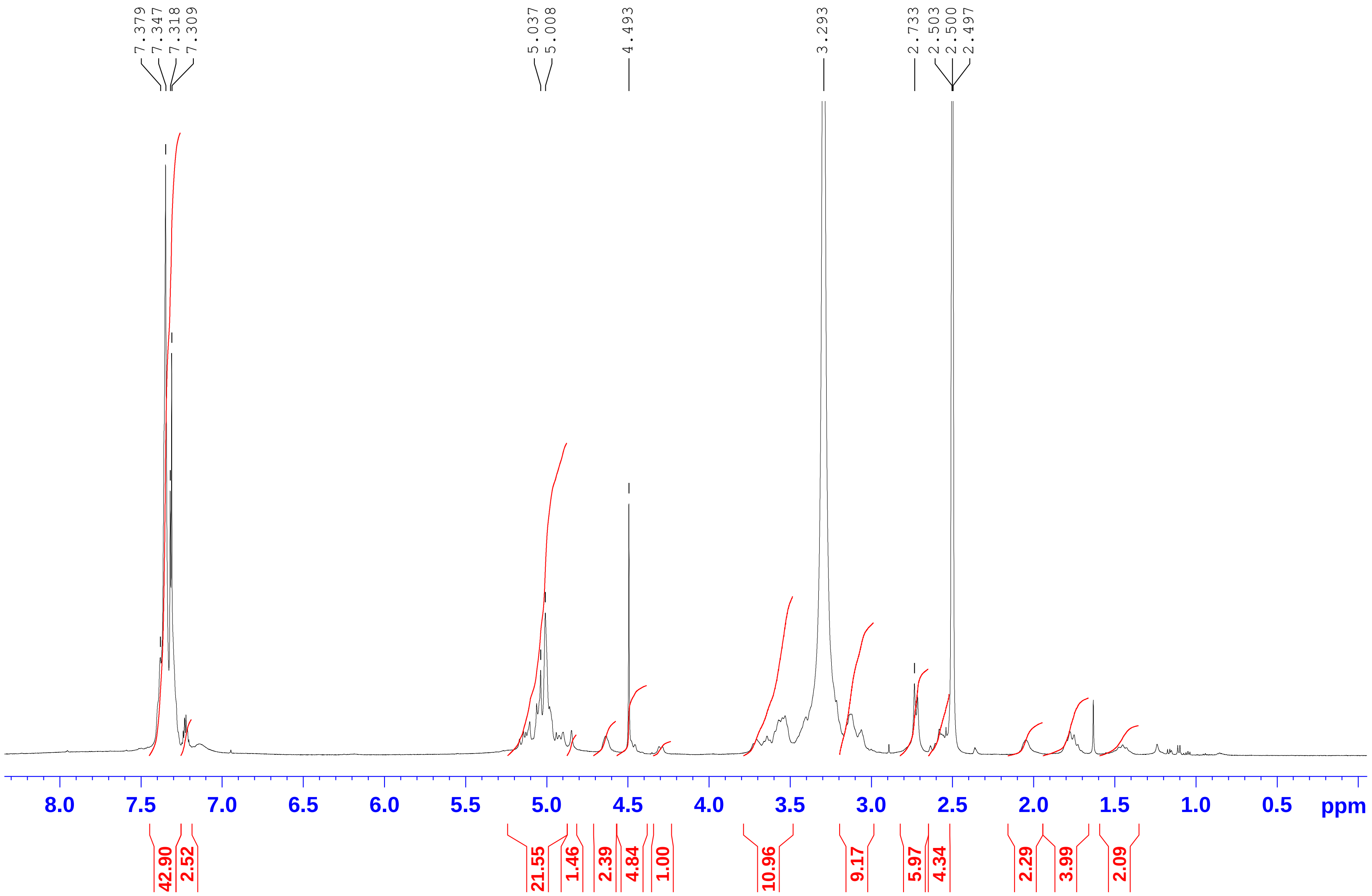

Figure S13. ^{1}H NMR (500.2 MHz, DMSO-*d*6) spectrum of 4,6,2′,6′,4″-penta-*N*-Cbz-6″-(2-guanidinoethylamino)-6″-deoxyapramycin **7a**

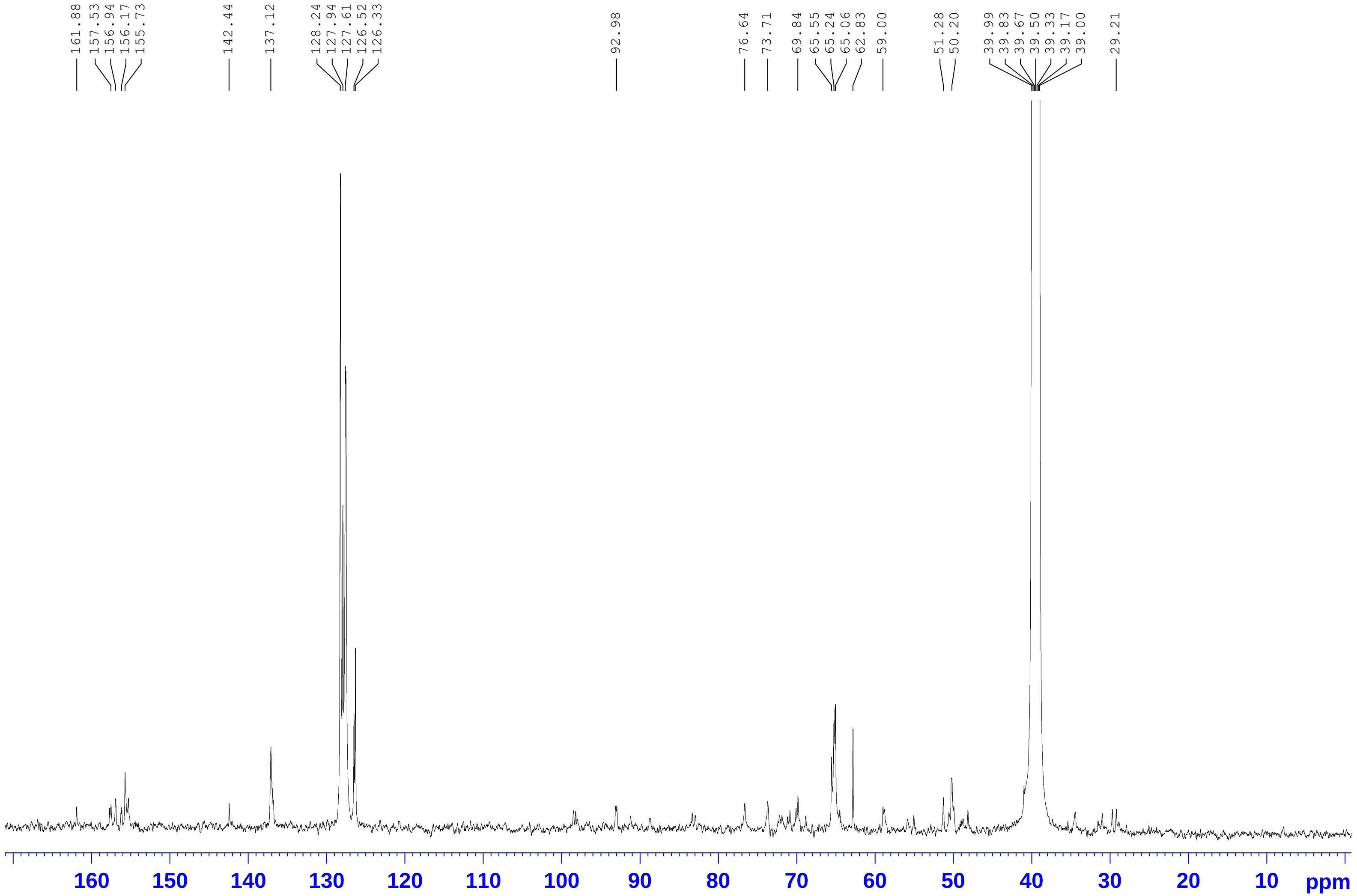

Figure S14. ^{13}C NMR (125.8 MHz, DMSO-d_6) spectrum of 4,6,2′,6′,4″-penta-*N*-Cbz-6″-(2-guanidinoethylamino)-6″-deoxyapramycin **7a**

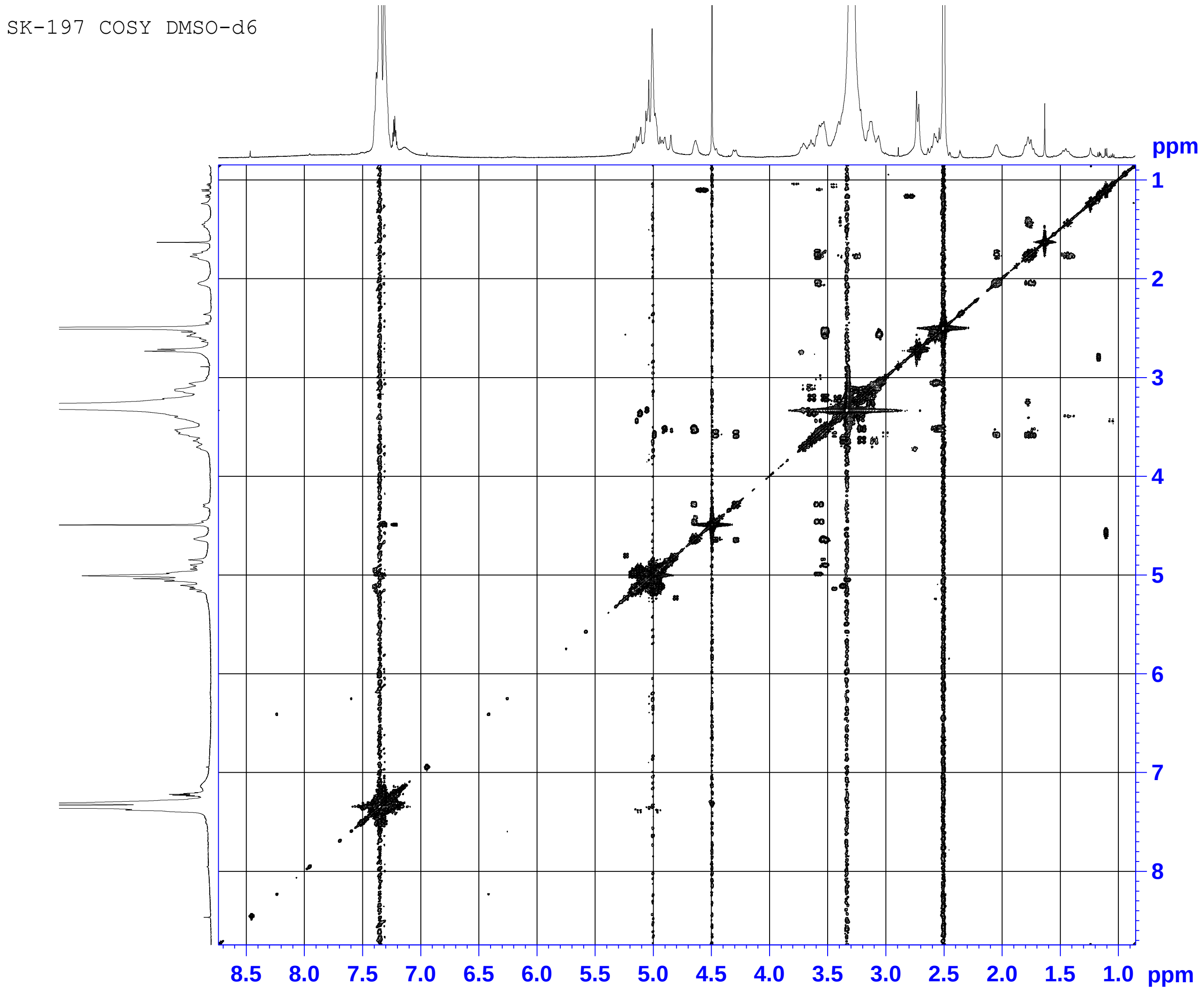

Figure S15. COSY NMR (DMSO-d_6) spectrum of spectrum of 4,6,2′,6′,4″-penta-*N*-Cbz-6″-(2-guanidinoethylamino)-6″-deoxyapramycin **7a**

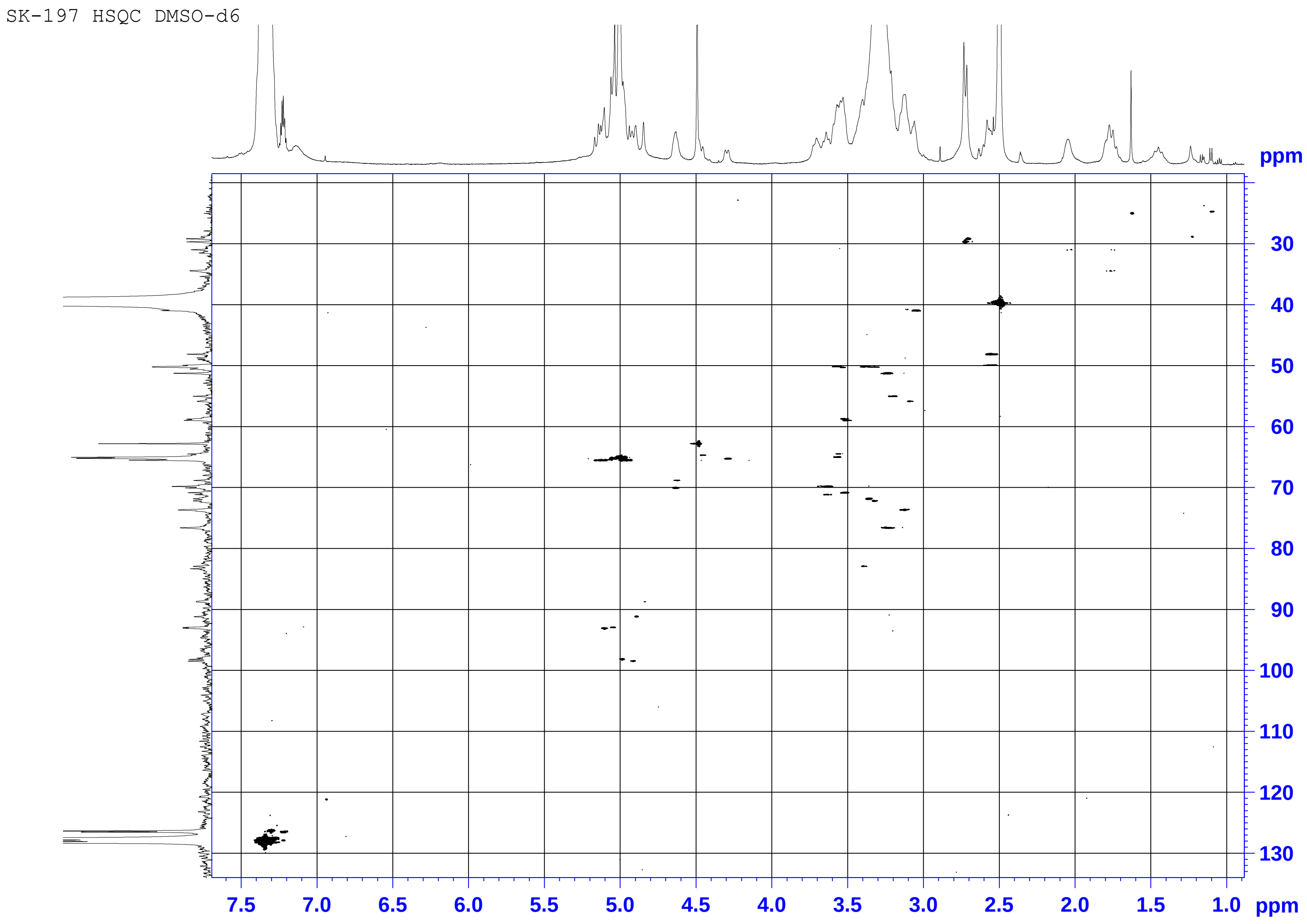

Figure S16. HSQC NMR (DMSO-*d*$_6$) spectrum of 4,6,2′,6′,4″-penta-*N*-Cbz-6″-(2-guanidinoethylamino)-6″-deoxyapramycin **7a**

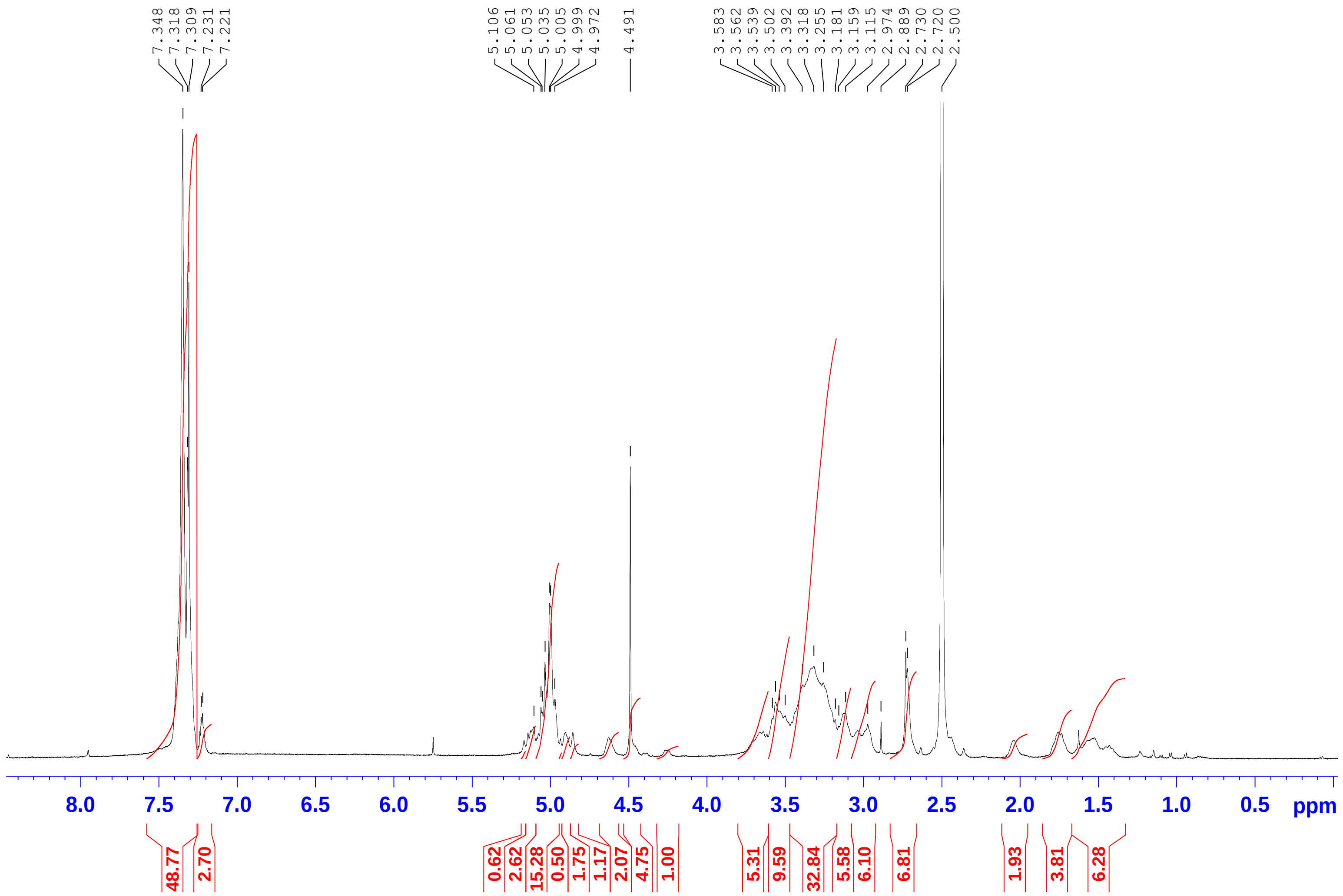

Figure S17. ^{1}H NMR (500.2 MHz, DMSO-d_6) spectrum of 4,6,2′,6′,4″-(penta-N-Cbz)-6″-(3-guanidinpropil-1-amino)-6″-deoxyapramycin **7b**

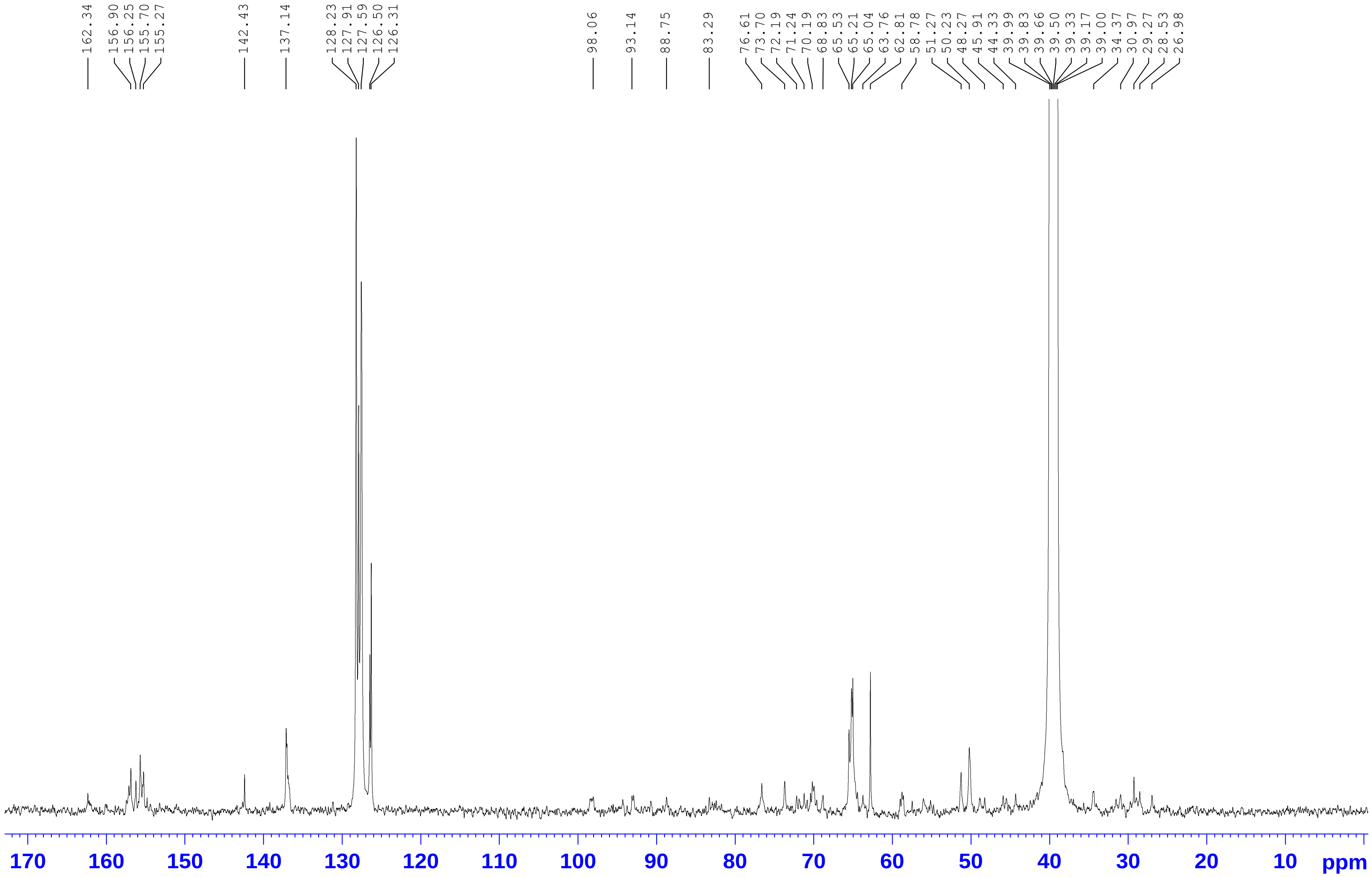

Figure S18. ^{13}C NMR (125.8 MHz, DMSO-d_6) spectrum of 4,6,2′,6′,4″-penta-*N*-Cbz-6″-(3-guanidinpropil-1-amino)-6″-deoxyapramycin **7b**

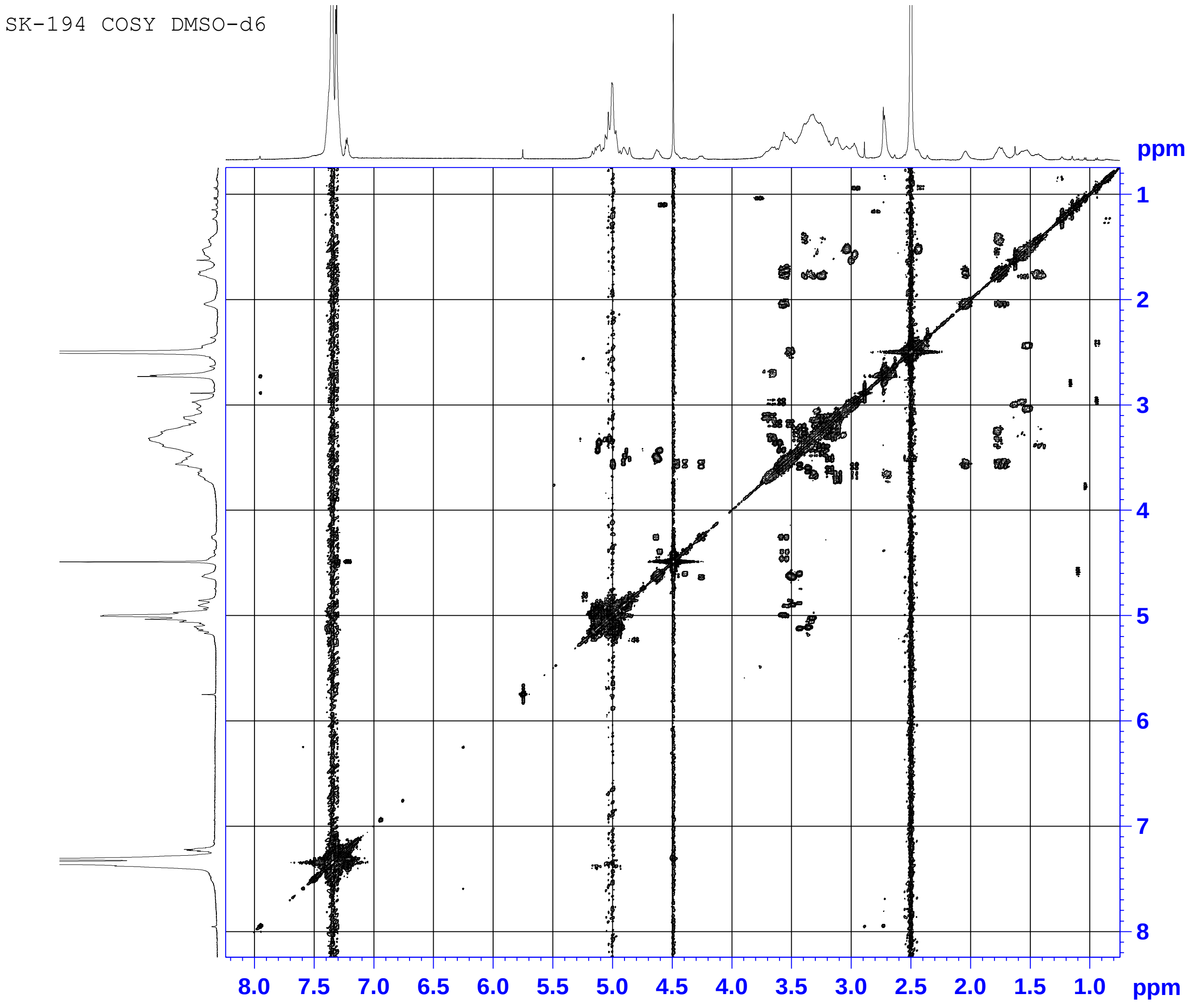

Figure S19. COSY NMR (DMSO-d_6) spectrum of spectrum of 4,6,2′,6′,4″-penta-*N*-Cbz-6″-(3-guanidinpropil-1-amino)-6″-deoxyapramycin **7b**

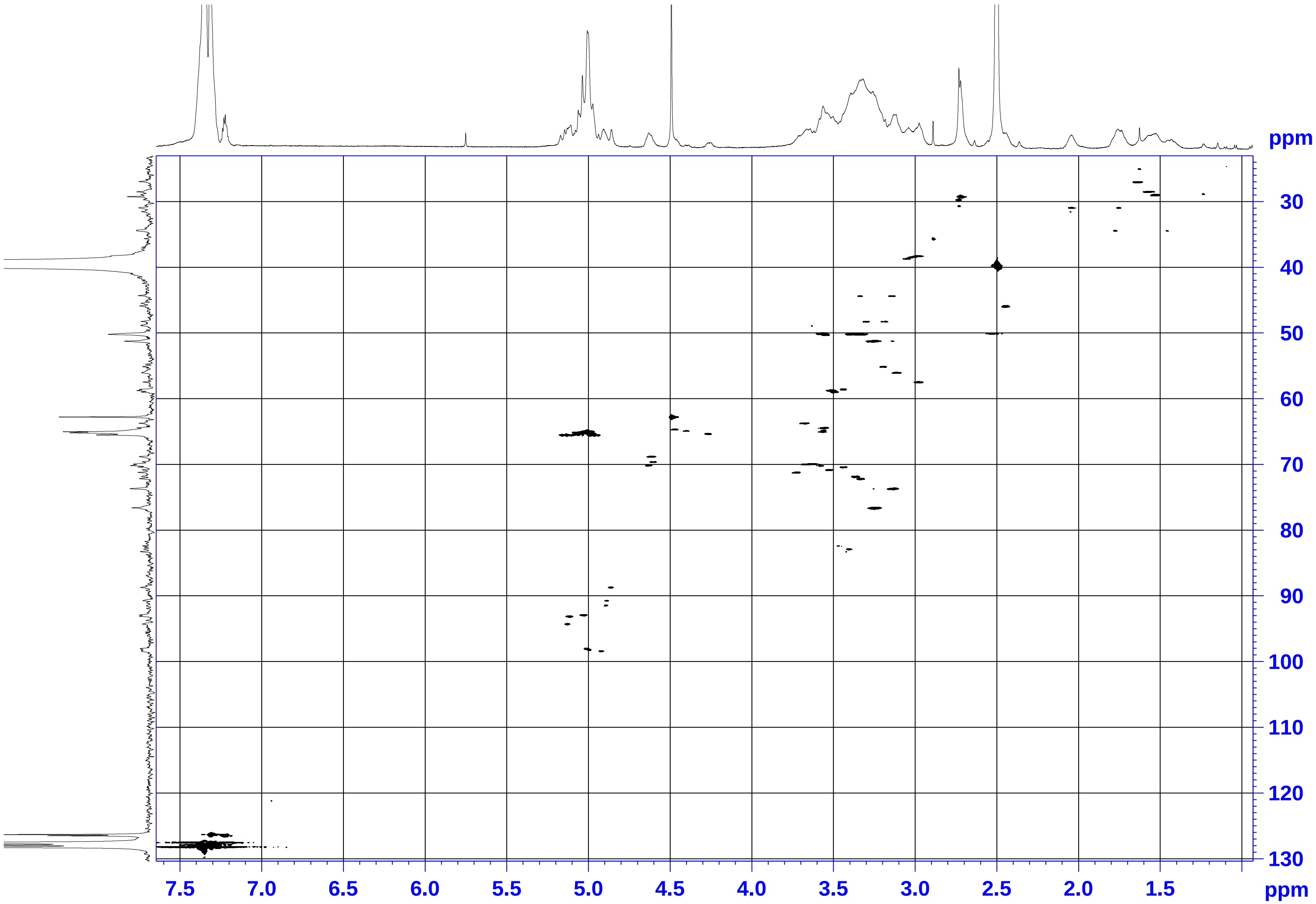

Figure S20. HSQC NMR (DMSO-d_6) spectrum of 4,6,2′,6′,4″-penta-*N*-Cbz-6″-(3-guanidinpropil-1-amino)-6″-deoxyapramycin **7b**

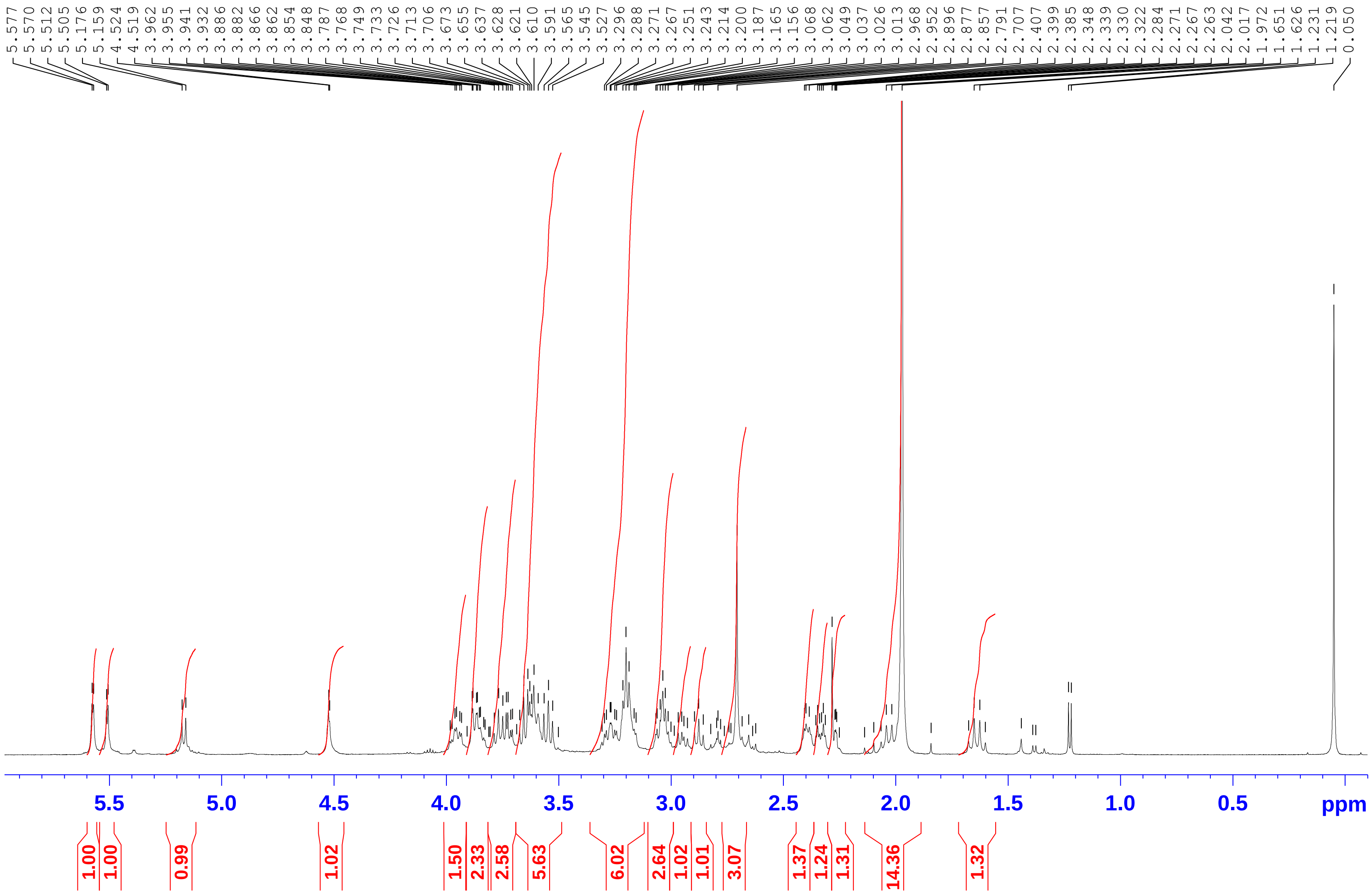

Figure S21. ^{1}H NMR (500.2 MHz, D_2O) spectrum of 6″-(2-aminoethyamino)-6″-deoxyapramycin **5a**

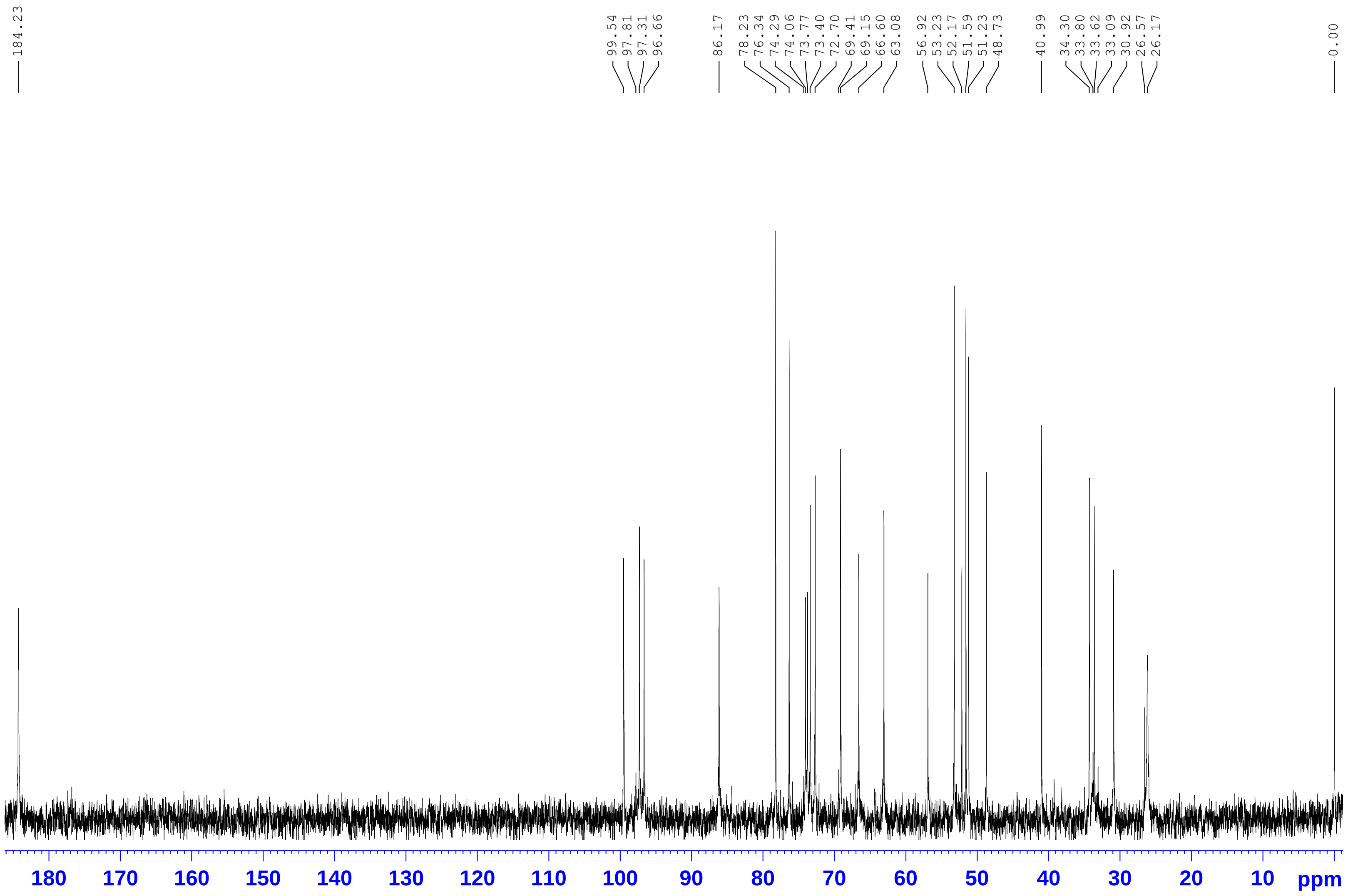

Figure S22. ^{13}C NMR (125.8 MHz, D_2O) spectrum of 6″-(2-aminoethyamino)-6″-deoxyapramycin **5a**

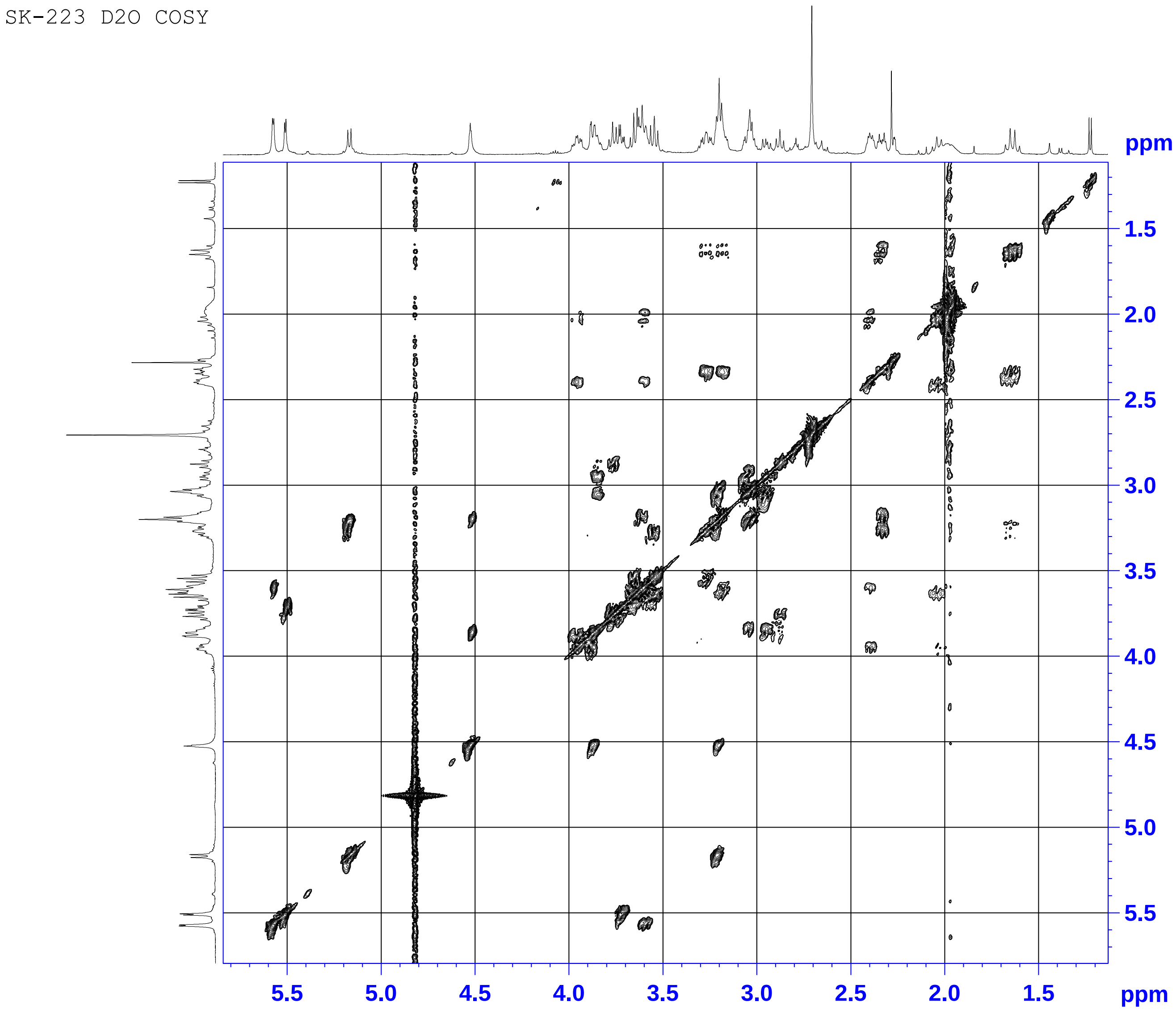

Figure S23. COSY NMR (D_2O) spectrum of spectrum of 6″-(2-aminoethyamino)-6″-deoxyapramycin **5a**

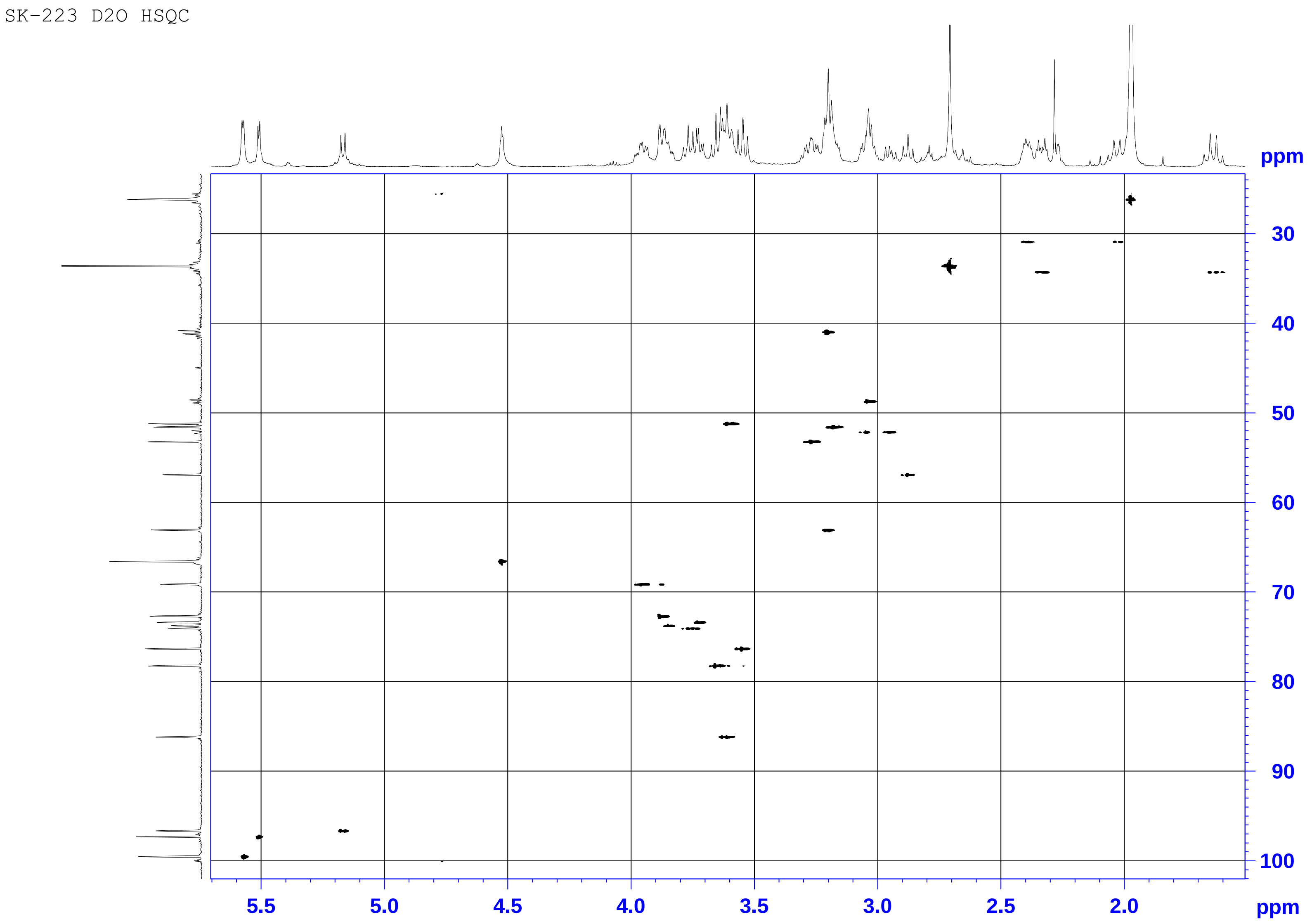

Figure S24. HSQC NMR (D_2O) spectrum of 6″-(2-aminoethyamino)-6″-deoxyapramycin **5a**

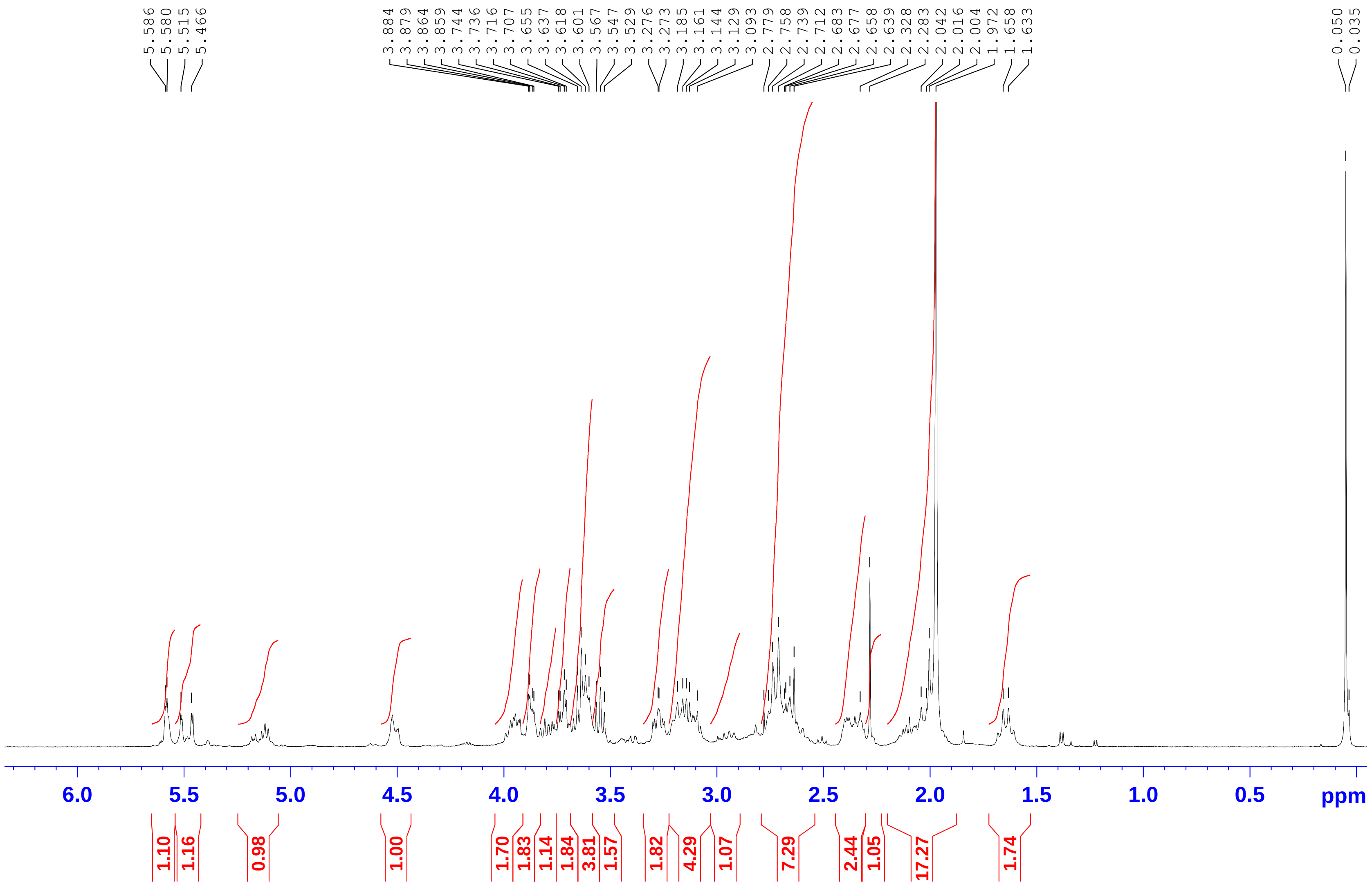

Figure S25. ^{1}H NMR (500.2 MHz, D_2O) spectrum of 6″-(3-aminopropyl-1-amino)-6″-deoxyapramycin **5b**

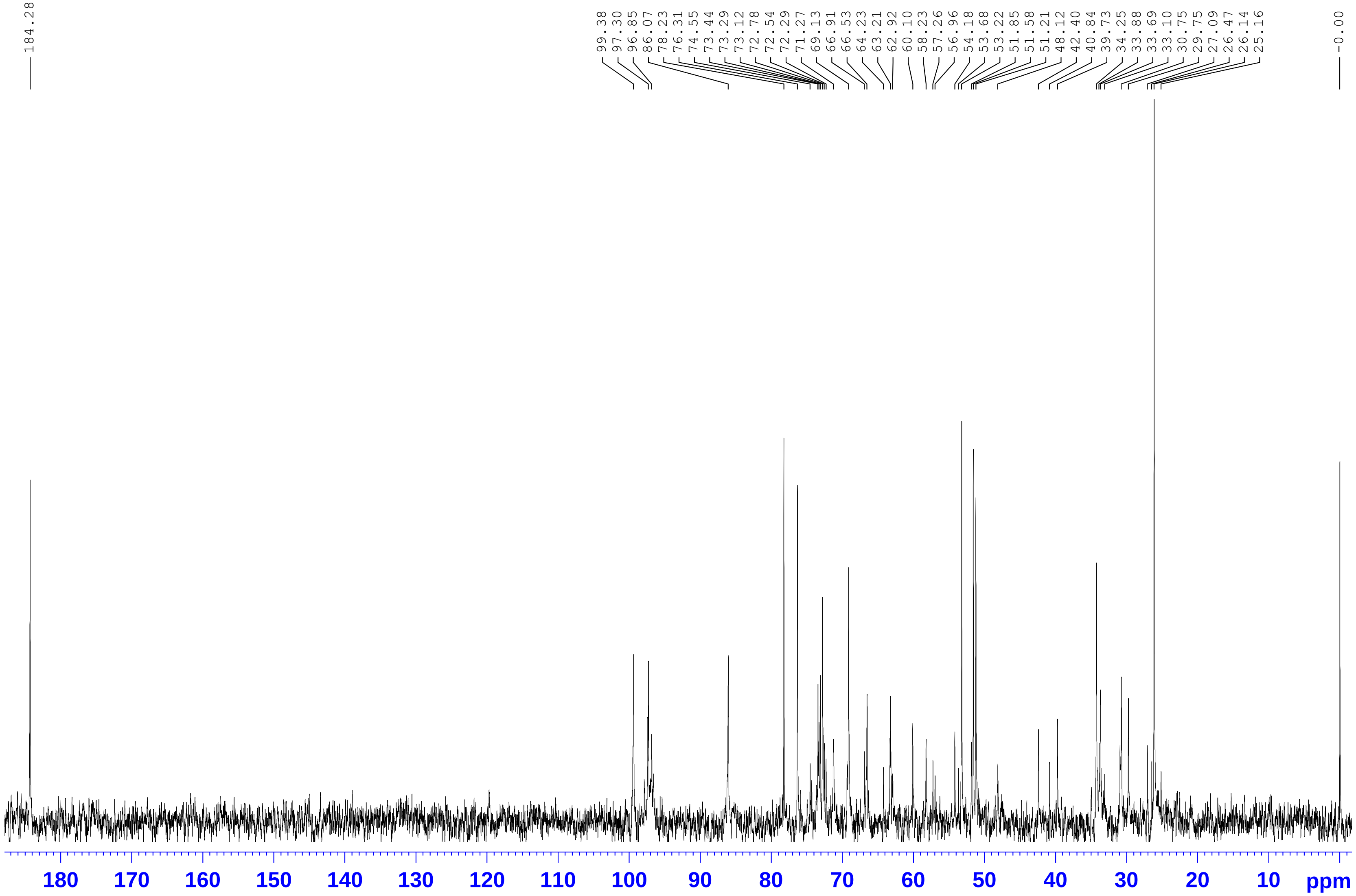

Figure S26. ^{13}C NMR (125.8 MHz, D_2O) spectrum of 6″-(3-aminopropyl-1-amino)-6″-deoxyapramycin **5b**

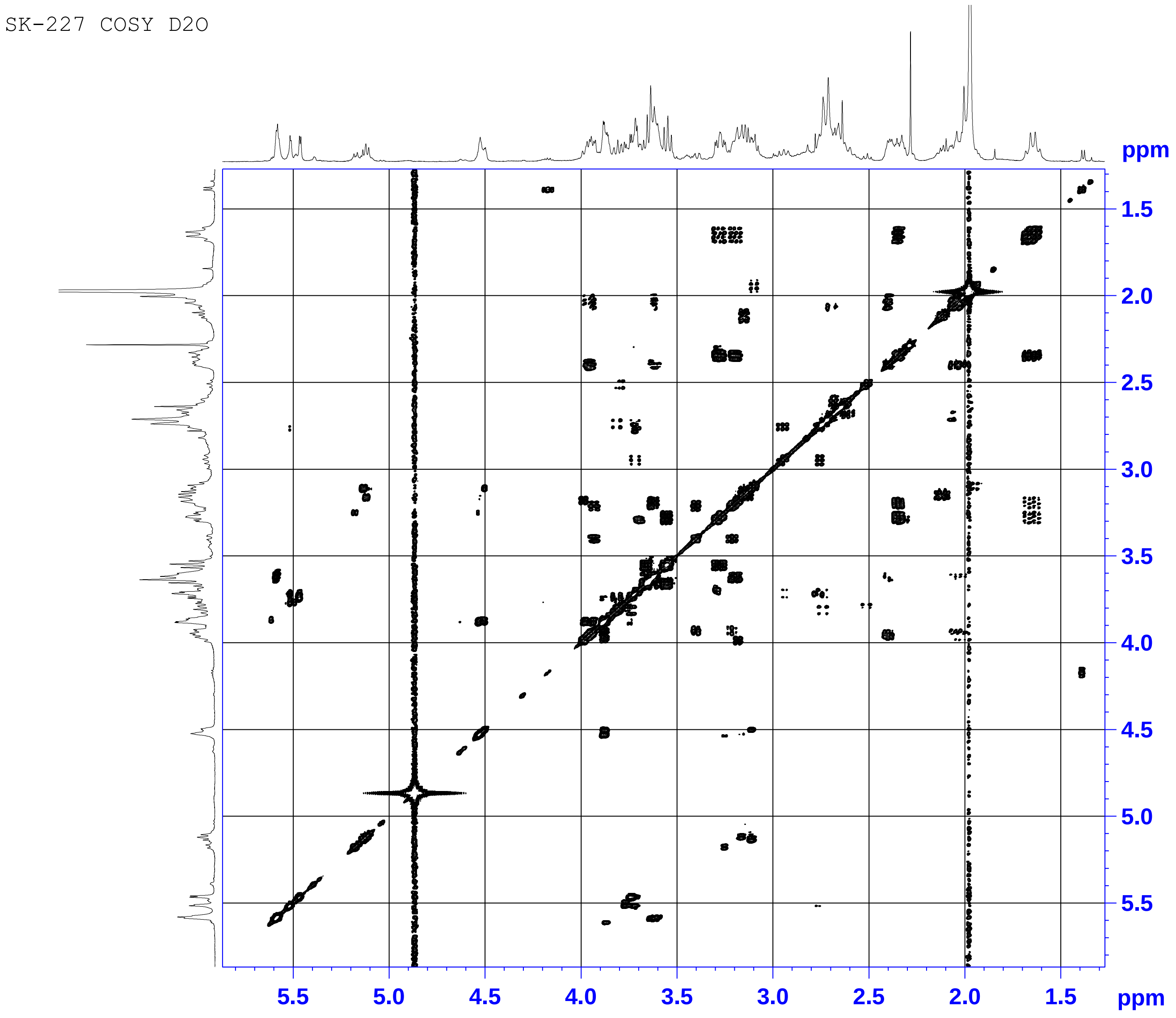

Figure S27. COSY NMR (D_2O) spectrum of 6″-(3-aminopropyl-1-amino)-6″-deoxyapramycin **5b**

SK-227 HSQC D2O

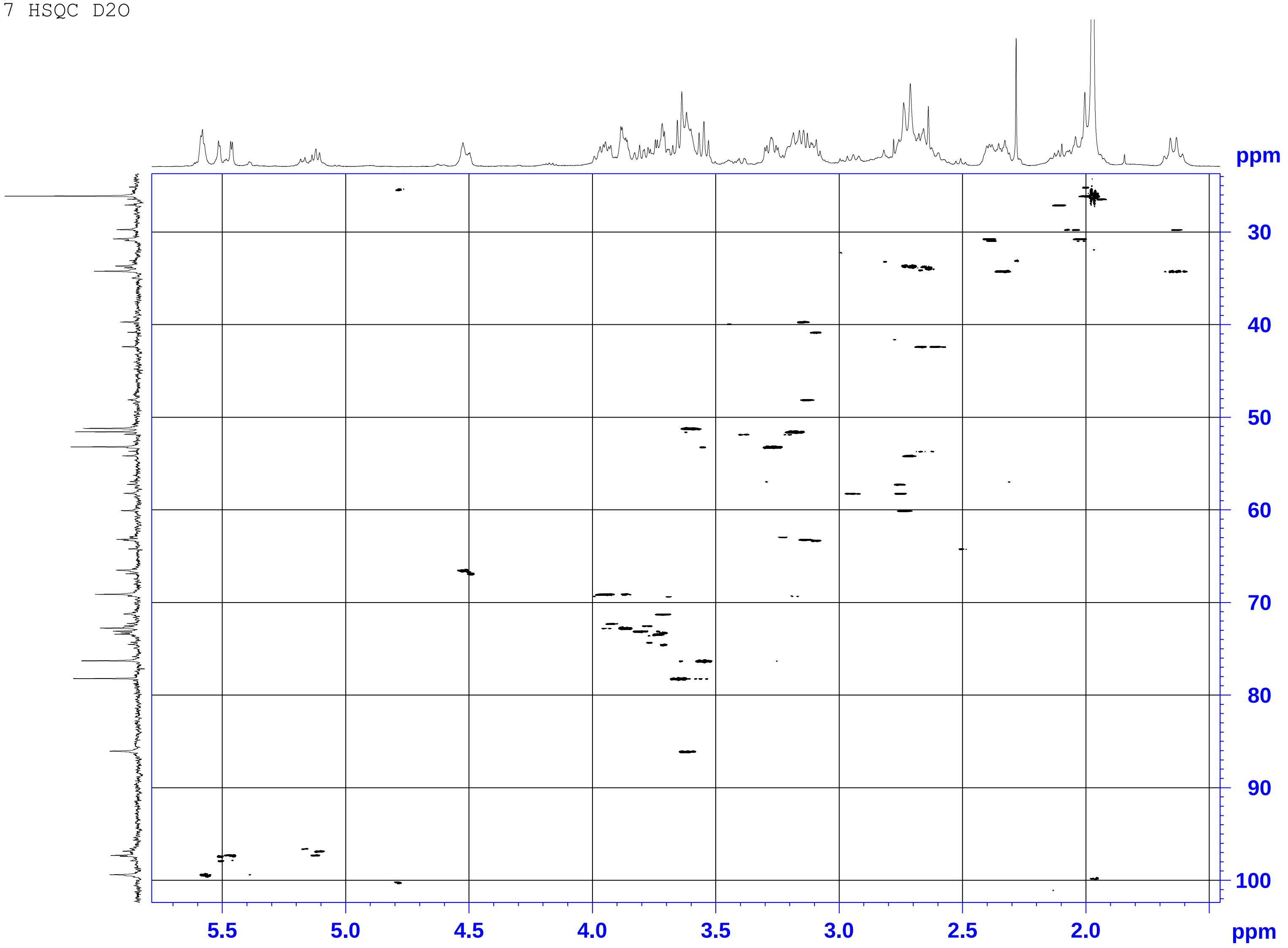

Figure S28. HSQC NMR (D_2O) spectrum of 6″-(3-aminopropyl-1-amino)-6″-deoxyapramycin **5b**

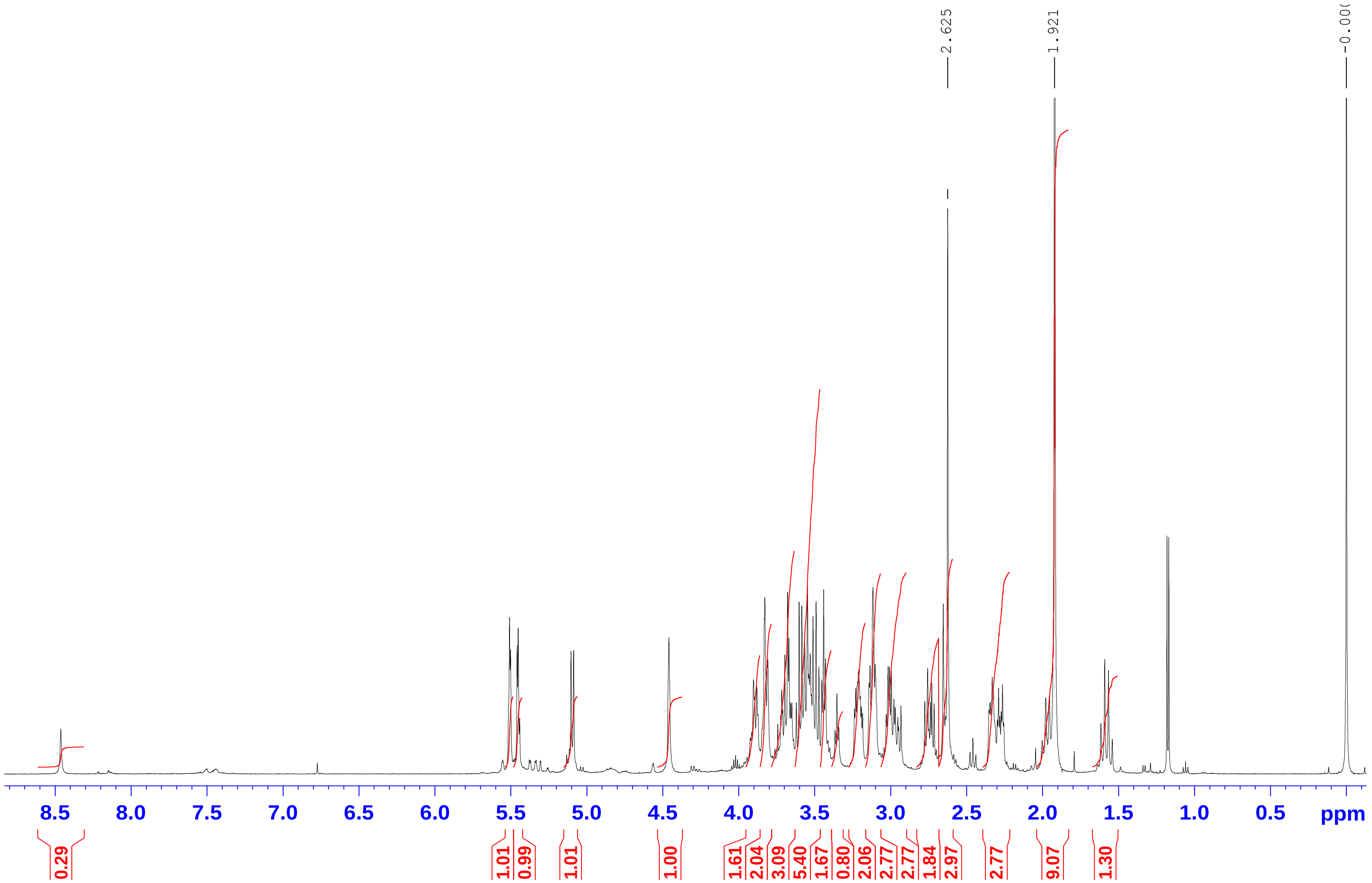

Figure S29. 1H NMR (500.2 MHz, D2O) spectrum of 6″-(2-guanidinoethylamino)-6″-deoxyapramycin **8a**

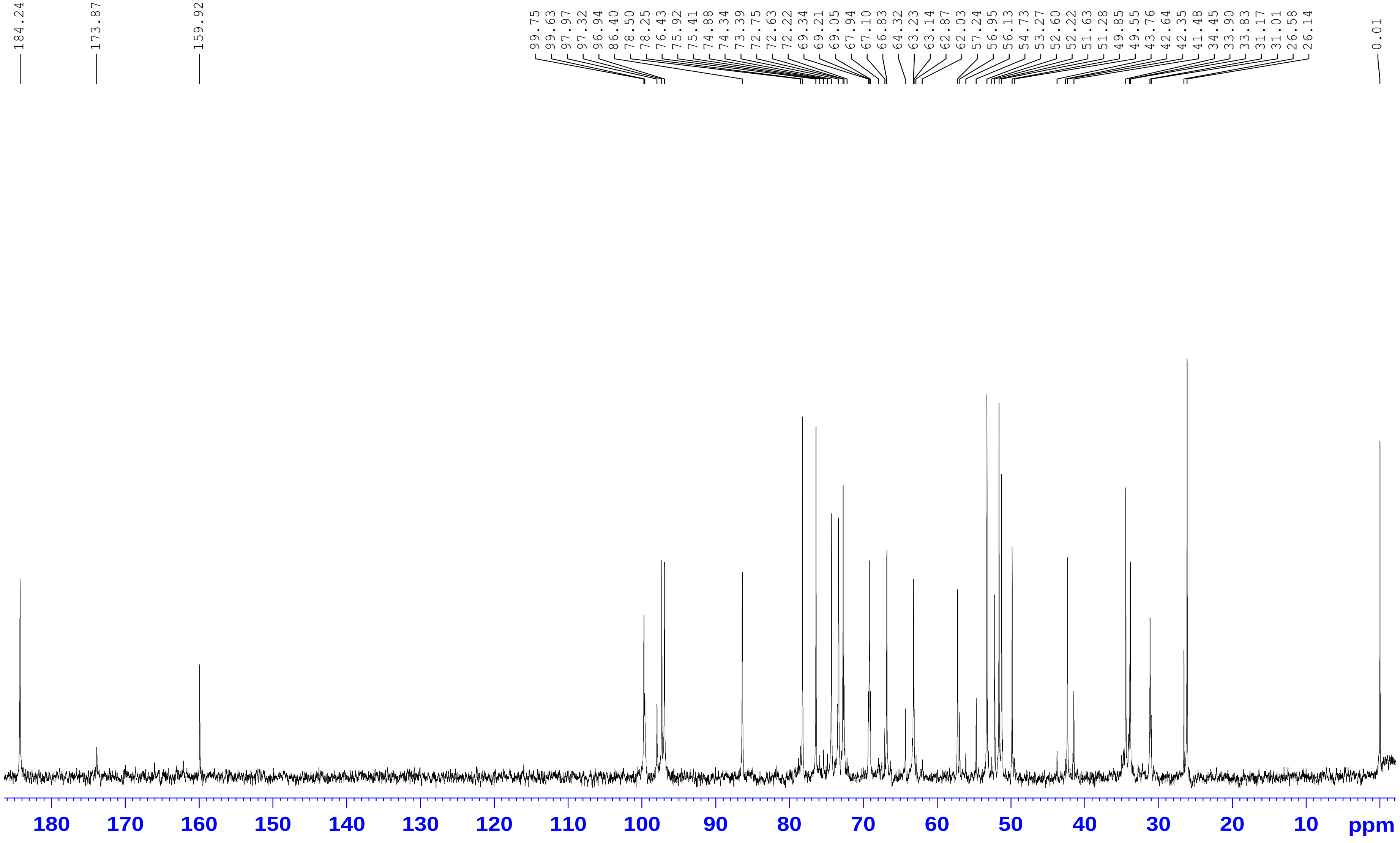

Figure S30. ^{13}C NMR (125.8 MHz, D_2O) spectrum of 6″-(2-guanidinoethylamino)-6″-deoxyapramycin **8a**

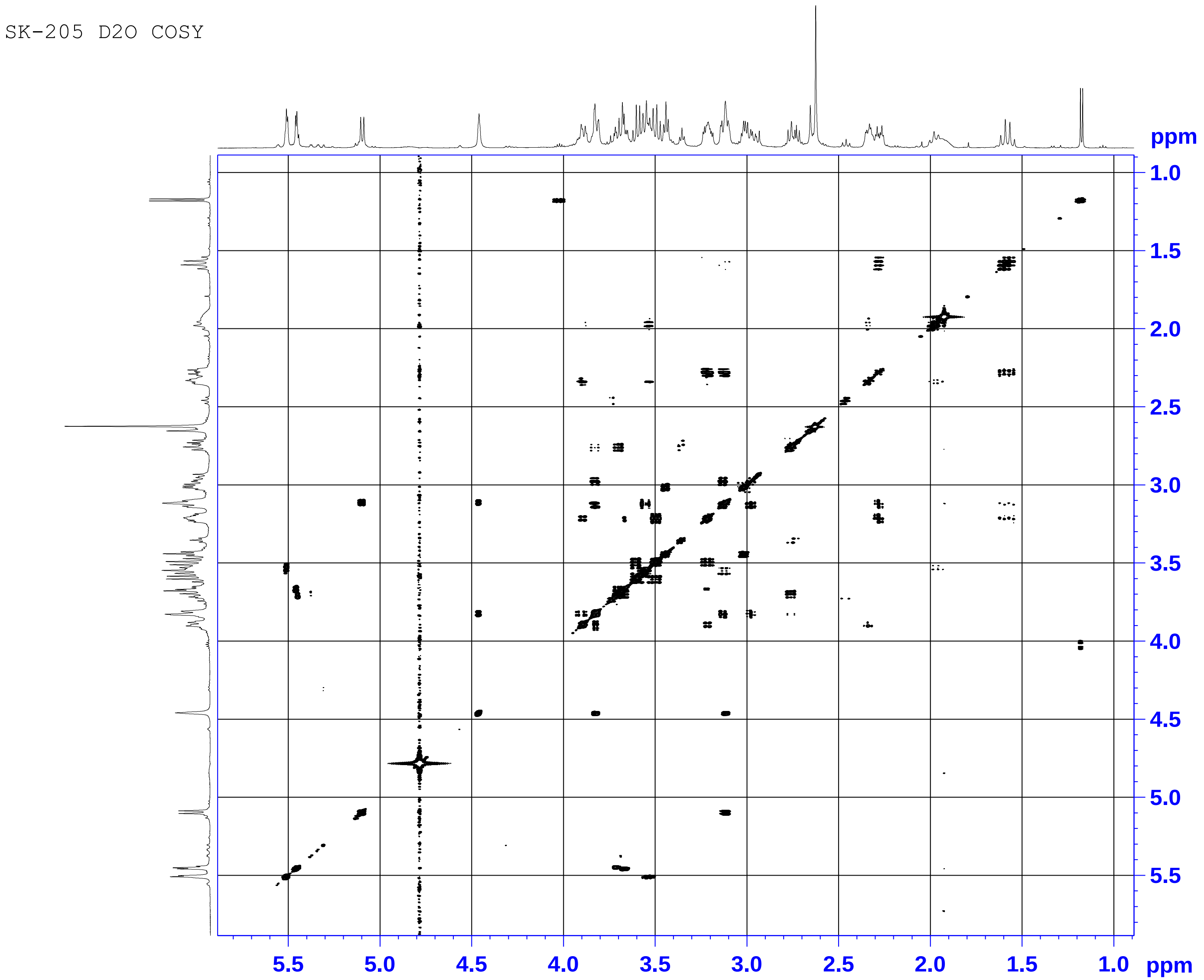

Figure S31. COSY NMR (D_2O) spectrum of spectrum of 6″-(2-guanidinoethylamino)-6″-deoxyapramycin **8a**

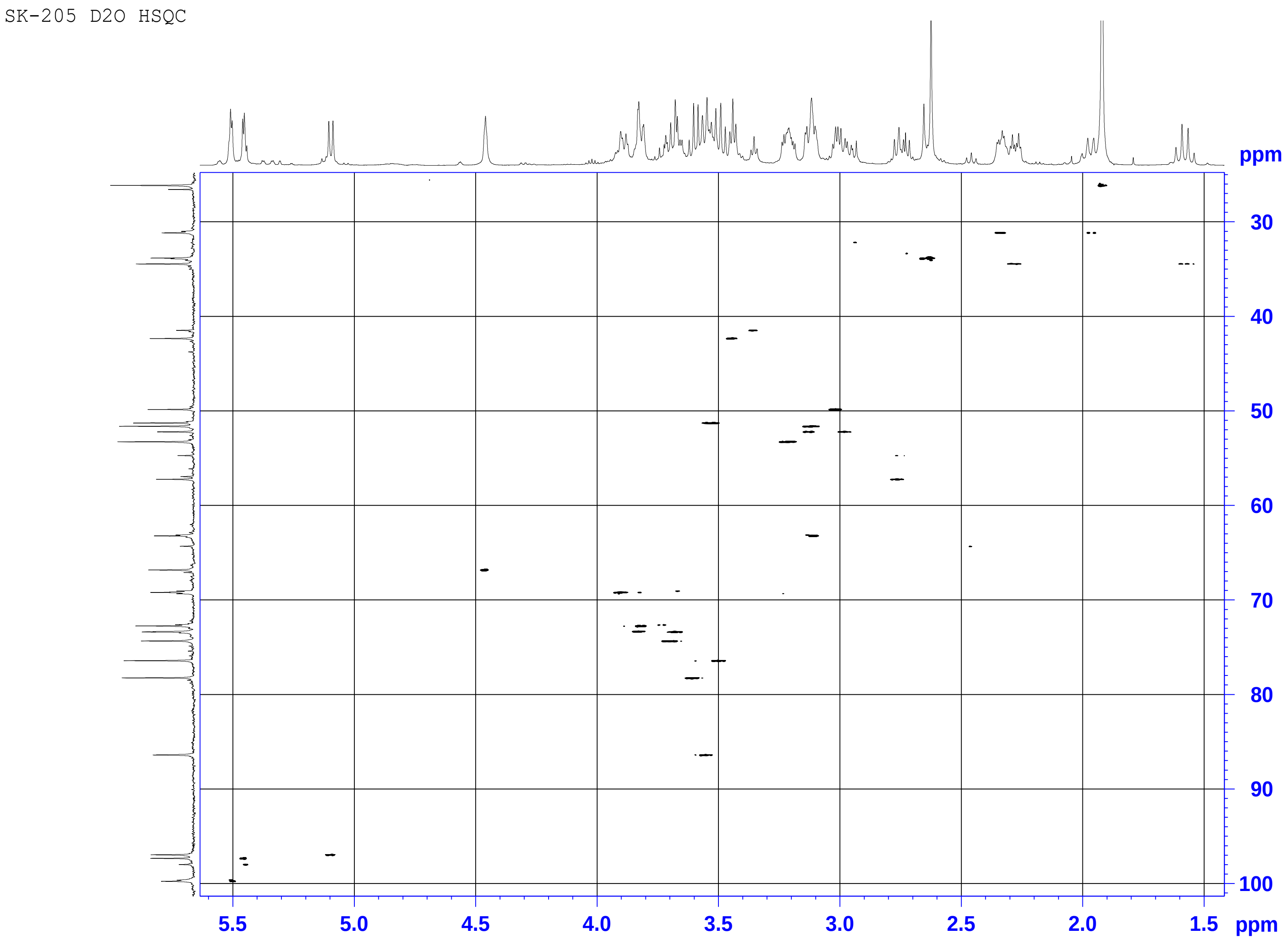

Figure S32. HSQC NMR (D_2O) spectrum of 6″-(2-guanidinoethylamino)-6″-deoxyapramycin **8a**

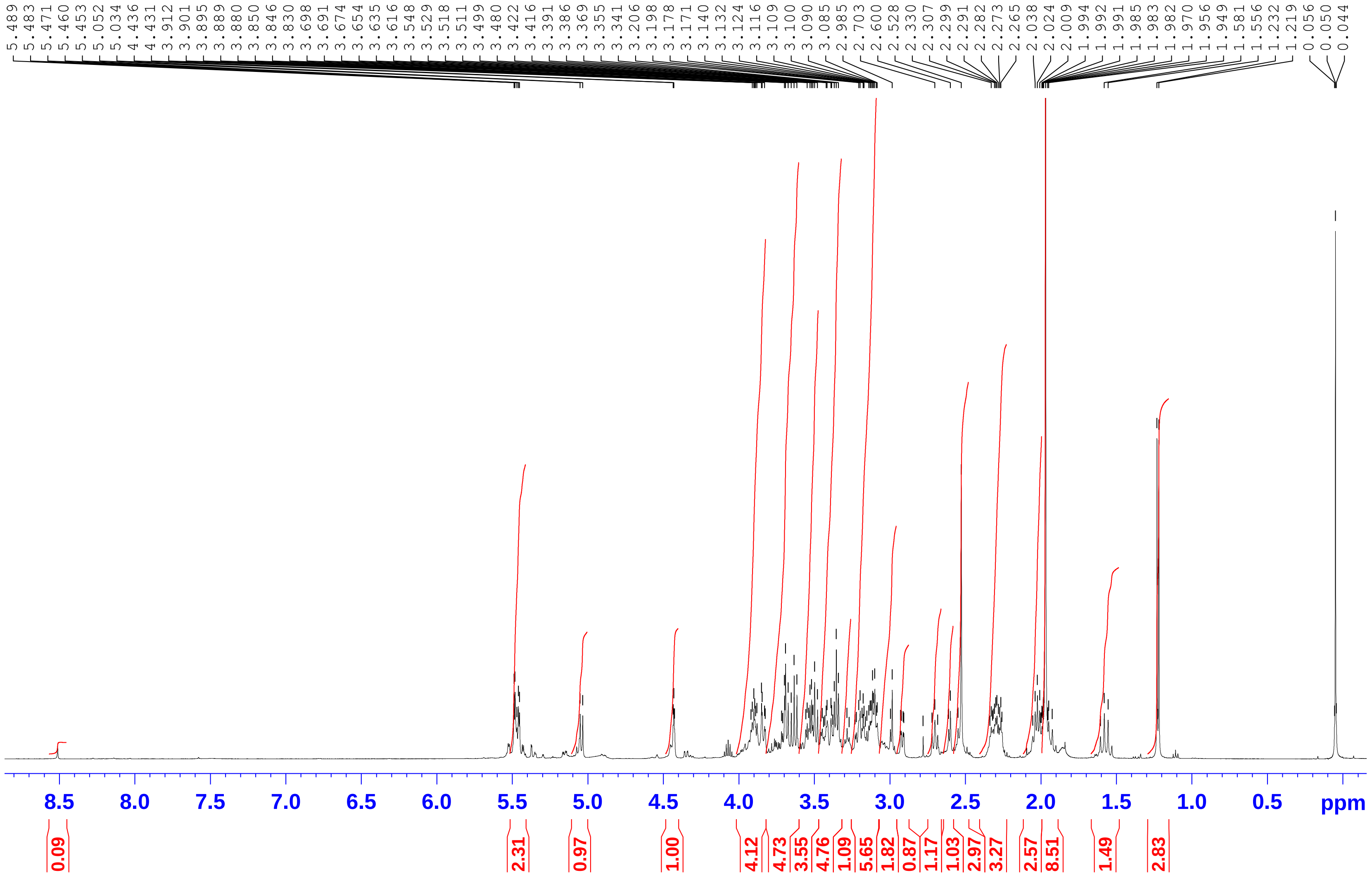

Figure S33. ^{1}H NMR (500.2 MHz, D_2O) spectrum of 6″-(3-guanidinpropil-1-amino)-6″-deoxyapramycin **8b**

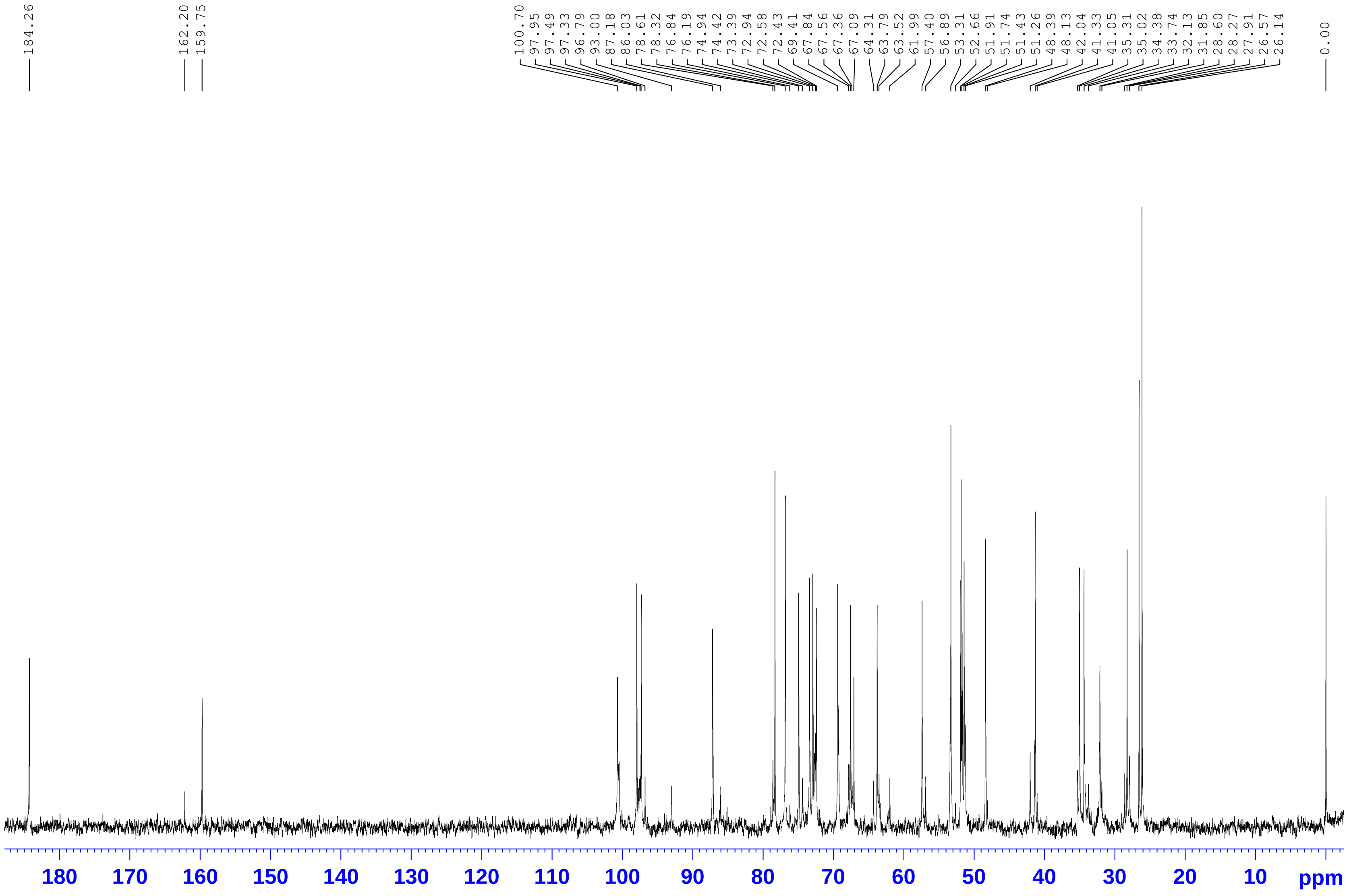

Figure S34. ^{13}C NMR (125.8 MHz, D_2O) spectrum of 6″-(3-guanidinpropil-1-amino)-6″-deoxyapramycin **8b**

SK-204 D2O COSY

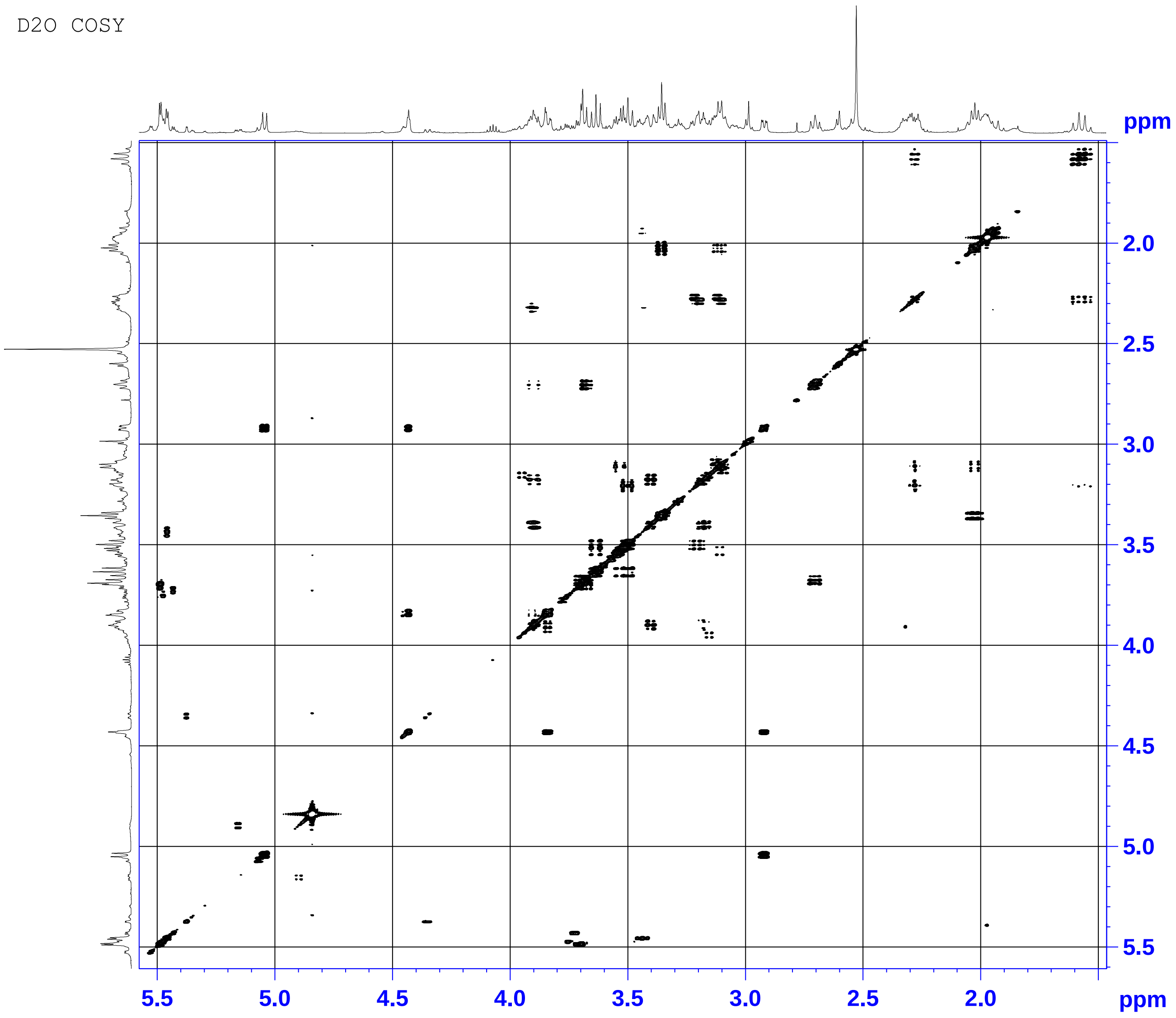

Figure S35. COSY NMR (D_2O) spectrum of spectrum of 6″-(3-guanidinpropil-1-amino)-6″-deoxyapramycin **8b**

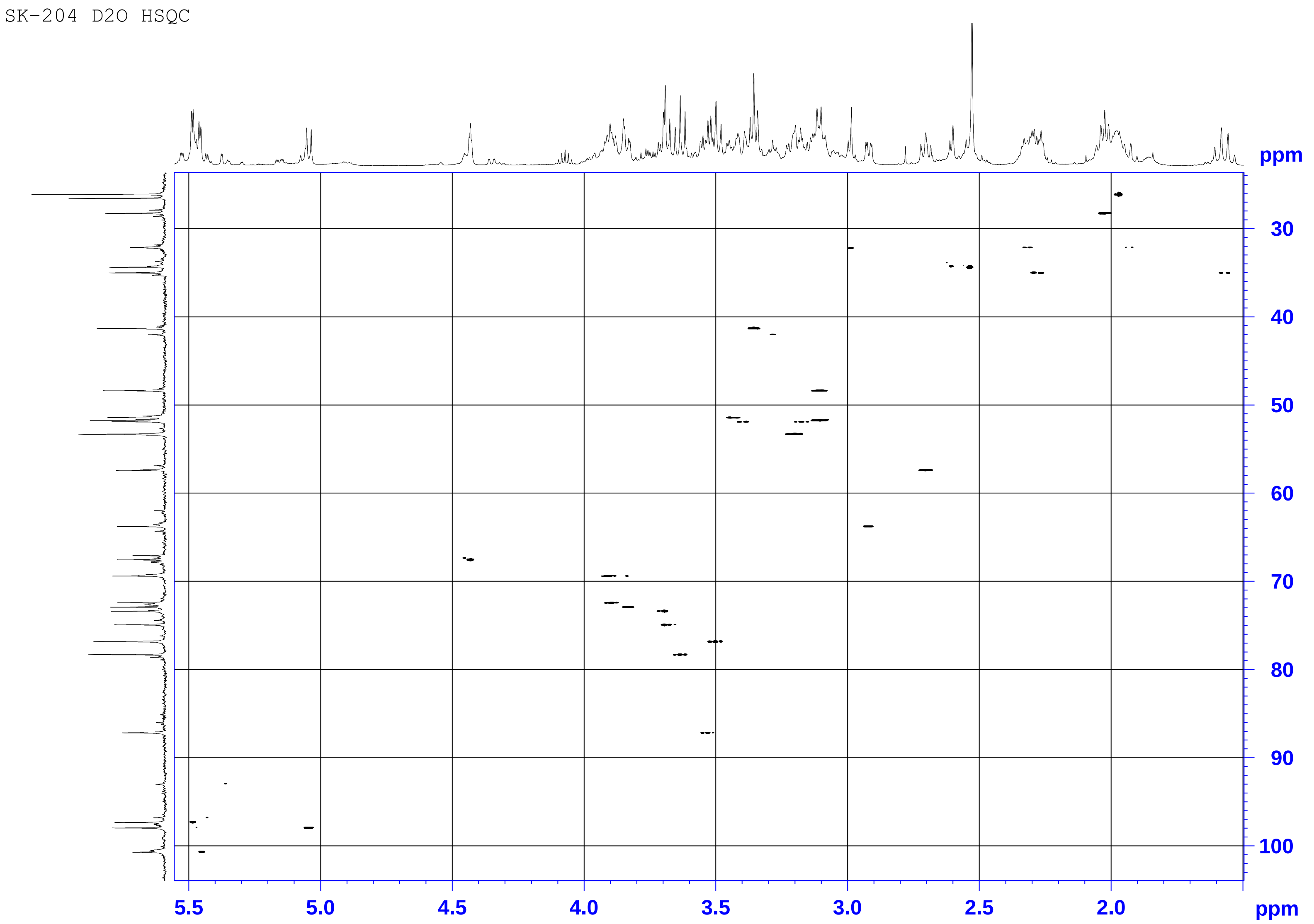

Figure S36. HSQC NMR (D_2O) spectrum of 6″-(3-guanidinpropil-1-amino)-6″-deoxyapramycin **8b**

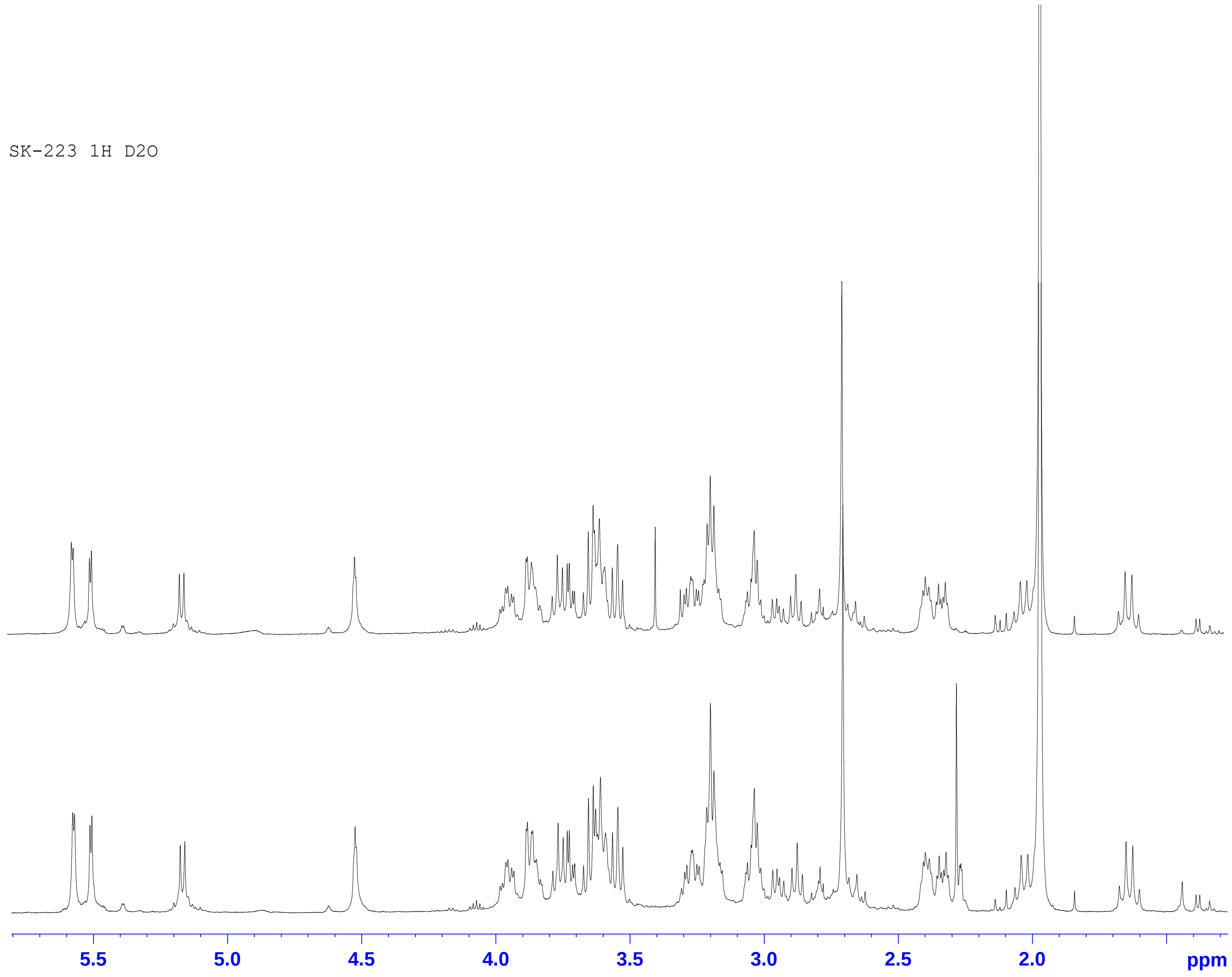

Figure S37. ¹H NMR spectrum of compound **5a** (SK-223). The top spectrum was recorded 3 months after the bottom one.
